# A computational method for three-dimensional reconstruction of the microarchitecture of myometrial smooth muscle from histological sections

**DOI:** 10.1371/journal.pone.0173404

**Published:** 2017-03-16

**Authors:** E. Josiah Lutton, Wim J. E. P. Lammers, Sean James, Hugo A. van den Berg, Andrew M. Blanks

**Affiliations:** 1 Cell and Developmental Biology, Division of Biomedical Sciences Warwick Medical School, Coventry, United Kingdom; 2 Bioengineering Institute, Auckland University, Auckland, New Zealand; 3 Department of Physiology, College of Medicine & Health Sciences, UAE University, Al Ain, United Arab Emirates; 4 Department of Pathology, University Hospitals Coventry and Warwickshire (UHCW), NHS Trust, Coventry, United Kingdom; 5 University of Warwick, Coventry, United Kingdom; PreTel Inc, UNITED STATES

## Abstract

**Background:**

The fibrous structure of the myometrium has previously been characterised at high resolutions in small tissue samples (< 100 mm^3^) and at low resolutions (∼500 *μ*m per voxel edge) in whole-organ reconstructions. However, no high-resolution visualisation of the myometrium at the organ level has previously been attained.

**Methods and results:**

We have developed a technique to reconstruct the whole myometrium from serial histological slides, at a resolution of approximately 50 *μ*m per voxel edge. Reconstructions of samples taken from human and rat uteri are presented here, along with histological verification of the reconstructions and detailed investigation of the fibrous structure of these uteri, using a range of tools specifically developed for this analysis. These reconstruction techniques enable the high-resolution rendering of global structure previously observed at lower resolution. Moreover, structures observed previously in small portions of the myometrium can be observed in the context of the whole organ. The reconstructions are in direct correspondence with the original histological slides, which allows the inspection of the anatomical context of any features identified in the three-dimensional reconstructions.

**Conclusions and significance:**

The methods presented here have been used to generate a faithful representation of myometrial smooth muscle at a resolution of ∼50 *μ*m per voxel edge. Characterisation of the smooth muscle structure of the myometrium by means of this technique revealed a detailed view of previously identified global structures in addition to a global view of the microarchitecture. A suite of visualisation tools allows researchers to interrogate the histological microarchitecture. These methods will be applicable to other smooth muscle tissues to analyse fibrous microarchitecture.

## Introduction

Smooth muscle tissue has a filamentous network structure that is essential to its electrophysiological and mechanical function [1]. It is therefore imperative to determine this structure in order to understand and control the behaviour of such tissue. The human myometrium is composed of smooth muscle cells, organised at the lowest level in small bundles of width 200–400 *μ*m [2], and at higher levels in various structures which range from clear organisation to seeming disorder [3]. In order to gain a thorough understanding of these structural components, it is necessary to obtain a reconstruction of the fibrous structure at a sufficiently high resolution to capture this microarchitecture.

Weiss *et al.* [3] achieved whole uterus visualisation using diffusion tensor magnetic resonance imaging (DT MRI), elucidating the fibre structure of the myometrium. However, detailed DT MRI investigation at this scale is limited by the relatively low resolution available for smooth muscle (∼0.5 mm), due to the low signal-to-noise ratio [4]. Higher-resolution capture of the structure of the rat heart using contrast-enhanced MRI has been achieved by Gilbert *et al.* [5] using a 9.4 T magnetic field, but larger tissue masses are not readily captured in this manner. Another technique for visualisation of tissue is micro-computed tomography (micro-CT). Whilst this technique is best suited to the imaging of hard tissues such as bone, it has been adapted for high-resolution capture of cardiac tissue [6]. One disadvantage of both MRI and micro-CT is that there is no direct or reliable correspondence with the detailed histology that is observable in serial sections.

The methods used in the present paper involve reconstructing the smooth muscle tissue from serial sections, thus providing a direct correspondence between the reconstruction and the histological sections. One major challenge encountered with this method of reconstruction is the registration of subsequent slides. One method of avoiding this issue is to perform staining and image capture while the tissue is embedded in paraffin [7]. This technique involves the manual application of stains, image capture, and removal of each layer of tissue, which is time consuming, and would be impractical for large tissue samples. Manual registration of serial sections has previously been performed extensively [8]. Again, this is time-consuming and not practical for large tissue samples. Automated registration of tissue based on the Fourier transform of the images does not rely on specific features of the tissue and hence enables registration of any tissue with sufficient heterogeneity [9, 10]. Alternatively, where unique features are present in multiple slide images, these features can be aligned to register these images [11]. These feature-based techniques are advantageous for tissue-specific registration methods because they can utilise structures which are characteristic of the given tissue. Of particular interest in the present paper are registration methods based on the cell nuclei. Can *et al.* [12] used such a technique to register slides by aligning nuclear images extracted from the histological slides. Another example of such a technique developed by Weiss *et al.* [13] uses nuclear density as a feature for registration.

A small portion (80 mm^3^) of the human myometrium has previously been reconstructed from histological sections by Young and Hession [2]. This reconstruction provides information on the basic structures of the myometrium, which is the subject in the present study. The scale of the smallest fibrous structures (∼300 *μ*m) dictates the resolution required to observe these structures.

The technique described here enables the *in silico* reconstruction of the fibrous structure of myometrial smooth muscle from histological sections. In particular, this reconstruction represents the tissue as a weighted direction field at a resolution of ∼50 *μ*m per voxel length, where the weighting represents density and heterogeneity of the smooth muscle cells. This enables a systematic visualisation of three-dimensional structures within the myometrium that can be directly referred back to the original histological slides, thus providing a relevant histological and, conceivably, physiological or anatomical, context for any structural features identified. The present paper uses a specialised feature-based registration technique that takes the local orientation of nuclei as a feature for alignment. The main advantage of this approach is the precise registration of local structures based on the direction of the bundles. We compare the *in silico* structure thus obtained to the original tissue in order to verify the accuracy of the representation.

## Materials and methods

*In silico* tissue microarchitecture was established through histological inference of serial sections of the tissue, using computational image analysis on the histological sections to determine fascicular direction. The final result is a representation of the fascicular directions in the tissue samples at a resolution of ∼50 *μ*m per voxel length. The computational process was semi-automated, with the only manual input required being verification of the integrity of slides after registration.

**Fig 1:**
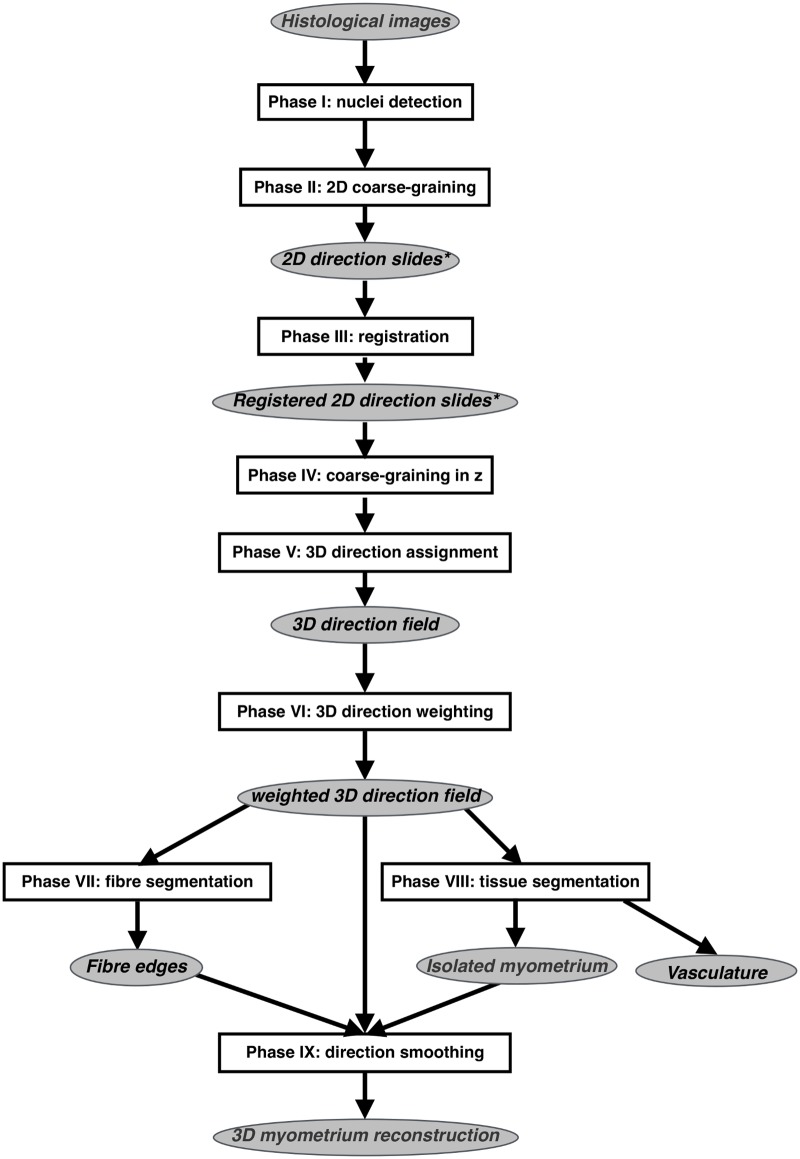
Processing steps of the reconstruction algorithm. All steps in this algorithm are fully automated, with the exception of **Phase III**, which requires some manual verification for the purposes of quality control. Slides marked with an asterisk were also used in the verification of the reconstruction’s faithfulness to the original slides, being subjected to two-dimensional fascicular segmentation (**Phase VII**) and direction smoothing (**Phase IX**). An additional step (**Phase X**) was used to measure bundle widths, which enabled both comparison of registered and unregistered slides and analysis of the reconstructed microarchitecture. Vasculature was also identified as a product of tissue segmentation (**Phase VIII**).

The sequence of steps used to generate the reconstructions from serial sections is shown in Fig 1. All code used in this process is available in the supporting information (S1 and S2 Files).

### Ethics statement

All procedures on human tissue were conducted within the guidelines of The Declaration of Helsinki and were subject to local ethical approval (REC-05/Q2802/107; approval accorded by the West Midlands Coventry and Warwickshire Research Ethics Committee). Prior to surgery, informed written consent for sample collection was obtained.

The experimental protocol for the use of rat tissue was approved by the Animal Research Ethics Committee, Faculty of Medicine & Health Sciences, United Arab Emirates University (institutional ethical approval: AE/03/30).

### Animal housing

Rats were housed in standard cages and kept in a 12-hour light-dark cycle at 20 C°. They were fed standard rat chow and had free access to water. Rats were fasted for 12 hours before the experimental procedures but had free access to water. Anesthesia was induced and maintained with ether inhalation. The experimental protocol was approved by the local ethics committee.

### Tissue preparation

**Fig 2:**
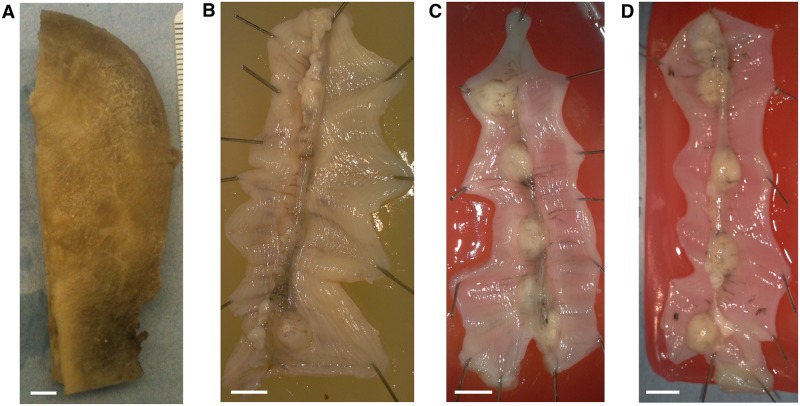
Tissue samples. **A**: Human uterus tissue block, positioned with fundus at the top of the image and cervix at the bottom. Transverse sectioning was performed in the orientation shown here, starting at the top (nearest) surface, up to a depth of 3.5 mm. **B**–**D**: Rat uteri processed, positioned with ovarian end at the top of the image and cervical end at the bottom. Each rat uterus was cut along the antimesometrial border, preserving the mesometrial border where implantation sites can be seen as yellow bumps near the middle of the tissue. All rat samples were sectioned in the orientation displayed, starting at the bottom (farthest) surface, and continuing through the entirety of the tissue. White scale bars represent 5 mm.

Three rat uteri and one human uterus were investigated. The human uterus was non-pregnant and a sample of dimensions 7 cm × 3 cm × 0.35 cm was taken from the tissue for processing. This sample included the length of the organ, as shown in Fig 2A. The full length, however, was slightly too large to fit on the slides used. For this reason the cervical (caudal) end was clipped during sectioning. The rat uteri were obtained in following manner. Three virgin Wistar rats were time-mated, and pregnancy dated as day 0 of gestation if the sperm cells were observed in the vaginal lavage the next morning. Rats were euthanized by graded CO_2_ inhalation and the uterine horns were rapidly excised via a midline incision of the abdomen on day 20 for two samples (Fig 2B and 2C) and day 19 for the third (Fig 2D). The uterine horns were opened longitudinally along the antimesometrial border and embryos removed and pinned with the serosal side facing upwards, to a final dimension of approximately 20 mm×50 mm.

The tissue samples were fixed in formalin, embedded in paraffin, and sliced into serial sections 5 *μ*m thick. The slides were labelled with a unique identifier for the tissue block and numbered according to the sectioning order. Some sections produced were highly distorted through creasing or tearing and were therefore deemed unfit for processing and were discarded. Each such section was numbered according to the above convention, although no physical slide was present, to preserve the physical distance between slides. After discarding these slides, the number of remaining slides subjected to the following analysis was 284 in the human tissue block, and 221, 237, and 201 in the rat tissue blocks. This corresponded to discarding 64% of the slides in the human tissue block and 44%, 43%, and 52% of the slides in the rat tissue blocks. This resulted in no loss of information because the tissue was oversampled by sectioning the tissue at a thickness of 5 *μ*m. Slides were also discarded during the registration algorithm (**Phase III**) if they fail to accurately register.

All remaining slides were subsequently stained with haemotoxylin and eosin (H & E) and scanned. The scanning resolution for slides in one of the rat tissue samples (Fig 2B) was 0.465 *μ*m×0.465 *μ*m per pixel, and slides from all other tissue samples were scanned at 0.371 *μ*m×0.372 *μ*m per pixel.

The scanned slides were in Mirax format, and were converted into TIFF files using either the Mirax Pannoramic Viewer [14] or a combination of Vips [15], OpenSlide [16], and bigTIFFTools [17]. The conversion produced a set of tiles of size 2048×2048 pixels for each slide, where each tile represented a square of the overall image without overlap. These tiles provided the input for the ImageJ plugin described in the following section.

### Phase I: Two-dimensional extraction of cell orientation

The orientation of a myometrial smooth muscle cell is well-approximated by the orientation of its nucleus [18], which implies that the cell nuclei are a good indicator of the direction of the bundle in which the observed cell is embedded. Accordingly, the image analysis process began with the acquisition of shape data for the nuclei. This process was fully automated and was performed by a bespoke ImageJ plugin.

**Fig 3:**
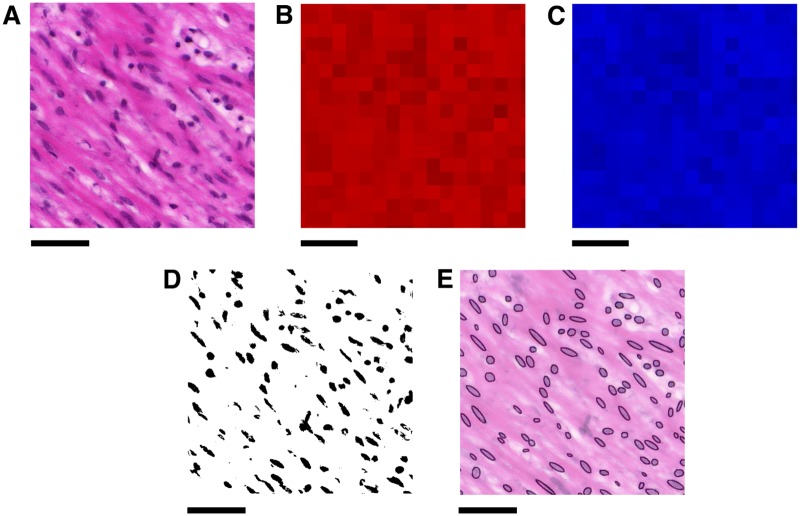
Extracting nuclei from histological slides. **A**: A 500 × 500 pixel region of tissue. **B** & **C**: Local red and blue thresholds respectively, found by considering the average values within 32 × 32 pixel regions. **D**: Binary image obtained by applying these thresholds to the original image **A**. **E**: Ellipses found by applying the ImageJ “Analyze Particles” function [20] to **D**, superimposed onto a lower intensity copy of **A**, showing the nuclei isolated from the rest of the tissue. Scale bars represent 50 *μ*m.

To isolate the nuclei from the rest of the image, a thresholding procedure was used, as summarised in Fig 3. The H & E staining colours nuclei purple and the remaining tissue pink [19]. Accordingly, the thresholding algorithm targeted red and blue colour values in the RGB colour space of the images.

Variation in staining of slides necessitated the use of different thresholds for each slide. An additional difficulty was variation of the contrast between the nuclei and the surrounding tissue within each slide, as can be seen in Fig 3A, so that threshold values needed to be defined locally within each slide. In view of these considerations, each slide was divided into regions of 32 × 32 pixels (12 × 12 *μ*m^2^ high-resolution, 15 × 15 *μ*m^2^ low-resolution). This size was chosen as it is approximately twice the width of a typical nucleus (5–7 *μ*m, or 10–20 pixel lengths depending on resolution) and each region containing a nucleus will therefore contain a portion of the surrounding tissue.

The threshold for each colour was determined by taking the average value of the region: for red values, the upper threshold was set at 5/6 times the average value (Fig 3B). The nuclei were highlighted by setting the threshold slightly below the average red value; this also removes the surrounding tissue from the binary image. The blue values have an upper threshold equal to the average value (Fig 3C), which serves to reduce the noise in the resulting binary image, while preserving pixels in the nuclei. The resulting binary image is shown in Fig 3D.

The binary images obtained contain the approximate shapes of the nuclei as seen within the plane. Next, the images were analysed using the ImageJ “Analyze Particles” function [20]. This function fits an ellipse to each nucleus, as shown in Fig 3E. The function also allows the ellipses to be filtered by size, which enables further noise reduction by eliminating ellipses which are outside the feasible size range of nuclei. Ellipses with area smaller than 40 pixels (5.5 *μ*m^2^ high-resolution, 8.6 *μ*m^2^ low-resolution) in size were discarded as noise, while ellipses larger than 600 pixels (82.6 *μ*m^2^ high-resolution, 129.7 *μ*m^2^ low-resolution) in size were discarded on the assumption that these corresponded to clusters of nuclei, which would not provide accurate shape data for the purposes of determining fascicular direction.

**Fig 4:**
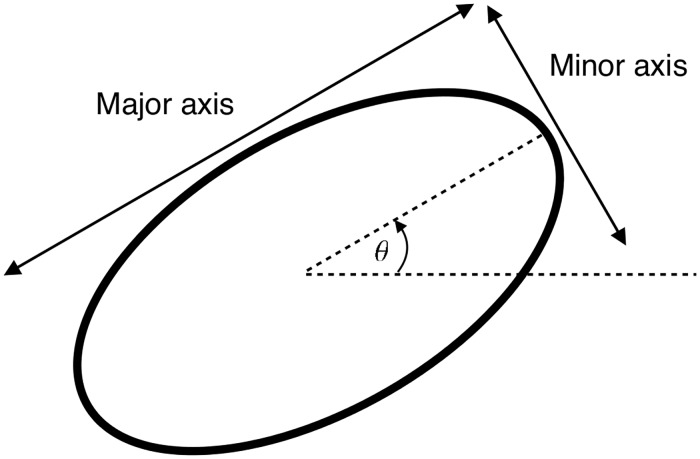
Shape characteristics of an ellipse. Aspect ratio was calculated as major axis divided by minor axis.

The length and orientation of the major axis of the ellipse was defined by the largest diameter of the nucleus, whereas the minor axis was fixed by setting the area of the ellipse equal to that of the nucleus [20]. For each of these ellipses, the position, aspect ratio, area, and angle of the major axis from the *x*-axis were recorded as illustrated in Fig 4.

### Phase II: Coarse-graining of nuclear orientation

The nuclear shape data indicates the orientation of individual cells in the tissue. This orientation gives an indication of the direction of the bundle in which the cell was embedded; however, the orientation of an individual cell deviates from the bundle direction and therefore does not directly correspond to the bundle direction. Thus, to determine the fibrous structure, a coarse-graining step was required. For this reason, regional averages were calculated to reduce the data set. Each slide was divided into regions of size 128 × 128 pixels (47.5 *μ*m×47.5 *μ*m high-resolution, 59.5 *μ*m×59.5 *μ*m low-resolution). All ellipses with centres within a given region were considered as being contained within that region.

The ellipses used to generate this regional representation were filtered by size: ellipses within the size range 10–60 *μ*m^2^ were considered to be smooth muscle nuclei. This refinement of the earlier size filter removed smaller cells, such as red blood cells, and larger clusters from the calculations. Ellipses outside this size range were used later in the processing pipeline: the smaller size range was used in detecting vasculature, while the full range of sizes was used for detecting placental tissue.

The ellipses within a region were categorised as being *planar*, i.e. nucleus lying in the plane of the slide, *vertical*, i.e. nucleus lying vertical to the plane of the slide, or else *indeterminate*. A long thin ellipse is more likely to represent a nucleus in the cut-plane than a short round one. Accordingly the aspect ratio was used as a criterion to distinguish between these categories: nuclei with aspect ratio greater than 2.0 were classified as *planar*, nuclei with aspect ratio less than 1.6 were classified as *vertical*, and nuclei with aspect ratio between 1.6 and 2.0 were considered indeterminate, and were included in both *planar* and *vertical* categories.

**Fig 5:**
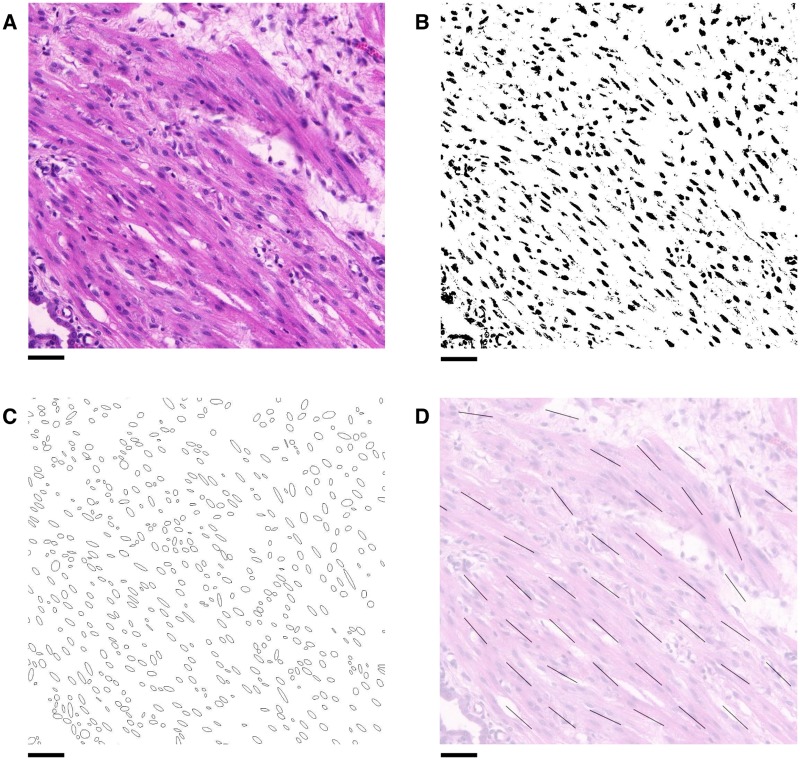
Obtaining directional data from histological images. **A**: Example area of an H & E stained slide taken from rat myometrium. **B**: Image obtained after applying the thresholding procedure to **A**, yielding a binary image with nuclei shown in black. **C**: Ellipses approximating the nuclei found using the ImageJ “Analyze Particles” function on **B**. **D**: Directional data obtained by averaging directions of ellipses in **C**, shown in comparison with the original image **A**. Scale bars represent 50 *μ*m.

In this manner, regions are categorised as being *planar*, *vertical*, or *empty*. If a region contained fewer than two ellipses, it was categorised as being *empty*. If more than 5/9 of the ellipses within a region were classified as *vertical*, the region was classified as *vertical* and no planar direction was assigned to the region. Otherwise, the region was classified as *planar* and a direction for the region was also assigned: the direction of a region was defined to be the median angle of all ellipses in the region. The median was used rather than the mean as the number of ellipses within a region was generally small, which renders the mean highly sensitive to outliers, whereas the median is more robust. The interquartile range of the angles within a given region was also taken; if this was greater than 45° then the direction was deemed unreliable, and consequently the region was marked as *vertical* and therefore was not assigned a direction. An example of the resulting directional data is shown in Fig 5.

In the following analysis, the regions used in this coarse-graining step are referred to as pixels, since they represent the indivisible unit of the coarse-grained images. In this regional representation, each pixel is assigned the category (*planar*, *vertical*, or *empty*), nuclear count, and direction (where appropriate) of the region of tissue that it represents. All coarse-grained slides were padded with *empty* pixels on all sides such that the images all had 800 pixels width and 1600 pixels height. This padding was performed to ensure that all slide data remained within the image frame after registration (**Phase III**). When it is necessary to refer to the original histological images, distances will be described in terms of real-world measurements, thereby confining the term pixel to represent the coarse-grained image pixels.

### Phase III: Image registration

Registration of the histological slides is an absolute prerequisite for the construction of an *in silico* representation of the three-dimensional structure of the tissue. Two types of registration were sequentially applied to achieve this. The first is a rigid registration, where each slide is translated and rotated based on a single transformation that is applied to the whole slide, so that the slide maintains its original shape. The second is an elastic registration, where a grid of points is laid over the slide and each point can be subjected to a different transformation, which allows the slide to adjust its shape in aid of improved correspondence of tissue features.

In both registration processes a generalised form of the Hough transform was used [21]. Registration of a shape *S*_1_ to a reference shape *S*_0_ involves finding a transformation *T* such that the transformed shape *T*(*S*_0_) approximates the reference shape *S*_1_. This was achieved by searching for a transformed image of the shape *S*_1_ in the reference shape *S*_0_. In particular, the Hough space for this shape detection is the three-dimensional space given by the rotation angle *θ* and translation vector *t* such that for a shape *S*

$$\begin{array}{c} T\left(S\right)=M_{\theta }S+t, \end{array}$$

where *M*_*θ*_ is the rotation matrix of *θ*, defined by

$$\begin{array}{c} M_{\theta }≔\left(\begin{array}{cc} \cos\theta & \sin\theta \\ -\sin\theta & \cos\theta \end{array}\right). \end{array}$$

This Hough space was generated by performing the transformation *T* to *S*_1_ and recording the number of transformed points in *S*_1_ that match points in *S*_0_. Applying this process to a range of transformations yielded a density plot for the transformation parameters, such that the optimal transformation in this range is located at the point of highest density.

In principle, this technique enables registration of any two shapes. However, mapping each pair of points to the Hough space in this fashion is impractical for shapes with a large number of points. For this reason recourse must be taken to additional techniques to reduce the computational effort required, as described in the rigid and elastic registration sections below.

#### Rigid slide registration

**Fig 6:**
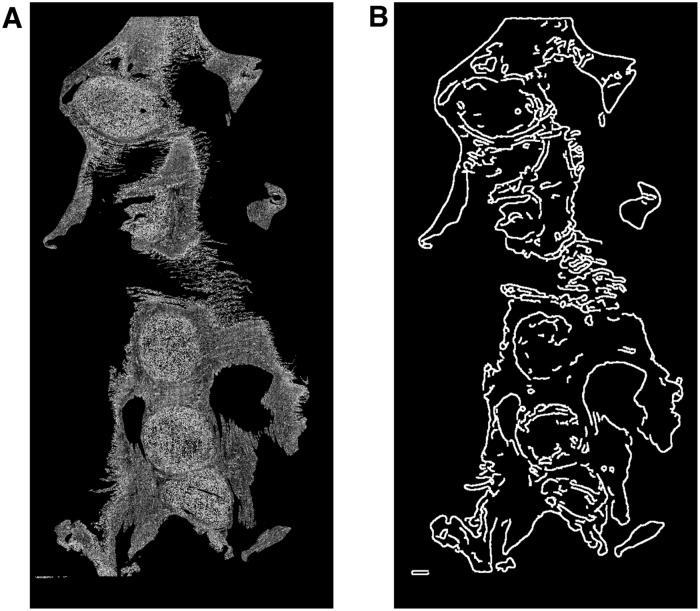
The edge detection process for whole slides. **A**: Grey image representing the nuclear count assigned to each pixel. **B**: The edge image produced using a Canny edge detection algorithm on **A**.

The rigid registration was performed by letting the edge of the tissue in each slide as the shape to be used as the input for the generalised Hough transform. To obtain the edge image of a given slide, a greyscale image was generated by considering the nuclear density. Each pixel was assigned a value equal to its nuclear count calculated in **Phase II**. These pixel values were then scaled up by the constant factor given in Table 1 to improve contrast for visualisation. The resulting image can be seen in Fig 6.

**Table 1:** Parameter values for edge detection.

Parameter	value
Count scale factor	20
Gaussian standard deviation	3 pixels
Sobel window radius	1 pixels
Upper hysteresis threshold	50
Lower hysteresis threshold	30

The Canny edge detector uses Gaussian smoothing, a Sobel differencing operator, and hysteresis thresholding using the parameters specified here.

A Canny edge detection algorithm [22] was subsequently applied to these images to produce images of the edges of the tissue (Fig 6B), which were then subjected to the rigid registration procedure. Parameter values for the Canny edge detector are given in Table 1.

Computing a full Hough space to find the optimal transformation would require a large number of calculations and would hence take an excessive amount of time to perform. In order to improve computational efficiency, the transformation was split into a translation and a rotation, and the Hough density optimisation was performed for each separately. The registration algorithm alternated, in an iterative fashion, between rotating and translating the image to find the optimal transformation. This sequence of iterations was subdivided into 7 *transformation steps*, where a maximum of 10 such iterations were performed in each step. Furthermore, with each step the range of possible translations and rotations was decreased, as described below.

Let *E*_1_ denote the set of edge points in the image to be registered and *E*_0_ denote the set of edge points in the reference image. For each translation step, the Hough space is the density plot of the translations

$$\begin{array}{c} t=q-p, \end{array}$$

for all *q* ∈ *E*_0_ and *p* ∈ *E*_1_. This Hough space was bounded for the sake of computation, and the bounds for the Hough space were narrowed with each transformation step, to further improve efficiency. The sequence of bounds on translation in each direction is given by

$$\begin{array}{c} \pm \frac{W}{2^{i}}, \end{array}$$

where *W* = 800 is the image width and *i* is the transformation step number from 1 to 7.

**Fig 7:**
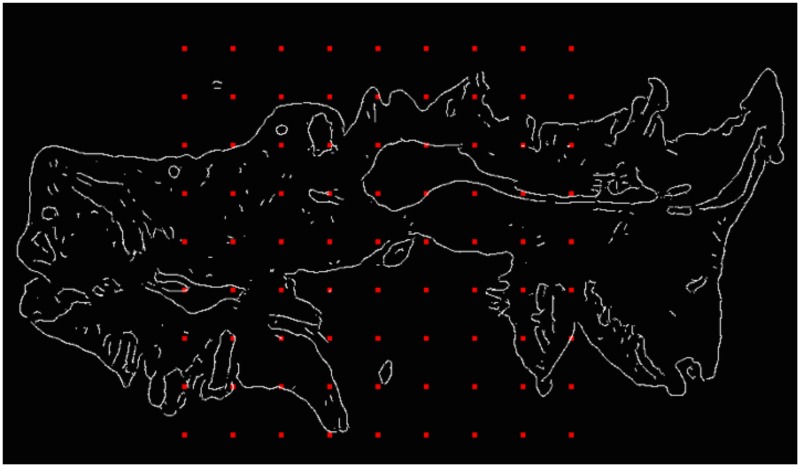
Centres of rotation for rigid slide registration. Each red point is used as a centre of rotation for the algorithm. The size and spacing of this grid is varied depending on transformation step number.

For each rotation step, the Hough space is the density plot of rotation angles *θ* with

$$\begin{array}{c} M_{\theta }p=q, \end{array}$$

where *M*_*θ*_ is the rotation matrix of *θ*, *p* ∈ *E*_1_, and *q* ∈ *E*_0_ as in the translation step. This formulation of rotation specifically takes the centre of rotation at the origin of the image space. To improve performance a range of rotation centres *C* was used, as illustrated in Fig 7. At each rotation step, the angle of rotation was optimised for all centres in *C* before proceeding to the following translation step. This grid of points was dependent on transformation step number: the central coordinate of the image *c*_0_ was included in each such grid, and all other grid points were of the form

$$\begin{array}{c} c_{0}+50i\left(x,y\right), \end{array}$$

where *i* is the transformation step number, and *x* and *y* are integers. This grid was confined to the area within 200 pixels from *c*_0_ for the first transformation step, and to within the bounds of the image in all later transformation steps. This reduction in grid points reduced the number of calculations required for each transformation step, and hence reduced the computation time.

**Fig 8:**
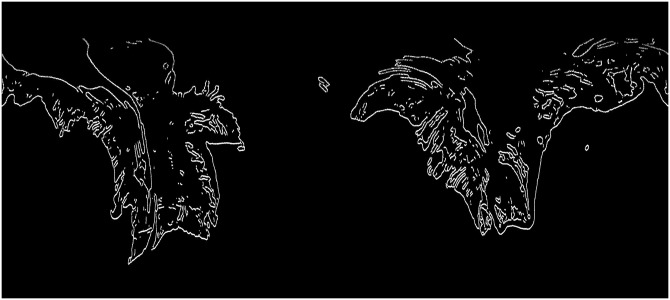
Polar transform of an edge image. Polar transform of the edge image given in Fig 6B.

For each *c* ∈ *C*, the Hough space is the density plot of rotation angles *θ* with

$$\begin{array}{c} M_{\theta }\left(p-c\right)+c=q, \end{array}$$

with *M*_*θ*_, *p* ∈ *E*_1_, and *q* ∈ *E*_0_ as above. Optimising for each centre of rotation in turn enabled a faster overall optimisation. To calculate the values for the rotational Hough space, the images were converted to polar coordinates, as shown in Fig 8. For each centre of rotation *c* the polar space was defined by taking *c* as the origin.

A translation along the angle axis in the polar space is equivalent to a rotation about *c* in Cartesian space. Thus the Hough space is the density plot of the set of angles *θ* such that

$$\begin{array}{c} r+\left(\begin{array}{c} 0\\ \theta \end{array}\right)=s \end{array}$$

where *r* and *s* are contained in the polar transforms of *E*_1_ and *E*_0_ respectively. As was the case with the translation Hough space, the angle Hough space was bounded, and this bound was reduced with each transformation step. In particular, the sequence of bounds on the angle of rotation was given by

$$\begin{array}{c} \pm 180^{{}^{\circ}},\pm 10^{{}^{\circ}},\pm 5^{{}^{\circ}},\pm 2.5^{{}^{\circ}},\pm 1.25^{{}^{\circ}},\pm 1^{{}^{\circ}}. \end{array}$$

This sequence starts at ±180° to allow for the possibility that a slide was rotated by ∼180° during the sectioning process.

The iteration procedure for each transformation step is as follows:

1. Compute the optimal translation and translate image.
2. For each centre of rotation, compute the optimal rotation and rotate image.
3. If no rotation or translation changes the image or 10 iterations have occurred, end, else go to (1).

This iterative loop was repeated 7 times, with each transformation step reducing the range of translations and rotations as described above. This technique was applied to all pairs of adjacent slides sequentially in the stack, yielding a set of slides that were globally aligned with each other. The elastic registration procedure was subsequently applied to this stack, as described in the following section.

#### Elastic slide registration

The physical process of sectioning distorts the tissue on a local level. In order to correct for this distortion, a second registration algorithm was applied that allows a limited amount of elasticity within the slides. The elastic registration process enabled the refinement of the rigid registration described above, by applying the generalised Hough transform to match areas of a slide to a reference slide. The regional representation of the slides was used to provide the shape data for the generalised Hough transform. The registration algorithm specifically registers *planar* pixels, while *vertical* and *empty* pixels are considered to be indistinguishable for the purposes of registration, both being regarded as *empty*.

**Fig 9:**
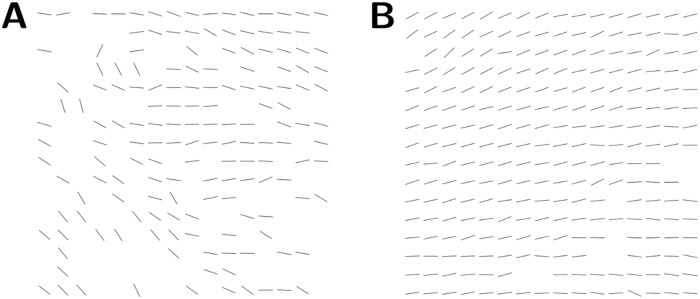
The varying degrees of heterogeneity within a slide. The lines represent directions of *planar* pixels. Each image shows the planar directions in a 16 × 16 pixel (approx. 1 × 1 mm^2^) tile. **A**: An example of a tile of high heterogeneity. The large amount of empty space and variation in fascicular direction allows for a precise registration of the tile. **B**: An example of a tile of low heterogeneity. Here the tile comprises vectors with little variation in direction. This homogeneity can readily lead to an unreliable registration, and accordingly, the area of interest needs to be expanded to obtain a higher level of heterogeneity.

The distortion in a slide cannot be completely eliminated, since attempting to do so would inevitably lead to each slide being transformed into a near copy of the reference slide to which it was registered. To avoid this extreme situation, the elastic registration was locally rigid: while distances between points far apart in a slide could be modified by the registration, points in close proximity to each other maintained a fixed distance during registration. The area covered by this local rigidity was determined on the basis of the spatial heterogeneity of the neighbourhood, as follows. Consider the two areas illustrated in Fig 9. Fig 9A shows a relatively heterogeneous region of tissue, which translates into a limited range of potential transformations onto the reference slide. On the other hand, Fig 9B shows a more homogeneous region; this homogeneity might allow this region to be transformed to many regions on the reference slide and still appear to be properly aligned.

**Fig 10:**
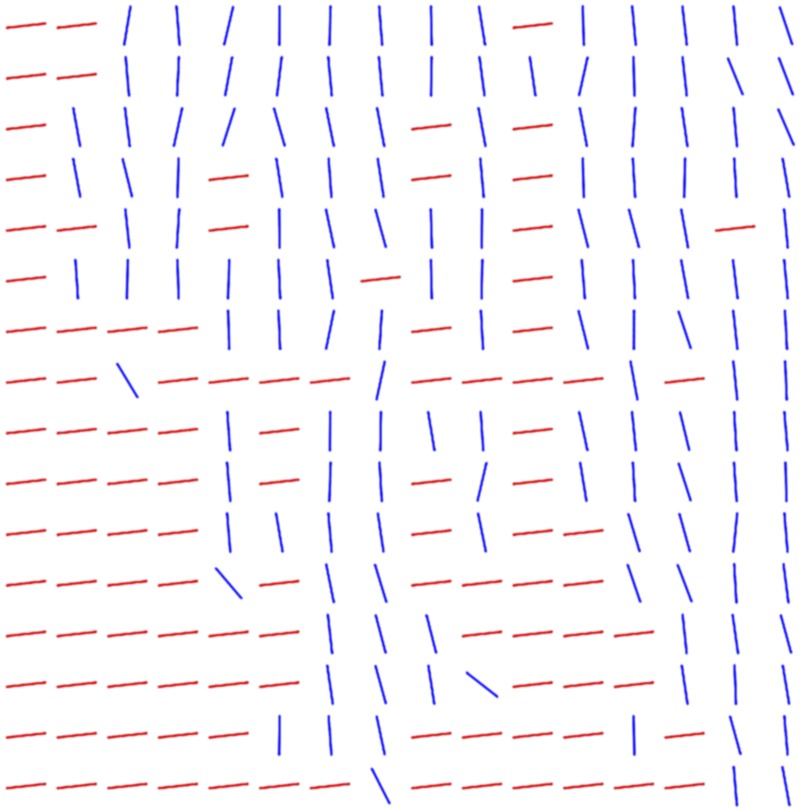
Directions used for measuring heterogeneity. Blue lines indicate the directions of the *planar* pixels, while the red lines indicate non-*planar* pixels, which are represented in the heterogeneity measure using the direction perpendicular to the mean direction of the *planar* pixels, as shown.

The overall heterogeneity of an area is characterised by the combination of the heterogeneity of pixel classifications and the heterogeneity of directions assigned to *planar* pixels. This combined heterogeneity was quantified as follows. For an area of *N* pixels, let *N*_*p*_ be the number of *planar* pixels contained in the area, with direction angles ${\left\{\theta_{i}\right\}}_{i=1}^{N_{p}}$. Define the sequence of angles $S={\left\{\phi_{i}\right\}}_{i=1}^{N}$ such that

$$\phi_{i}=\{\begin{array}{ll} \theta_{i} & \text{if }i\leq N_{p}\\ \bar{\theta }+90{}^{\circ} & \text{if }i>N_{p} \end{array}$$

where $\bar{\theta }$ is the mean angle of the *planar* pixels in the area. This sequence can be thought of as the set of planar angles in the area with all non-*planar* pixels being filled in with the direction perpendicular to the mean direction of the *planar* pixels, as illustrated in Fig 10. For areas where a majority of the pixels are categorised as *planar*, the addition of these angles to the sequence increases the heterogeneity of *S*. Conversely, areas where non-*planar* pixels are in the majority, most of the angles in *S* are of the same value, and therefore more non-*planar* pixels would decrease the heterogeneity of *S*. This modified sequence of angles accounts for heterogeneity of categorisation by the above reasoning, while heterogeneity of the planar directions is accounted for by the inclusion of the planar angles in the sequence. The heterogeneity of the area is thus defined to be the variance of *S*.

The level of heterogeneity was used to determine how large an area around a 16 × 16 pixel tile was required for registration. If the angle variance was below (45°)^2^, the area being registered was expanded to include neighbouring tiles. This expansion continued until the required level of heterogeneity was achieved, to minimise the risk of mapping to spurious areas on adjacent slides.

The following steps require a suitable quantitative expression that indicates how well a given area matches the reference slide for a given transformation. This comparison was expressed by an energy function. Consider an area *A* containing *N* pixels and a transformation *T*. For a pixel *p*, the transformed point *T*(*p*) will be surrounded by a set of pixels {*q*_*i*_} in the reference slide, given by

$$\begin{array}{c} q_{i}=\left\lfloor T\left(p\right)\right\rfloor +\left(x,y\right) \end{array}$$

where ⌊⋅⌋ is a rounding down of the coordinates to the next pixel, and *x*, *y* ∈ {0,1}. The pixel *p* is defined to be *matched* if, for any *q*_*i*_, either both *p* and *q*_*i*_ are non-*planar*, or both are *planar* and

$$\begin{array}{c} |a\left(q_{i}\right)-\left(a\left(p\right)+\theta_{T}\right)|<a_{\text{max}}, \end{array}$$

where *a*(⋅) is the planar angle of a pixel, *θ*_*T*_ is the angle of translation, and *a*_max_ is the maximum angle difference allowed, as given in Table 2. The energy *e* of the transformation *T* in *A* is defined to be

$$\begin{array}{c} e\left(A,T\right)≔1-\frac{N_{m}}{N}, \end{array}$$

where *N*_*m*_ is the number of pixels which are matched according to the criteria described above.

**Table 2:** Parameter values for elastic registration.

Parameter	Value
*a*_max_	20°
*e*_min_	0.2
*b*_0_ (human)	32 pixels
*b*_0_ (rat)	16 pixels

The first step in optimising the transformation of an area *A* was to determine if it requires a transformation. The area was deemed to require transformation if

$$\begin{array}{c} e\left(A,T_{0}\right)>e_{\text{min}}, \end{array}$$

where *T*_0_ is the transformation defined by no rotation and no translation and *e*_min_ is the minimum energy required to perform a transformation, as given in Table 2. This initial test reduced the computational workload by reducing the number of areas needing to be registered.

For an area requiring transformation, the directional data within this area were used for the Hough transform. To create the Hough space, the directional data in the area of interest and the corresponding data on the reference slide were taken to be the shape data. If *S*_1_ and *S*_2_ denote, respectively, the points corresponding to *planar* pixels in the area of interest and in the reference slide, the Hough space is then generated by plotting the density of the rotation and translation parameters (*α*, *t*), given by

$$\begin{array}{c} t=-M_{\theta }p+q, \end{array}$$

for all *p* ∈ *S*_1_ and *q* ∈ *S*_0_. Additionally, the Hough space was bounded by comparison of the directional data: if the pixel *p* has angle *a*_1_, and the pixel *q* (on the reference slide) has angle *a*_0_, then a further requirement is

$$\begin{array}{c} |a_{0}-\left(a_{1}+\theta \right)|<a_{\text{max}}, \end{array}$$

where *a*_max_ is the maximum difference in direction allowed, as given in Table 2. This ensures that the direction of *p*, after rotation, gives an angle which approximates that of *q*. This second constraint bounds the angle range in the Hough space corresponding to the transformation of *p* to *q*. In addition to this bound, the distance an individual pixel was allowed to be transformed and the angle of rotation were also bounded. The angle of rotation was bounded by 30°, while the bound on transformation distance was determined as follows. For an area *A* the distance bound is defined to be

$$\begin{array}{c} b\left(A\right)≔b_{0}e\left(A,T_{0}\right) \end{array}$$

where *T*_0_ is the zero transformation and *b*_0_ is a constant. The energy factor *e*(*A*, *T*_0_) in this bound means that the search for the transform is more localised when the area closely matches the reference slide, and a wider search was performed when the area did not resemble the corresponding area in the reference slide. The bound *b*(*A*) is the elasticity of the area *A*, with *b*_0_ fixing the maximum elasticity of any given area. The value of *b*_0_ was selected for a tissue block based on the cross-sectional area of the tissue; a tissue with a larger cross-section has more area to distort, and hence requires a larger amount of elasticity. This elasticity limit serves not only to reduce computational workload, but also to prevent the registration algorithm distorting the tissue through a widely inaccurate transformation. Subject to these bounds, the generalised Hough transform was implemented to obtain the optimal transformation for each area.

This process assigned a transformation to each tile within a slide. In order to ensure accuracy of each of these transformations, they were compared to those of neighbouring tiles. For a given tile, the transformations assigned to tiles situated within a local neighbourhood of the initial tile were compared to the transformation assigned to the initial tile. If one of these was categorised as superior, it replaced the original transformation. To compare transformations, the energy function was used. For a given neighbourhood, the tiles within that neighbourhood were assigned areas of interest {*A*_*i*_} and transformations {*T*_*j*_} by the process detailed above. The total energy of a transformation *T*_*j*_ over the neighbourhood is given by

$$\begin{array}{c} \sum_{i}e\left(A_{i},T_{j}\right). \end{array}$$

The transformation which minimised this sum was deemed the optimal transformation. The initial tile was assigned this optimal transformation.

The application of this comparison has two main advantages. First, it reduces the chance of a tile being assigned an incorrect transformation. Second, it allows for a wider range of transformations for an individual tile, as the range of transformations available to each tile is bounded by the local elasticity. This local elasticity differs with each tile; therefore a tile with a high level of elasticity could attain a transformation outside the range of tiles with lower levels of elasticity. By allowing each tile to potentially be assigned transformations from neighbouring tiles, tiles of low elasticity can attain transformations outside of the initial elasticity limit.

Finally, each tile was transformed based on these allocated transformations. The transformation was applied to the original nuclear data. Each data point was transformed using a weighted average of the transformations from the nearest tiles. For a nucleus at point *p*, let {*c*_*i*_} denote the set of the four closest tile centres to *p*. The transformed point is defined as

$$\begin{array}{c} \sum_{i=0}^{3}w_{i}\left(M_{\theta_{i}}p+t_{i}\right), \end{array}$$

where *θ*_*i*_ and *t*_*i*_ are the transformation parameters associated with the tile centred at *c*_*i*_, and *w*_*i*_ is the weighting function, given by

$$\begin{array}{c} w_{i}=\frac{1/|p-c_{i}{|}^{2}}{\sum_{j=0}^{3}1/{\left|p-c_{j}\right|}^{2}}. \end{array}$$

This final transformation completes the process used to elastically register two slides. The next section describes how to combine the elastic registration process that has been described in the present section with the rigid registration algorithm described in the previous section to obtain an optimal representation of the original tissue.

#### Determination of registration order

The techniques outlined in the foregoing sections provide a means for registering slides and reducing distortion. What remains is to implement them to obtain a faithful representation of the original tissue. Initially slides were registered using the rigid registration, with each slide being registered to the previous slide in the *z*-stack. This produced a rough alignment which allowed the comparison of slides for distortion levels. The levels of distortion were then used to select the optimal reference slides for the elastic registration.

The elastic registration process registered each slide to a reference slide. The sectioning process created some distortion in each slide. For this reason the least distorted slides were designated most suitable to provide the reference for other slides. The process of selecting reference slides started with selecting a *global* reference slide. This global reference slide ultimately served as a reference slide for all other slides. *Local* reference slides were subsequently selected, working outwards from the global reference slide based on how well they matched the previous reference slide. Each reference slide was registered to the previous reference slide and as a result, all reference slides were effectively registered to the global reference slide. Finally, all non-reference slides were registered to their nearest reference slide to complete the registration process.

To determine a measure of how well two slides matched, a match score was calculated as follows:

$$\begin{array}{c} 1-\frac{\#\text{matched pixels}}{\text{average}\#\text{non}-empty\text{ pixel}}, \end{array}$$

where pixels are defined as matched if they are non-*empty* in both slides. This match score is a measure of the relative distortion of the two slides, with 0 being perfectly matched and 1 being completely mismatched.

The *global* reference slide was determined by identifying a slide with a large area of tissue present, which is quantified as the total number of non-*empty* pixels in the slide. This provides a reliable global reference as it gives a general cross-sectional shape of the tissue. To verify that this global reference slide was a robust representation of the tissue block, a substack of slides containing the largest average number of non-*empty* pixels was determined. All slides in this substack were then compared with each other to obtain an approximation of the level of distortion in each slide. The *global* reference slide was taken to be the slide in the substack with the lowest approximate distortion.

#### Verification of slide integrity

An accurate representation could be achieved using the registration algorithms detailed above, provided that the level of distortion was low. If the level of distortion of a slide was too high, however, it would not register to a reference slide. The cut-off point where the distortion becomes too high is not always evident from visual inspection of the slides prior to registration. Therefore, it is only after the registration is complete that a slide can be checked to determine if it has a sufficiently low level of distortion. This check was performed manually after each of the rigid and elastic registration steps had completed. If a slide tested was deemed to have failed to register accurately, it was discarded. If this registration failure occurred during the rigid slide registration, the next slide in the stack was registered to the previous slide to compensate for the inaccurate registration. If this registration failure occurred during the elastic registration and the slide was not a reference slide, no further registration was performed. If, however, the slide was a (local) reference slide, then all slides registered to this reference slide would be deformed by this inaccurate registration. For this reason, the elastic registration algorithm was run again from the last reference slide that had been accurately registered.

**Fig 11:**
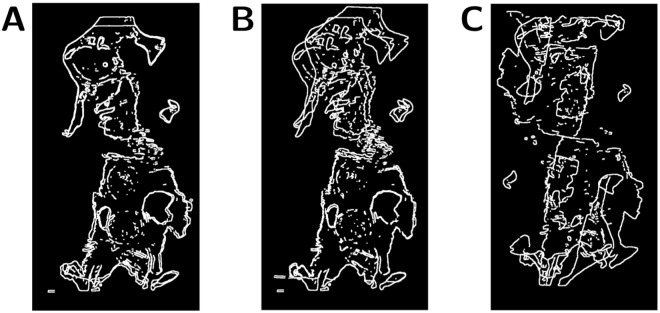
The possible outcomes of the rigid registration algorithm. The possible outcomes of the rigid registration algorithm. Each image is the sum of two slide edge images after rigid registration. **A**: An example of successful rigid registration. The two images are well-aligned, which means they pass the visual integrity test. This situation is the typical outcome of registration (88%). **B**: An example of partially successful rigid registration. In this example the slide being registered is distorted compared to the reference slide. This level of distortion exceeds the maximum elasticity allowed by the elastic registration algorithm, and therefore cannot be corrected in the elastic registration step, which means that this distorted slide is discarded. **C**: An example of failed rigid registration. In this case the slide being registered is discarded.

The manual testing of slide integrity after the rigid slide registration was performed by comparing the edge images. The edge image of a slide being tested was added to the edge image of its reference slide. This composite image was then examined by eye, and was deemed unacceptable if the edges were almost completely mismatched, or if a large level of distortion was observed. Examples of both of these cases are given in Fig 11.

**Fig 12:**
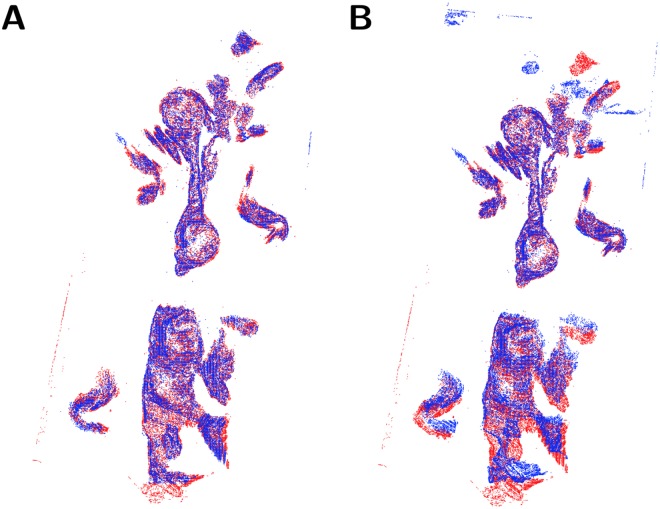
The possible outcomes of the elastic registration algorithm. The possible outcomes of the elastic registration algorithm. The images here compare the *planar* pixels of a reference slide in red with the *planar* pixels of a slide being registered in blue. **A**: An example of successful elastic registration. Here the slides show perfect alignment over the full length of the slide. This is the typical outcome of the elastic registration algorithm (75%). **B**: An example of failed elastic registration. Here the slides are aligned for part of the length of the slide, but have failed to correctly register on the lower half. This occurs when the slide being registered is too distorted for the elastic registration algorithm to fix. Accordingly, the slide being registered here is discarded.

The testing of slide integrity after the elastic slide registration was performed by comparing *planar* pixels of slides, as illustrated in Fig 12. This visualisation was used to compare elastically registered slides instead of edge images because a slide might fail to register accurately on the interior of the tissue, while remaining accurately registered at the edges of the tissue. Each slide was compared to the reference slide to which it was registered. A slide was deemed inaccurately registered if its relative distortion compared to the reference slide was clearly visible from the overlaid *planar* pixels. An example of a slide deemed inaccurately registered and an example of a similar slide deemed accurately registered are given in Fig 12.

### Phase IV: Generating uniformly spaced representative slides

The next step in the semi-automated processing pipeline is to create a uniformly spaced stack of slides from the registered slides. This step prepares the data for the next phase, which is the crucial step of merging the data into a three-dimensional representation.

**Fig 13:**
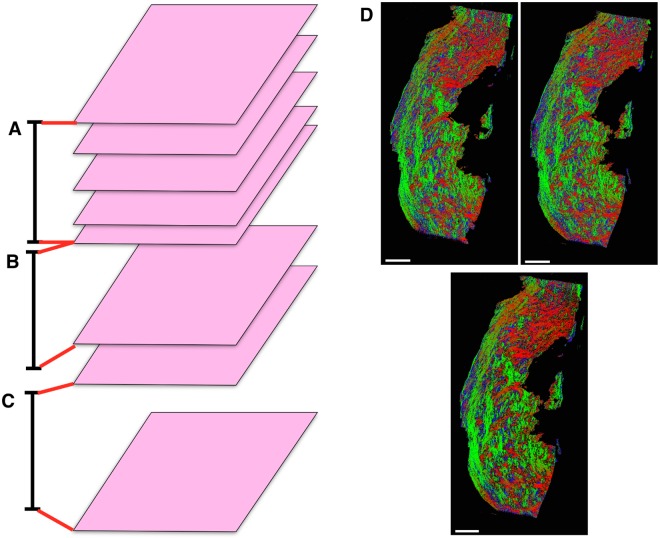
Generating a stack of representative slides. **A**–**C**: Selecting slides to combine to generate representative slides. The stack of irregularly-spaced slides is divided into ranges (black bars) containing substacks. Red bars indicate the range of slides used to generate the representative slides. When there is more than one slide present in a range (**A**), the substack contained in the range is used to generate the representative slide. If only one slide is present in the range (**B**), the nearest slide outside of the range is added to the substack. If no slides are present in the range (**C**) then the nearest slides either side of the range are taken as the substack. **D**: Representative slide (bottom) generated from two registered slides (top), where red indicates *planar* pixels with left-right direction in the image plane, green indicates *planar* pixels with up-down direction, and blue indicates *vertical* pixels. Scale bars represent 5 mm.

Slides were discarded during tissue preparation and registration, which means that the remaining slides form an irregularly-spaced substack of the full set of slides taken from the tissue, as shown in Fig 13A–13C. In addition, one issue to contend with is that the minimum distance between slides (5 *μ*m) is an order of magnitude smaller than the length of the pixels (∼50 *μ*m) used to generate the coarse-grained two-dimensional representation of bundle direction in **Phase II**, making the *z*-resolution up to 10 times higher than the effective slide resolution. Another issue is the reliability of the bundle directions in individual slides. The elastic registration (**Phase III**) corrected large distortions of the tissue, but distortion in the order of 50 *μ*m would be too small to be corrected in this manner. Small distortions of this kind would change the bundle direction of the regional representation at the pixel level, which reduces the reliability of the inferred direction in each pixel. This distortion varies between slides, and therefore combining directional data from multiple slides can be used to improve the reliability of the inferred bundle direction. For these reasons, the slides were condensed into a new stack of slides, with uniform separation equal to the length of the pixels generated in **Phase II**, as follows. The stack of slides was first divided into a set of substacks of uniform thickness, as illustrated in Fig 13. Each of these substacks was then mapped onto a representative slide in a new stack. The substack size was chosen so that the total thickness of a substack was equal to the length of a pixel. Thus the range of a substack, with representative slide at position *z*, was given by

$$\begin{array}{c} zl_{r}/l_{s}\leq s<\left(z+1\right)l_{r}/l_{s},s\in S \end{array}$$

where *S* is the set of all slide numbers in the original stack, *l*_*r*_ is the length of a pixel in *μ*m, and *l*_*s*_ is the thickness of the sections in *μ*m.

Each representative slide was generated by combining nuclear data from each slide in the substack, yielding a stack of evenly-spaced slides that form a three-dimensional volume. To reflect the three-dimensional nature of the stack, the discrete units of the representative slides will be referred to as voxels, which form the same two-dimensional layout as the pixels generated in **Phase II**. These voxels were assigned categories and direction in the same manner as the pixels in **Phase II**, based on the nuclei present in the volume represented by the given voxel. The average cross-sectional nuclear count was also assigned to each voxel as opposed to the total nuclear count assigned in **Phase II**, given by the total number of nuclei present divided by the number of slides in the substack.

The procedure as outlined above could result in a substack with fewer than two slides. In the case of a substack containing one slide, the nearest slide outside the given range is added to this substack (Fig 13B). In the case of an empty substack, the nearest slides present after registration on either side of the given range are used to generate the representative slide (Fig 13C). Extending the range in each of these cases allows the generation of reliable representative slides for any sparsely populated areas of the stack.

The resulting representative slide generated from two individual slides is shown in Fig 13D. The representative slide shows higher contrast in colours, which represent directions, while maintaining the overall structure. Higher contrast represents a more uniform local direction within fibrous structures, which indicates that these directions are more reliable representations of the original fibrous structure than the input slides.

### Phase V: Determining the three-dimensional direction field

The nuclear data obtained previously provided orientations of nuclei within the plane. The shape of the nuclei allowed a broad categorisation of the *z*-direction, but did not provide a reliable direction, due to the variability in shape and size of the individual nuclei. A fundamental difficulty is that the elliptical footprint left by a nucleus in the cut-plane (the *xy*-plane) is consistent with *two* headless direction vectors, which are related by a mirror reflection in the perpendicular plane through the minor axis of the ellipse (the headless vectors coincide when the footprint is either maximally or minimally eccentric, since on the former case the cell lies parallel to the cut-plane, and in the latter it is perpendicular to this plane). A further difficulty is that, while in theory it should be possible to identify these two vectors for a given nuclear shape, small variations in the shape can cause large changes in the direction; in particular, the uncertainty introduced by the resolution of the images is sufficient to cause an uncertainty of up to 45°. To resolve this indeterminacy, a consistency maximisation technique is used; in particular, the local bundle structure around the voxel was utilised.

At this point in the processing pipeline, the bundle structure has not been explicitly determined, but it is possible to infer some aspects of this structure from the two-dimensional data. The aim is to identify boundaries where the two-dimensional direction is discontinuous. Three-dimensional directions are assigned to points along these boundaries, using the boundary as a guide, and the remaining directions are assigned by working away from these boundaries using the previously assigned directions as a guide. This procedure was used to determine the three-dimensional direction for all *planar* voxels, which have an assigned two-dimensional direction. These three-dimensional directions were then used as a guide to determine the three-dimensional direction of the *vertical* voxels.

The process for determining the three-dimensional directions is as follows. The *planar* voxels were sorted in order of distance from the inferred boundaries and assigned three-dimensional direction in sequence, then the *vertical* voxels were similarly sorted and assigned directions. For each voxel in the sequence, the distance to the inferred boundaries along multiple directions was measured, and the optimal direction was taken to be a weighted average of these directions, where the weight was dependent on this distance. These steps require the definition of the inferred boundary and distance, which are given next.

Inference of boundaries requires a comparison of vectors. During the course of the following algorithm voxels are assigned direction vectors, which are in turn used to improve on the boundary detection for voxels later in the sequence. This means that the comparison of voxels must account for a mix of voxels assigned three-dimensional vectors, voxels assigned two-dimensional vectors (*planar*), voxels representing tissue with no assigned direction (*vertical*), and *empty* voxels. This comparison is aided by the use of spherical polar coordinates (*θ*, *ϕ*) to represent the unit direction vectors *v*:

$$\begin{array}{c} v=(\cos\theta \cos\phi ,\sin\theta \cos\phi ,\sin\phi ). \end{array}$$

This formulation of three-dimensional vectors can be compared to planar directions by only considering the *θ* term, while the *ϕ* term can be used to measure how close *v* is to the vertical direction. Consider a voxel *p* with an assigned direction given by (*θ*, *ϕ*). A voxel *q* adjacent to *p* is said to be in the same bundle as *p* if it is non-*empty* and one of the following hold:

1. *q* has an assigned vector given by (*θ*_1_, *ϕ*_1_) with |*θ*_1_−*θ*| < 30° and |*ϕ*_1_−*ϕ*| < 30°
2. *q* is *planar* with angle *θ*_1_ and |*θ*_1_−*θ*| < 30°
3. *q* is *vertical* and *ϕ* > 30°.

These criteria enable the comparison of a voxel with an assigned three-dimensional direction to any other type of voxel, which is sufficient to define the distance from a point to a bundle edge along a given direction.

**Fig 14:**
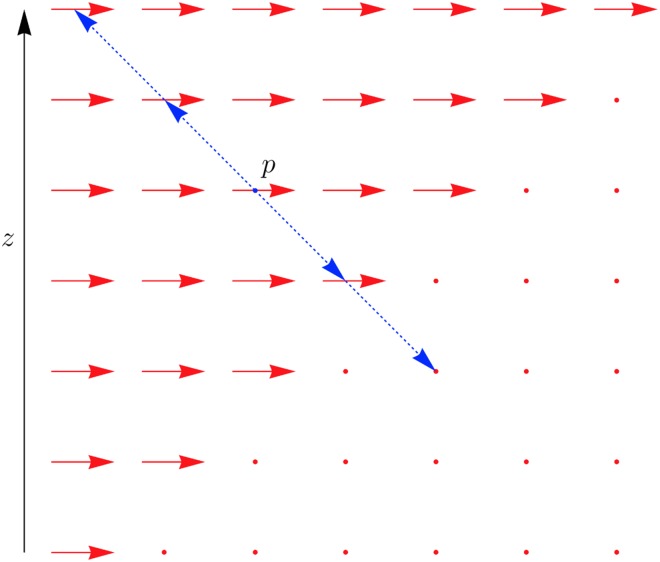
Measuring the distance to a inferred boundary along a direction vector. Red arrows represent the planar directions at each point, red dots represent a planar direction through the plane being viewed. The direction vector *v* in this example is taken to be −45° to the slide plane. The distance *L*(*p*, *v*) is measured as the number of discrete steps, shown here with blue arrowheads, along *v* between *p* and a stopping point where either a dramatic change in direction occurs, as shown here, or an empty voxel is reached. This stopping point is considered to be the edge of the bundle containing *p*.

Each step in the algorithm described below requires a means to compare potential direction vectors for a voxel in order to obtain the optimal direction vector. The measure used to compare vectors is the distance along a direction vector from the voxel to the nearest inferred boundary, as illustrated in Fig 14. This distance is bounded above by a constant value, in order to ensure that it is measuring local bundle structure. Consider a voxel at point *p* and a direction vector *v*. The line *l* passing through *p* with direction *v* is defined by

$$\begin{array}{c} l(\lambda )≔p+\lambda v \end{array}$$

for λ∈R. The line *l* can be discretised to a sequence of voxels at points ${\left\{p_{i}\right\}}_{i\in Z}$, defined by

$$\begin{array}{c} p_{i}≔p+\lambda_{i}v, \end{array}$$

where the sequence ${\left\{\lambda_{i}\right\}}_{i\in Z}$ is defined such that

$$\begin{array}{c} \lambda_{i}-\lambda_{i-1}=\min_{j}\frac{1}{v_{j}}, \end{array}$$

where *v*_*j*_ is the *j*th component of *v*, and

$$\begin{array}{c} \lambda_{0}=0. \end{array}$$

**Fig 15:**
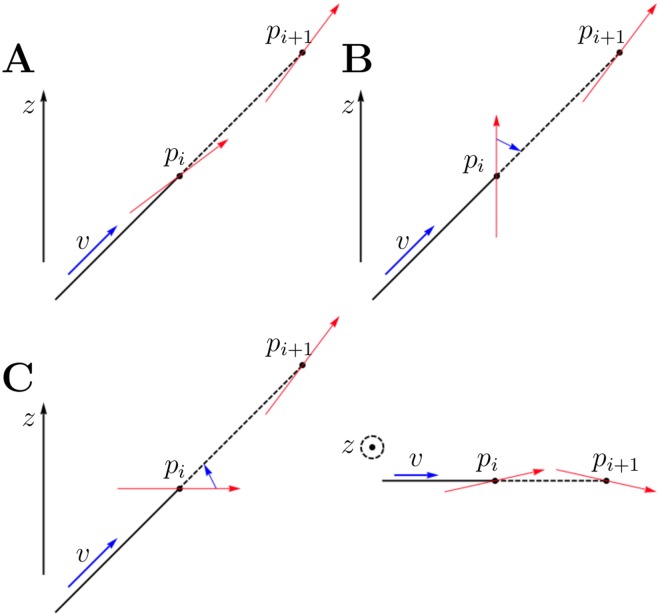
Temporary assignment of three-dimensional direction. **A**: The direction at *p*_*i*_ has already been determined in three dimensions, therefore no modifications are needed. **B**: The voxel at *p*_*i*_ has category *vertical*, meaning it has no predetermined direction, so the direction vector *v* is used. **C**: The voxel at *p*_*i*_ has category *planar*, meaning it already has a fixed planar direction, so this planar direction is rotated toward the *z*-axis by an angle *phi*, where (*θ*, *ϕ*) is the representation of *v* in spherical polar coordinates.

This sequence is constructed so that each of the points *p*_*i*_ is contained in a distinct voxel, and the voxel containing *p*_*i*_ is adjacent to the voxel containing *p*_*i*+1_. The distance along *l* to the nearest inferred boundary is determined by sequentially comparing the voxels containing {*p*_*i*_} outward from *p*_0_ = *p* along the direction vector *v*, using the above criteria. Suppose the voxel at *p*_*i*_ has been determined to be in the bundle containing *p*. For the sake of convenience assume that *i* ≥ 0. This voxel is compared with *p*_*i*+1_ to determine if *p*_*i*+1_ is also in the bundle containing *p*. The aim is to temporarily assign a vector *u* to *p*_*i*_ in order to apply the above criteria. If *p*_*i*_ has already been assigned a three-dimensional direction, then *u* is set to this direction. If *p*_*i*_ is *vertical* then *u* = *v*. If *p*_*i*_ is *planar*, *θ*_*p*_ is the planar angle assigned to *p*_*i*_, and (*θ*_*v*_, *ϕ*_*v*_) is the polar representation of *v*, then *u* is the unit vector represented by the polar coordinates (*θ*_*p*_, *ϕ*_*v*_). The voxel *p*_*i*_ with the assigned vector *u* can now be compared with *p*_*i*+1_ to determine if they are in the same bundle. These various cases are illustrated in Fig 15. This sequential comparison is performed in both directions simultaneously, and stops when one of *p*_±*i*_ is no longer contained in the same bundle. The stopping index *i* is denoted by *L*(*p*, *v*), and represents the length of the line from *p* to the nearest inferred boundary in the direction *v*. This measure is used below to compare direction vectors: if, for a voxel at *p*, the direction vectors *v*_0_ and *v*_1_ are such that

$$\begin{array}{c} L\left(p,v_{0}\right)>L\left(p,v_{1}\right), \end{array}$$

then the line extending from *p* in the direction *v*_0_ is longer than in the direction *v*_1_, and therefore *v*_0_ is considered more likely than *v*_1_ to represent the true direction at *p*.

The process for determining the three-dimensional direction of a voxel differed depending on whether the voxel was categorised as *planar* or *vertical*, as is evident in the methods detailed below. Since the *planar* voxels already have a planar direction assigned, all *planar* voxels were assigned *z*-directions prior to assigning directions to the *vertical* voxels. In both of these sets the voxels were assigned in a specific order, as described below. This ordering enabled the voxels to which a direction can be assigned with a higher degree of confidence to be assigned first, and this information can be used to reduce the uncertainty in direction in the remaining voxels.

The following measure was used to determine the ordering of the *planar* voxels. For a *planar* voxel at *p*, with planar direction defined by *θ*, the function *s*(*p*) is given by

$$\begin{array}{c} s\left(p\right)=|L\left(p,v_{+}\right)-L\left(p,v_{-}\right)| \end{array}$$

Where *v*_+_ and *v*_−_ are defined by

$$\begin{array}{c} v_{+}=\left(\cos\theta \cos60,\sin\theta \cos60,\sin60\right) \end{array}$$

$$\begin{array}{c} v_{-}=\left(\cos\theta \cos60,\sin\theta \cos60,-\sin60\right). \end{array}$$

**Fig 16:**
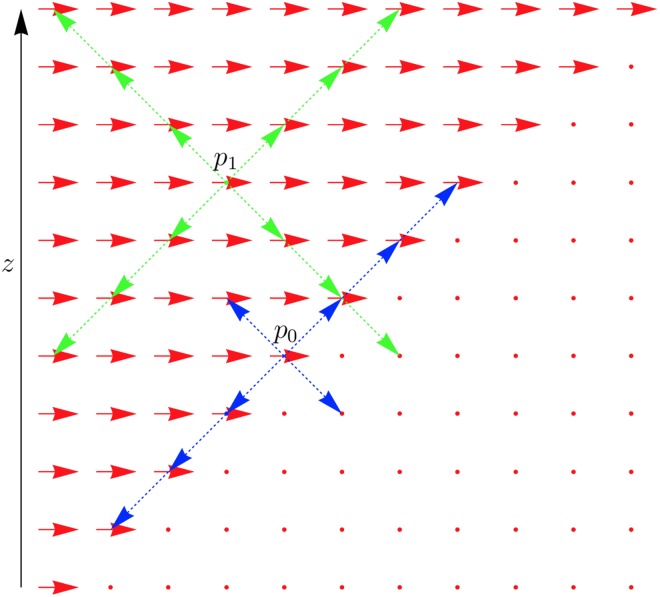
Comparison score of extreme *z*-angles. The point *p*_0_ is situated on the boundary between areas of dramatically differing directions and therefore the local structure will impose a tight restriction on the *z*-direction at *p*_0_. The point *p*_1_ is farther from this boundary, and therefore the *z*-direction is not clearly determined by the local planar directions. The value of *s* for each of these points reflects this effect by comparing the distance to such boundaries at the extreme angles shown.

This measure compares the lengths of the lines at 60° to the plane, with planar direction defined by the voxel. These two angles are used for this measure because, as detailed below, these values are the boundaries of the range of potential angles assigned to a *planar* voxel. Thus the score *s* compares the line lengths corresponding to the *z*-directions at either extreme. A high value of *s* corresponds to a higher chance of the *z*-angle being toward one of these extremes, which means that the voxel is more likely to have a well-defined *z*-direction based on the original planar directions, as illustrated in Fig 16. For this reason, the planar voxels are sorted into descending order of *s*-values.

The three-dimensional vector assigned to a *planar* voxel was bounded by the angles ±60°. This bound was selected to ensure that the final direction was constrained to angles below vertical, but still allowing the possibility that the true direction could be closer to vertical than in the slide plane.

For a *planar* voxel at point *p*, with planar direction defined by *θ*, the optimal *z*-angle $\hat{\phi }$ is defined such that

$$\begin{array}{c} L\left(p,\left(\cos\theta \cos\hat{\phi },\sin\theta \cos\hat{\phi },\sin\hat{\phi }\right)\right)=\max_{\phi \in [-60,60]}L\left(p,\left(\cos\theta \cos\phi ,\sin\theta \cos\phi ,\sin\phi \right)\right). \end{array}$$

**Fig 17:**
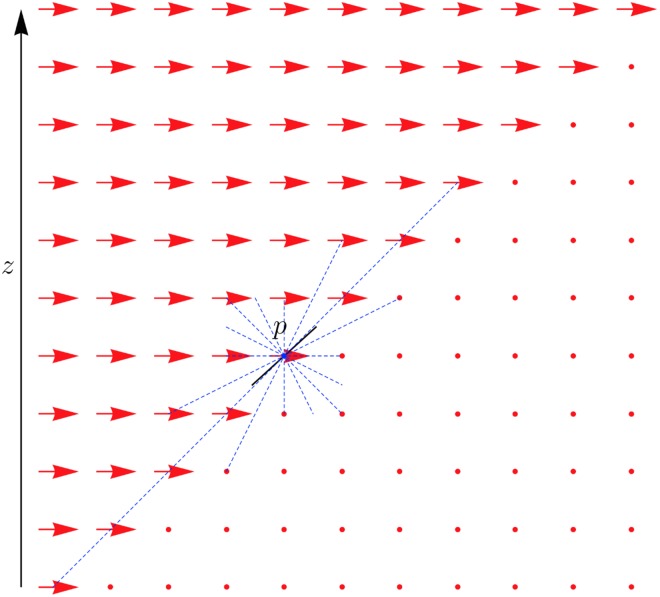
Assigning three-dimensional direction. The lines corresponding to a *z*-angle below 0 all have length below the length threshold, and consequently are not used in the calculation of the average angle. The weighted average of the directions with sufficiently large lengths is shown here as a black line. This direction is a good approximation to the actual direction along the edge of the bundle.

To approximate $\hat{\phi }$, an evenly spaced set of angles ${\left\{\phi_{i}\right\}}_{i=0}^{n-1}$, ranging from *ϕ*_0_ = −60° to *ϕ*_*n*−1_ = 60° with separation 15° was used. For each angle *ϕ*_*i*_ the length

$$\begin{array}{c} L_{i}≔L\left(p,\left(\cos\theta \cos\phi_{i},\sin\theta \cos\phi_{i},\sin\phi_{i}\right)\right) \end{array}$$

was calculated. The weighted average

$$\begin{array}{c} \bar{\phi }≔\sum_{i\in S}\frac{L_{i}\phi_{i}}{\sum_{i\in S}L_{i}} \end{array}$$

was then taken to be the approximation of $\hat{\phi }$, where *S* is the set of all indices *i* for which *L*_*i*_ is larger than 2. This averaging is illustrated in Fig 17. This approximation, taken with the value *θ*, defines the three-dimensional direction at *p*, given by

$$\begin{array}{c} v\left(p\right)≔\left(\cos\theta \cos\bar{\phi },\sin\theta \cos\bar{\phi },\sin\bar{\phi }\right). \end{array}$$

Unlike *planar* voxels, *vertical* voxels have no predetermined planar direction. For this reason, both planar and *z*-directions must be found for the *vertical* voxels. To determine the three-dimensional direction of the *vertical* voxels, first the voxels are ordered in a manner analogous to that of the *planar* voxels described above. For a voxel at *p* the function *s*(*p*) is given by

$$\begin{array}{c} s\left(p\right)≔\max_{u,v\in V}\left|L\left(p,u\right)-L\left(p,v\right)\right|, \end{array}$$

where the set *V* is defined to be the set of vectors

$$\begin{array}{c} \{(\pm \cos45,0,\sin45),(0,\pm \cos45,\sin45)\}. \end{array}$$

This set is composed of the direction vectors which are at 45° to the (*x*, *y*)-plane and perpendicular to one of the *x*- or *y*-axes. This angle was selected because it allows the matching criterion to be met between *p* and *vertical* voxels, which have default angle 60, and with *planar* voxels which have been assigned angles away from the plane. As is the case for *planar* voxels, a high *s* confers more confidence that *p* has direction toward one of these extremes, and accordingly, the *vertical* voxels are sorted into descending order of *s*-values.

For a given *vertical* voxel at point *p*, the optimal planar angle $\hat{\theta }$ and *z*-angle $\hat{\phi }$ are defined such that

$$\begin{array}{c} L\left(p,\left(\cos\hat{\theta }\cos\hat{\phi },\sin\hat{\theta }\cos\hat{\phi },\sin\hat{\phi }\right)\right)=\max_{\theta \in [0,360)}\max_{\phi \in [30,90]}L\left(p,\left(\cos\theta \cos\phi ,\sin\theta \cos\phi ,\sin\phi \right)\right). \end{array}$$

To approximate $\hat{\theta }$ and $\hat{\phi }$, an evenly spaced set of planar angles ${\left\{\theta_{i}\right\}}_{i=0}^{n-1}$, ranging from *θ*_0_ = 0° to *θ*_*n*_ = 360° with separation 11.25° was used. For each planar angle *θ*_*i*_, the approximate optimal *z*-angle $\bar{\phi }\left(\theta_{i}\right)$ was calculated using the same technique as detailed above for *planar* voxels, with the exception that the range of *z*-angles was defined as [30, 90] as opposed to [−60,60]. The length

$$\begin{array}{c} L_{i}≔L(p,\left(\cos\theta_{i}\cos\bar{\phi }\left(\theta_{i}\right),\sin\theta_{i}\cos\bar{\phi }\left(\theta_{i}\right),\sin\bar{\phi }\left(\theta_{i}\right)\right) \end{array}$$

was calculated. The approximation $\bar{\theta }$ of $\hat{\theta }$ was defined to be the value *θ*_*i*_ with maximal associated length *L*_*i*_, since this angle gives the longest possible line within inferred fascicular (bundle) boundaries. This approximation, taken with the associated value of $\bar{\phi }$ defined the three-dimensional direction at *p*, given by

$$\begin{array}{c} v\left(p\right)≔\left(\cos\bar{\theta }\cos\bar{\phi },\sin\bar{\theta }\cos\bar{\phi },\sin\bar{\phi }\right). \end{array}$$

Using this technique the three-dimensional bundle directions were determined from the regional data.

### Phase VI: Weighting the direction vectors

The direction vectors found in the previous sections are weighted by two separate weighting functions, which represent the nuclear density and homogeneity. The weighting functions provide a measure of how well the tissue represented by a direction vector matches smooth muscle tissue. A low nuclear density would indicate that there is little tissue present in the given voxel, while a low level of heterogeneity in a voxel would indicate that the voxel is less likely to be contained in a smooth muscle fibre. These weighting functions were used to improve the accuracy of the reconstruction.

#### Nuclear density weighting

**Fig 18:**
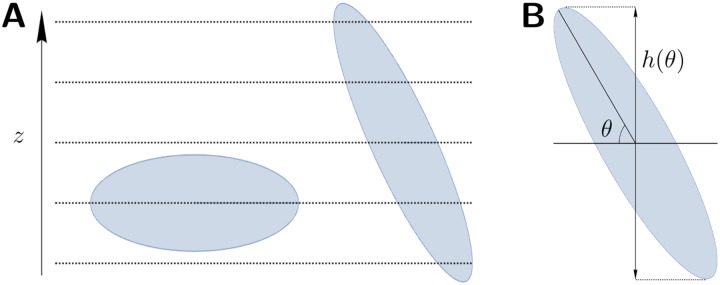
The effect of nuclear orientation on sample probability. **A**: Comparison of the sectioning of two nuclei at different angles to the plane. The nucleus lying within the plane is only sampled once, whereas the nucleus which is more vertical is sampled multiple times in sectioning. **B**: The height function *h* of angle *θ*. The value of *h*(*θ*) is the difference in height between the highest and lowest points on a nucleus at angle *θ*.

**Fig 19:**
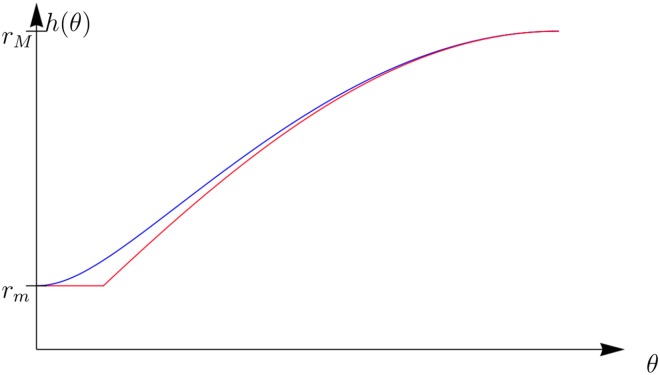
Approximation of weighting. A comparison of the value of *h*(*θ*) and a scaled sine curve which has been cut of at sin(*θ*) = *r*_*m*_. Here *r*_*M*_ is the length of a nucleus, and *r*_*m*_ is the width. This approximation is acceptable in this case in view of the high eccentricity of the nuclei.

Each voxel was weighted using the nucleus count present in the regional data as a measure of tissue density. This count represents the average number of nuclei observed in a slide through the given voxel. Because this value represents the number of nuclei observed within a plane, the direction of the nuclei in relation to this plane affect the number of nuclei observed, as illustrated in Fig 18A. This phenomenon is a common problem in stereology, as it creates a bias depending on the plane of slicing [23]; however, in the present setting the average orientation of nuclei has already been found in **Phase V**, which can be used to account for this bias as follows. A nucleus contained within a given voxel with angle from the plane *ϕ* is observed in any given plane with probability

$$\begin{array}{c} P\left(\text{observed}\right)=\frac{h(\phi )}{l} \end{array}$$

where *l* is the length of the voxel, and *h*(*ϕ*) is the length of the vertical projection of the nucleus as illustrated in Fig 18B. For ease of calculation, the function *h* was approximated by

$$\begin{array}{c} h\left(\phi \right)\approx \max\left\{r_{M}\sin\phi ,r_{m}\right\} \end{array}$$

where *r*_*M*_ is the length of the major axis and *r*_*m*_ is the length of the minor axis of a nucleus. A comparison of this approximation with the actual value of *h*(*ϕ*) is shown in Fig 19. Using this probability, the average number of nuclei observed in a plane *N*_*p*_ can be expressed as a function of the total number of nuclei in the voxel *N*:

$$\begin{array}{c} N_{p}≔E\left(\text{number}\;\text{of}\;\text{observed}\right)=\sum_{i}P\left(\text{nucleus}\;i\;\text{observed}\right)=\sum_{i}\frac{h(\phi_{i})}{l}=\frac{h(\phi )}{l}N, \end{array}$$

where E(·) denotes expectation, {*ϕ*_*i*_} are the vertical angles of the nuclei and *ϕ* is the average vertical angle of the nuclei. The value of *ϕ* is given by the three-dimensional direction vector *d* assigned to the voxel:

$$\begin{array}{c} \sin\phi =d_{z}. \end{array}$$

Thus the number of nuclei within a given voxel is approximated by

$$\begin{array}{c} N\approx \min\left\{\frac{N_{p}l}{r_{M}d_{z}},\frac{N_{p}l}{r_{m}}\right\}. \end{array}$$

The values for *r*_*M*_/*l* and *r*_*m*_/*l* are given in Table 3. The total number of nuclei within a voxel can therefore be estimated on the basis of the average number of nuclei in the plane and the *z*-component of the direction.

**Table 3:** Parameter values for the density weighting function.

Parameter	Value
*r*_*M*_/*l*	0.3
*r*_*m*_/*l*	0.1
*N*_0_	10
*N*_1_	160

These values specifically relate to the high-resolution capture of the tissue. While these values are technically dependent on resolution, a scaling of the length values corresponds to a reciprocal scaling of the nuclear count values, which compensates for this effect.

The weight of a voxel with inferred nuclear count *N* is defined as

$$w_{d}\left(N\right)≔\{\begin{array}{ll} N/N_{0} & \text{if }N\leq N_{0}\\ 1 & \text{if }N_{0}<N<N_{1}\\ 0 & \text{if }N_{1}<N \end{array}$$

where *N*_0_ is selected such that if *N* ≥ *N*_0_ then the voxel is filled with conducting tissue. The upper bound *N*_1_ removes voxels which contain more nuclei than could feasibly be present in smooth muscle tissue. The parameter values for the weightings of low- and high-resolution reconstructions are given in Table 3.

#### Nuclear homogeneity weighting

**Fig 20:**
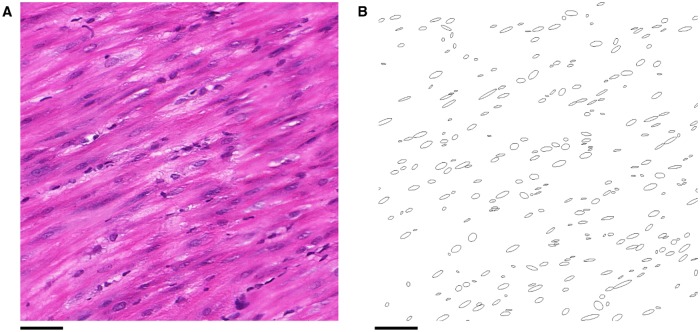
Isotropy within a region. Within a sufficiently small volume the nuclei in smooth muscle tissue have similar orientation. **A**: A histological image of smooth muscle tissue. **B**: The orientation of the nuclei within the slide plane from **A**. Scale bars represent 50 *μ*m.

The direction of nuclei within smooth muscle tissue is relatively homogeneous in small volumes, as illustrated in Fig 20. Within a voxel, which has width ∼50 *μ*m, it is reasonable to assume that the orientation of nuclei within smooth muscle tissue is approximately constant. Therefore, a low level of homogeneity within a voxel would indicate that either the voxel does not contain smooth muscle, or that the voxel lies on the boundary of multiple bundles. The method used to measure homogeneity is similar to the method for smoothing directions in two-dimensional segmentation, which is detailed below. For a given voxel, let *S* be the set of angles of orientation of the nuclei contained in the voxel and *ϕ* be the *z*-angle assigned to the voxel. Define the vector-valued function *v*(*θ*, *ϕ*) by

$$\begin{array}{c} v(\theta ,\phi )≔(\sin\phi \cos(2\theta ),\sin\phi \sin(2\theta ),\cos\phi ). \end{array}$$

The measure of homogeneity for the given voxel is defined to be

$$\begin{array}{c} w_{h}\left(S,\phi \right)≔\frac{{\left|\sum_{\theta \in S}v\left(\theta ,\phi \right)\right|}^{2}}{\left|S\right|}. \end{array}$$

While three-dimensional vectors are used in this measure, it is not necessary to double the angles in the *z*-direction, because the value of *z* is constant within the voxel. The product of this homogeneity function with the density weighting function described in the previous section gives the full weighting of each voxel in the tissue.

### Phase VII: Segmentation of the fibrous structure in two and three dimensions

**Fig 21:**
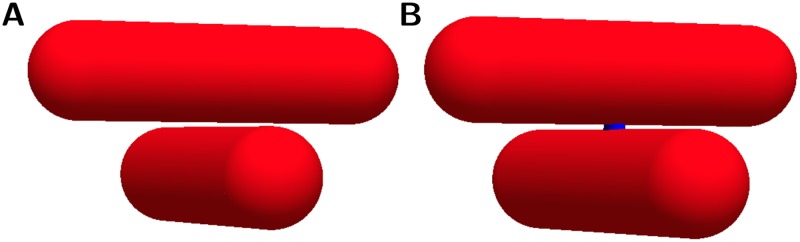
Different forms of adjacent bundles. **A**: The two tubes shown here represent fibrous structures. They are close together, but not communicating. At resolutions comparable to the size of the gap, these structures may appear to touch. This is a problem when the aim is to resolve individual structures and to determine conductivity in the tissue. **B**: A bridge structure (blue) has been added. In this situation the bundles are connected by a filamentous structure and therefore there should be a connection in the digital representation. Even though the two situations are similar, they have substantially different electroconductive properties, and therefore need to be differentiated.

The fibrous structure of the *in silico* tissue reconstruction was determined by segmenting the tissue using an edge detection algorithm. These edges must completely encase a fibrous structure, which enables the distinction of two adjacent bundles that are not connected by a bridge, as illustrated in Fig 21. Edge detectors which rely on a local differencing operator, such as the Canny edge detector, are not suitable for this kind of differentiation, since they tend to form incomplete boundaries [24]. To overcome this problem, a variant of watershed segmentation is used, as described in the following sections. Watershed algorithms have the general property that any boundary is continuous [25], and will therefore enable the distinction between bundles to be absolute. Segmentation of fibrous structure was performed in three dimensions to enable better representation of the fibrous structure in the *in silico* reconstruction.

Segmentation in two dimensions was performed to measure bundle widths, which is used for comparison between registered and unregistered slides. The techniques used for segmentation in two dimensions differ from those used in three dimensions, since the nature of the direction vectors is fundamentally different between the two cases: in two dimensions the directional data take the form of two-dimensional planar angles and vertical points, whereas in three dimensions all directions are represented by a three-dimensional vector. The general formulation of the algorithm in both cases is the same, however. Initially, a grey image or volume is generated from the directional data using a form of Gaussian smoothing. This grey image is used to perform the watershed segmentation, which generates the bundle edges.

#### Watershed segmentation with merging

A watershed algorithm uses the grey values of an image to determine boundaries between local gradient minima [25]. The watershed algorithm used in the following determines these separating lines as follows. Each point *p* in the image is sorted in order of increasing gradient values, to give the list ${\left\{p_{i}\right\}}_{i=0}^{n-1}$. This list is subsequently separated into sets of points, which shall be referred to as pools. This separation is performed sequentially from *p*_0_ using the following steps. For each *p*_*i*_, consider all points *p*_*j*_ adjacent to *p*_*i*_. If *i* < *j* for all *j*, then none of the points *p*_*j*_ have been placed into a pool, and so *p*_*i*_ is placed into a new pool. If all points *p*_*j*_ with *j* < *i* are contained in the same pool, then *p*_*i*_ is added to that pool. Otherwise, *p*_*i*_ is on the boundary between two or more pools, and therefore is not placed into any pools. Such unassigned points form the lines separating the features, and the set of all these points is referred to as the watershed.

The following sections outline a method of generating greyscale images that represent local anisotropy in two and three dimensions. Areas of high anisotropy are more likely to correspond to areas within fibrous structures, while areas of low anisotropy are more likely to correspond to points on the boundary between fibrous structures. For this reason, the watershed segmentation algorithm sorts points in order of decreasing local anisotropy, rather than increasing gradient. As will become apparent in the following sections, two local maxima in the greyscale anisotropy image do not necessarily correspond to two separate features. If the above watershed algorithm were performed on such an image, this would lead to erroneous boundaries forming, a phenomenon known as over-segmentation [26]. For this reason, an additional step of merging pools was employed. For two local maxima *p* and *q*, a comparison function *f*(*p*, *q*) is used which represents how well the local directions about *p* and *q* are matched. The specific formulation of *f* is dependent on the dimensionality of the problem, and shall therefore be described separately in the following sections. This function can be used to modify the watershed algorithm in the following manner. Consider a point *p*_*i*_ which has been found to lie on the watershed boundary of the two pools, and let *p* and *q* be the local maxima in each of these pools. The point *p*_*i*_ is considered a watershed point if

$$\begin{array}{c} f\left(p,q\right)<f_{\text{min}}, \end{array}$$

where *f*_min_ is a constant threshold. This means that if the direction fields about *p* and *q* are sufficiently well-matched, no watershed line is drawn between them. The modification can be extended to the case where *p*_*i*_ lies on the boundary of more than two pools. In this case, all such pairs of local maxima *p* and *q* are compared, and *p*_*i*_ is deemed a watershed point if any of the pairs satisfy the above inequality.

This watershed-and-merging algorithm was employed to segment directional images in two and three dimensions. The following sections describe in detail how the grey image or volume is generated in order to perform the watershed algorithm and how the comparison function *f* is formulated.

### Quantifying vector images

Segmentation in both two and three dimensions using the above algorithm requires the quantification of vector images and a comparison function. Both of these enumerations ultimately require a measurement of similarity between vectors, either within a local area for generating greyscale images, or between two areas for the comparison function. This similarity measurement must also account for the fact that the vectors present in the images represent both forward and backward directions; for this reason, the square of the scalar product is used.

Let *p* be a point in some weighted vector image *V*_*w*_, and *G* = {*g*_*j*_}_*j* ∈ *N*(*p*)_ be a Gaussian kernel applied in the neighbourhood *N*(*p*) about *p*. The image *I*_*w*_ representing *V*_*w*_ is given by

$$\begin{array}{c} I_{w}\left(p\right)≔\sum_{j\in N(p),k\in N(p)}g_{j}g_{k}w_{j}w_{k}\left(2{\left(v_{j}\cdot v_{k}\right)}^{2}-1\right), \end{array}$$ (1)

where *v*_*j*_ is the vector in *V*_*w*_ corresponding to the Gaussian entry *g*_*j*_ with weight *w*_*j*_, and similarly for *v*_*k*_ and *w*_*k*_. Entries in this image have values in the range [0, 1], and represent the weighted average of similarity of vectors in *N*(*p*), where the weighting applied is both the inherent weighting of *V*_*w*_ and the Gaussian weights. Computing the terms of these sums for each point in *I*_*w*_ is time-consuming; the following two sections present techniques to reduce the computational workload in 2 and 3 dimensions.

The merging step described in the previous section also requires a comparison function for two points in the image. For points *p* and *q* in *V*_*w*_ with respective neighbourhoods {*p*_*j*_}_*j* ∈ *N*(*p*)_ and {*q*_*k*_}_*k* ∈ *N*(*q*)_, the comparison function *f* is defined:

$$\begin{array}{c} f\left(p,q\right)≔\sum_{j\in N(p),k\in N(q)}\frac{g_{k}g_{j}w\left(p_{k}\right)w\left(q_{k}\right)}{\sum_{nm}g_{n}g_{m}w\left(p_{n}\right)w\left(q_{m}\right)}{\left(v_{j}\cdot u_{k}\right)}^{2}, \end{array}$$ (2)

where *w*(*) is the weighting function, g_*_ are entries of the Gaussian matrix as above, *v*_*j*_ is the vector at the point *p*_*j*_, and *u*_*k*_ is the vector at the point *q*_*k*_. This function compares the vectors in the neighbourhood of *p* to those in the neighbourhood of *q*, where a value of 1 corresponds to homogeneous neighbourhoods of the same direction, while a value of 0 corresponds to homogeneous neighbourhoods with perpendicular directions. Once again, computing these sums for multiple pairs of points is time-consuming, and a more efficient method for computing these sums is presented in the following sections.

#### Segmentation in two dimensions

The regional data for each slide consist of *planar*, *vertical*, and *empty* pixels. These categorisations represent an approximation of the fibre direction perpendicular to the plane, with *planar* representing a fibre running parallel to the plane and *vertical* representing a fibre running perpendicular to the plane. Accordingly, the bundle edges were determined separately for each of the two categories. The parameter values for the following procedure are given in Table 4.

**Table 4:** Parameter values for two-dimensional bundle segmentation.

Parameter	Value
Gaussian variance	1.0
Vertical grey level threshold	10.0
*f*_min_	0.54
Planar grey level threshold	0.01

The same Gaussian operator was applied in both *planar* and *vertical* segmentation processes.

**Fig 22:**
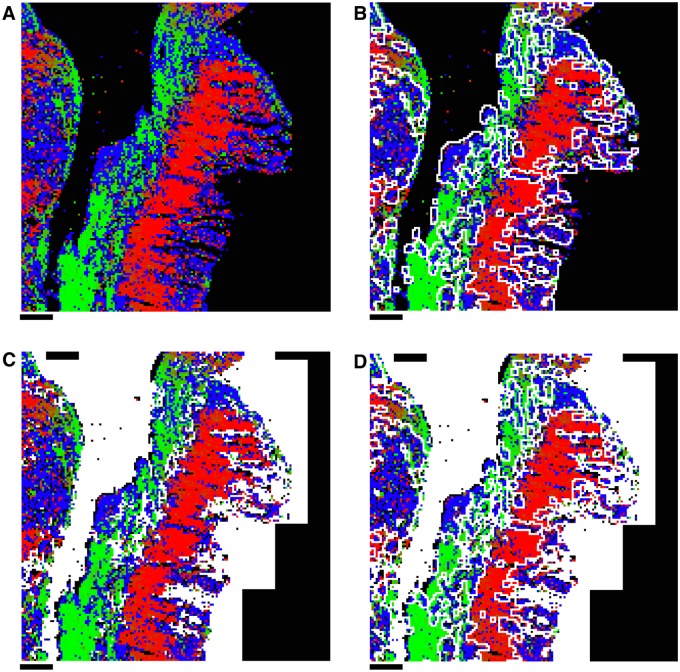
Two-dimensional edge detection. An example of the edges generated from the two separate edge detection algorithms. **A**: A colour representation of the regional directions, where blue indicates vertical pixels, while red and green indicate planar pixels. The red and green levels indicate direction; the green level is the *y*-component of the direction and the red level is the *x*-component. **B**: The edges obtained from the vertical pixels. Here the edges, shown in white, have been drawn around areas of blue with sufficiently high nuclear counts. **C**: The edges obtained from the planar pixels. Here a thresholding algorithm has turned all points in the grey image below the threshold white, and edges are formed between areas of mismatched directions. **D**: The combined edges from **B** and **C**, yielding the final segmentation boundaries. Scale bars represent 1 mm.

Edge detection for pixels labelled *vertical* is simplified by the fact that these pixels lack planar direction: the absence of a vector-valued attribute means that these pixels of each slide can be considered to represent a grey image, with values equal to the assigned nuclear count. In order to reduce the presence of noise, a Gaussian smoothing operator was applied to the image. To determine the edges of these vertical structures, a lower threshold on the grey values of the image is set. A point is deemed to be an edge point if its grey value is below this threshold and adjacent to a point that has a grey value above the threshold. The result of this procedure is shown in Fig 22B. Parameters for this procedure are listed in Table 4. The same Gaussian operator was applied in both *planar* and *vertical* segmentation processes.

**Fig 23:**
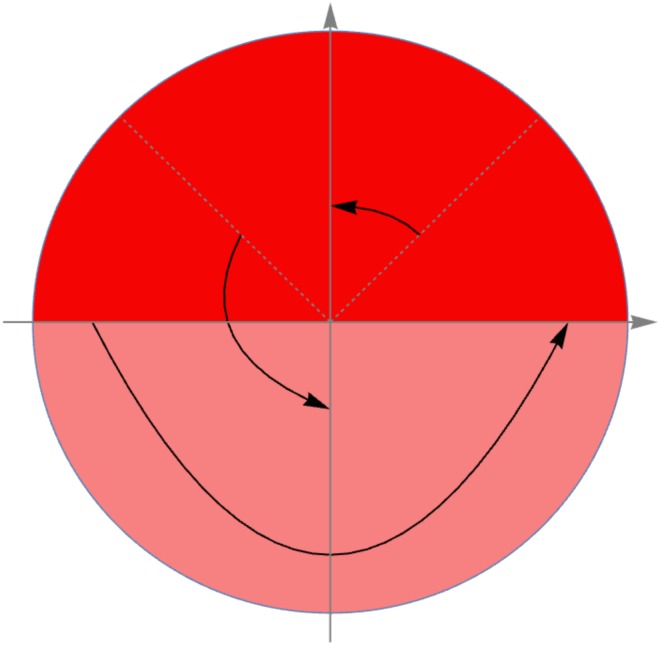
Double angles in two dimensions. Each vector is transformed by a rotation about the origin, doubling the polar angle. The dark red indicates the original position of the vectors, while the lighter red indicates the extra range included with transformation.

For pixels labelled *planar*, the aim is to generate the image defined in Eq (1). Consider the unit vector *v* in the vector image *V*, which makes an angle *θ* with the *x*-axis, and define the vector *v* by

$$\begin{array}{c} v^{\prime }=\left(\cos\left(2\theta \right),\sin\left(2\theta \right)\right), \end{array}$$

as illustrated in Fig 23. Let the vector image *V*′ be given by the above double-angle formulation. For a given point *p*, the vector obtained by the convolution of a Gaussian kernel *G* with *V*′ is given by

$$\begin{array}{c} \left(G*V^{\prime }\right)\left(p\right)=\sum_{k}g_{k}v_{k}^{\prime }, \end{array}$$

where *g*_*k*_ is the value of the Gaussian kernel corresponding to point *p*_*k*_, $v_{k}^{\prime }$ is the vector value of *p*_*k*_ in *V*′, and the set {*p*_*k*_} is the set of points in the window about *p*. Taking the norm squared of this vector yields

$$\begin{array}{c} \begin{array}{cc} |\left(G*V^{\prime }\right)\left(p\right){|}^{2} & ={\left|\sum_{k}g_{k}v_{k}^{\prime }\right|}^{2}\\ & =\left(\sum_{j}g_{j}v_{j}^{\prime }\right)\cdot \left(\sum_{k}g_{k}v_{k}^{\prime }\right)\\ & =\sum_{jk}g_{j}g_{k}\left(v_{j}^{\prime }\cdot v_{k}^{\prime }\right) \end{array}. \end{array}$$

The scalar product $v_{j}^{\prime }\cdot v_{k}^{\prime }$ that occurs in the summand of this last expression is given by

$$\begin{array}{c} v_{j}^{\prime }\cdot v_{k}^{\prime }=\cos\left(2\theta_{j}-2\theta_{k}\right), \end{array}$$

where *θ*_*j*_ and *θ*_*k*_ are the angles corresponding to the original vectors at *p*_*j*_ and *p*_*k*_, respectively. This can be expressed in terms of these original vectors *v*_*j*_ and *v*_*k*_:

$$\begin{array}{c} v_{j}^{\prime }\cdot v_{k}^{\prime }=2\cos^{2}\left(\theta_{j}-\theta_{k}\right)-1=2{\left(v_{j}\cdot v_{k}\right)}^{2}-1. \end{array}$$

Thus, the image *I* defined by

$$\begin{array}{c} I\left(p\right)≔|\left(G*V^{\prime }\right)\left(p\right){|}^{2} \end{array}$$

has entries

$$\begin{array}{c} I\left(p\right)=\sum_{jk}g_{j}g_{k}\left(2{\left(v_{j}\cdot v_{k}\right)}^{2}-1\right). \end{array}$$

**Fig 24:**
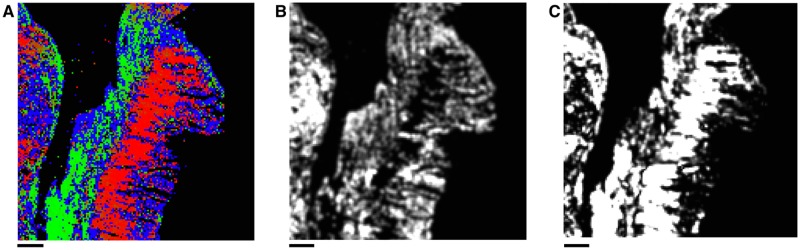
Scalar representation of two-dimensional vectors. **A**: A representation of the directions vectors, where red and green indicate the direction of planar vectors, and blue indicates vertical pixels. **B**: The grey image generated from smoothing the nuclear counts present in the vertical pixels. **C**: The grey image generated from the planar vectors represented in **A**. Scale bars represent 1 mm.

This can be converted to the form of Eq (1) by including weights, which are taken to be the nuclear counts assigned to the pixels, giving

$$\begin{array}{c} I_{w}\left(p\right)=\left|\left(G*V_{w}^{\prime }\right)\left(p\right)\right|=\left|\sum_{k}g_{k}w_{k}v_{k}^{\prime }\right|=\sum_{jk}g_{j}g_{k}w_{j}w_{k}\left(2{\left(v_{j}\cdot v_{k}\right)}^{2}-1\right), \end{array}$$

where

$$\begin{array}{c} V_{w}\left(p\right)=W\left(p\right)V\left(p\right) \end{array}$$

for the weighting image *W* with entries equal to the nuclear counts. An illustration of this weighted image is shown in Fig 24C. The image is a scalar-valued image that has been subjected to Gaussian smoothing. Prior to edge detection, a low level threshold is applied to the grey image to remove points with high levels of heterogeneity. This threshold ensures that any points which are manifestly not an interior part of any bundle are removed.

The second element of the watershed algorithm is computing the merging function *f* given in Eq (2). As is demonstrated below, the function *f* can be written

$$\begin{array}{c} f\left(p,q\right)=\frac{|\left(G*V_{w}^{\prime }\right)\left(p\right)+\left(G*V_{w}^{\prime }\right)\left(q\right){|}^{2}-I_{w}\left(p\right)-I_{w}\left(q\right)}{4(G*W)(p)(G*W)(q)}+\frac{1}{2} \end{array}$$ (3)

where $V_{w}^{\prime }$ is the weighted vector image with doubled angles, and *W* is the weight image, which is equal to the nuclear count at each point. The right-hand side of Eq (3) contains values which have previously been computed, with the exception of the image *G* * *W*, which is generated in the same manner as $G*V_{w}^{\prime }$, and therefore does not increase computational workload by a substantial margin. This form allows the evaluation of *f*(*p*, *q*) for a large number of pairs of points with relatively low computational effort.

To obtain Eq (1), consider the following expansion of the first term in the numerator in Eq (3):

$$\begin{array}{c} \begin{array}{cc} |\left(G*V_{w}^{\prime }\right)\left(p\right)+\left(G*V_{w}^{\prime }\right)\left(q\right){|}^{2} & ={\left|\sum_{k}g_{k}\left(w\left(p_{k}\right)v_{k}^{\prime }+w\left(q_{k}\right)u_{k}^{\prime }\right)\right|}^{2}\\ & =\sum_{jk}g_{k}g_{j}\left(w\left(p_{k}\right)v_{k}^{\prime }+w\left(q_{k}\right)u_{k}^{\prime }\right)\cdot \left(w\left(p_{k}\right)v_{k}^{\prime }+w\left(q_{k}\right)u_{k}^{\prime }\right)\\ & =\sum_{jk}g_{k}g_{j}w\left(p_{k}\right)w\left(p_{j}\right)v_{k}^{\prime }\cdot v_{j}^{\prime }+\sum_{jk}g_{k}g_{j}w\left(q_{k}\right)w\left(q_{j}\right)u_{k}^{\prime }\cdot u_{j}^{\prime }\\ & +2\sum_{jk}g_{k}g_{j}w\left(p_{k}\right)w\left(q_{j}\right)v_{k}^{\prime }\cdot u_{j}^{\prime }\\ & =I_{w}\left(p\right)+I_{w}\left(q\right)+2\sum_{jk}g_{k}g_{j}w\left(p_{k}\right)w\left(q_{j}\right)v_{k}^{\prime }\cdot u_{j}^{\prime }\\ & =I_{w}\left(p\right)+I_{w}\left(q\right)+2\sum_{jk}g_{k}g_{j}w\left(p_{k}\right)w\left(q_{j}\right)\left(2{\left(v_{k}\cdot u_{j}\right)}^{2}-1\right). \end{array} \end{array}$$

Now,

$$\begin{array}{c} \begin{array}{cc} \frac{|\left(G*V_{w}^{\prime }\right)\left(p\right)+\left(G*V_{w}^{\prime }\right)\left(q\right){|}^{2}-I_{w}\left(p\right)-I_{w}\left(q\right)}{4(G*W)(p)(G*W)(q)}+\frac{1}{2} & =\frac{\sum_{jk}g_{k}g_{j}w\left(p_{k}\right)w\left(q_{j}\right)\left(2{\left(v_{k}\cdot u_{j}\right)}^{2}-1\right)}{2\sum_{nm}g_{n}g_{m}w\left(p_{n}\right)w\left(p_{m}\right)}+\frac{1}{2}\\ & =\frac{\sum_{jk}g_{k}g_{j}w\left(p_{k}\right)w\left(q_{j}\right){\left(v_{k}\cdot u_{j}\right)}^{2}}{\sum_{nm}g_{n}g_{m}w\left(p_{n}\right)w\left(p_{m}\right)}\\ & =f(p,q), \end{array} \end{array}$$

which establishes Eq (1).

The watershed determined by using this grey image and comparison function represents the edges of fibrous structures. An example of the full effect of this algorithm is given in Fig 22. This watershed (Fig 22C) is combined with the edges found for the *vertical* pixels (Fig 22B), to give the final edge image as illustrated in Fig 22D. These edges can be used to determine structures in the slides before and after registration, which can be subsequently used to verify that the elastic registration has not substantially deformed the tissue.

#### Segmentation in three dimensions

To generate the grey volume and comparison function to be used in the watershed algorithm in three dimensions, a procedure is employed that is analogous to how these quantities are determined for the *planar* pixels in the two-dimensional case. The main difference between these two situations is that in the neighbourhood given in Eq (1), the three-dimensional direction vectors will generally not be contained in the same plane, and therefore a three-dimensional implementation of the previous double-angle technique is required.

The three-dimensional reconstruction *V*_*w*_ consists of a set of voxels to each of which a vector and weight have been assigned. These direction vectors are either zero, where there is no tissue present, or they are unit vectors in the direction of the tissue in the given voxel. The assigned weight represents nuclear density and homogeneity in the tissue at the given voxel. A three-dimensional direction vector *v* can be written in terms of spherical polar coordinates, as follows:

$$\begin{array}{c} v=(\cos\theta \sin\phi ,\sin\theta \sin\phi ,\cos\phi ), \end{array}$$

where *ϕ* ∈ [0, 180] and *θ* ∈ [0, 360). Consider the vector *v*′, given by

$$\begin{array}{c} v^{\prime }=\left(\cos\theta \sin\left(2\phi \right),\sin\theta \sin\left(2\phi \right),\cos\left(2\phi \right)\right). \end{array}$$

**Fig 25:**
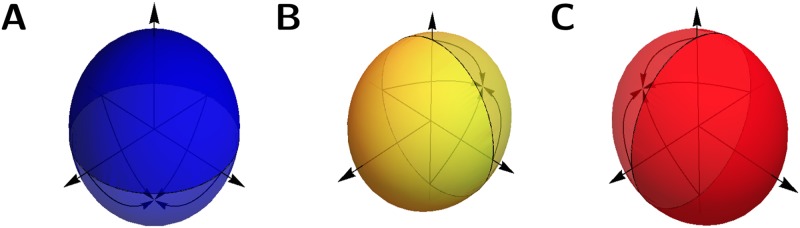
Three double angle transformations in three dimensions. The darker colours represent untransformed vectors, taken to be positive in the relevant index. These hemispheres are stretched by the doubling of the angles, which causes the domain to encompass the whole sphere.

This double-angle transformation is illustrated in Fig 25A, and has the effect of doubling the angle that *v* makes with the *z*-axis. The analogous transformations shown in Fig 25B and 25C represent the doubling of the angle that *v* makes with the *y*- and *x*-axes resepectively. Applying each of these transformations to the volume *V* generates three volumes *V*^(*i*)^, where *i* ∈ {0, 1, 2} indicates the axis along which the angle is doubled.

Consider the convolution of *V*^(*i*)^ with the three-dimensional Gaussian kernel *G*:

$$\begin{array}{c} \left(G*V^{\left(i\right)}\right)\left(p\right)=\sum_{k}g_{k}v_{k}^{\left(i\right)}, \end{array}$$

where *g*_*k*_ is the entry of the Gaussian kernel corresponding to the point *p*_*k*_, $v_{k}^{\left(i\right)}$ is the direction vector of *p*_*k*_ in *V*^(*i*)^, and the set {*p*_*k*_} is the set of points in the window about *p*. Taking the norm squared of this vector gives:

$$\begin{array}{c} \begin{array}{cc} |\left(G*V^{\left(i\right)}\right)\left(p\right){|}^{2} & ={\left|\sum_{k}g_{k}v_{k}^{\left(i\right)}\right|}^{2}\\ & =\left(\sum_{j}g_{j}v_{j}^{\left(i\right)}\right)\cdot \left(\sum_{k}g_{k}v_{k}^{\left(i\right)}\right)\\ & =\sum_{jk}g_{j}g_{k}v_{j}^{\left(i\right)}\cdot v_{k}^{\left(i\right)}. \end{array} \end{array}$$

Let the angles (*θ*_*j*_, *ϕ*_*j*_) and (*θ*_*k*_, *ϕ*_*k*_) denote the spherical polar coordinates for *v*_*j*_ and *v*_*k*_ respectively, with *ϕ* corresponding to the angle from axis *i*. The scalar product in the above sum is given by

$$\begin{array}{c} \begin{array}{cc} v_{j}^{\left(i\right)}\cdot v_{k}^{\left(i\right)} & =\cos\theta_{j}\;\sin\left(2\phi_{j}\right)\cos\theta_{k}\sin\left(2\phi_{k}\right)+\sin\theta_{j}\;\sin\left(2\phi_{j}\right)\sin\theta_{k}\;\sin\left(2\phi_{k}\right)\\ & +\cos\left(2\phi_{j}\right)\;\cos\left(2\phi_{k}\right)\\ & =\cos\left(2\phi_{j}\right)\cos\left(2\phi_{k}\right)+\sin\left(2\phi_{j}\right)\sin\left(2\phi_{k}\right)\cos\left(\theta_{j}-\theta_{k}\right)\\ & =\left(2\cos^{2}\;\phi_{j}-1\right)\left(2\cos^{2}\;\phi_{k}-1\right)+4\cos\;\phi_{j}\;\cos\;\phi_{k}\;\sin\;\phi_{j}\;\sin\;\phi_{k}\;\cos\left(\theta_{j}-\theta_{k}\right)\\ & =\left(2v_{ji}^{2}-1\right)\left(2v_{ki}^{2}-1\right)+4v_{ji}v_{ki}\sin\phi_{j}\sin\phi_{k}\cos\left(\theta_{j}-\theta_{k}\right) \end{array} \end{array}$$ (4)

where v_*i_ is the *i*th component of v_*_. The scalar product *v*_*j*_ ⋅ *v*_*k*_ is given by

$$\begin{array}{c} \begin{array}{cc} v_{j}\cdot v_{k} & =\cos\theta_{j}\;\sin\phi_{j}\cos\theta_{k}\;\sin\phi_{k}+\sin\theta_{j}\;\sin\phi_{j}\sin\theta_{k}\;\sin\phi_{k}+\cos\phi_{j}\cos\phi_{k}\\ & =v_{ji}v_{ki}+\sin\phi_{j}\sin\phi_{k}\cos\left(\theta_{j}-\theta_{k}\right). \end{array} \end{array}$$

Substituting this into Eq (4) yields:

$$\begin{array}{c} \begin{array}{cc} v_{j}^{\left(i\right)}\cdot v_{k}^{\left(i\right)} & =\left(2v_{ji}^{2}-1\right)\left(2v_{ki}^{2}-1\right)+4v_{ji}v_{ki}\left(v_{j}\cdot v_{k}-v_{ji}v_{ki}\right)\\ & =4v_{ji}^{2}v_{ki}^{2}-2v_{ji}^{2}-2v_{ki}^{2}+1+4v_{ji}v_{ki}v_{j}\cdot v_{k}-4v_{ji}^{2}v_{ki}^{2}\\ & =4v_{ji}v_{ki}v_{j}\cdot v_{k}-2v_{ji}^{2}-2v_{ki}^{2}+1. \end{array} \end{array}$$

Define the volume image *I* by

$$\begin{array}{c} I\left(p\right)≔\sum_{i}{\left|\left(G*V^{\left(i\right)}\right)\left(p\right)\right|}^{2}. \end{array}$$

Applying the above formulation of the scalar product to each *i* yields

$$\begin{array}{c} \begin{array}{cc} I(p) & =\sum_{i}\sum_{jk}g_{j}g_{k}\left(4v_{ji}v_{ki}v_{j}\cdot v_{k}-2v_{ji}^{2}-2v_{ki}^{2}+1\right)\\ & =\sum_{jk}g_{j}g_{k}\left(4v_{j}\cdot v_{k}\sum_{i}v_{ji}v_{ki}-2\sum_{i}v_{ji}^{2}-2\sum_{i}v_{ki}^{2}+3\right)\\ & =\sum_{jk}g_{j}g_{k}\left(4{\left(v_{j}\cdot v_{k}\right)}^{2}-1\right) \end{array} \end{array}$$ (5)

where |*v*_*j*_| = |*v*_*k*_| = 1 has been used. A factor of 4 is present here, in contrast to the factor of 2 in found in the two-dimensional case, because each term of the sum in Eq (5) is the sum of three sets of scalar products. Suppose two unit vectors *v* and *u* are situated in the (*x*, *y*)-plane. The double-angle transforms from the *x*- and *y*-axes are the same as the two-dimensional double-angle transform:

$$\begin{array}{c} v^{\left(0\right)}\cdot u^{\left(0\right)}=v^{\left(1\right)}\cdot u^{\left(1\right)}=v^{\prime }\cdot u^{\prime }, \end{array}$$

where *v*′ and *u*′ are the two-dimensional double angle transforms used in two-dimensional segmentation. The double-angle transform from the *z*-axis maps both vectors to (0, 0, −1), so that

$$\begin{array}{c} v^{\left(2\right)}\cdot u^{\left(2\right)}=1. \end{array}$$

Summing these double angles gives

$$\begin{array}{c} \sum_{i=0}^{2}v^{\left(i\right)}\cdot u^{\left(i\right)}=2v^{\prime }\cdot u^{\prime }+1. \end{array}$$ (6)

Eq 6 shows the relationship between the two- and three-dimensional cases and motivates the definition of the image $\tilde{I}$, given by

$$\begin{array}{c} \tilde{I}\left(p\right)≔\left(I\left(p\right)-1\right)/2. \end{array}$$

This image is compatible with Eq (1), and if the vectors in *V* are confined to one plane, Eq (6) ensures that $\tilde{I}$ is equivalent to the two-dimensional grey image.

The volume is weighted by the weight volume *W*, which can be used to modify the vector volumes *V*^(*i*)^, generating the weighted vector volumes $V_{w}^{\left(i\right)}$:

$$\begin{array}{c} V_{w}^{\left(i\right)}\left(p\right)=W\left(p\right)V^{\left(i\right)}\left(p\right). \end{array}$$

The weighted volume *I*_*w*_ is given by

$$I_{w}\left(p\right)≔\left(\underset{i}{\sum }|\left(G*V_{w}^{\left(i\right)}\right)\left(p\right){|}^{2}-\left(G*W\right)\left(p\right)\right)/2$$

which can be rewritten in terms of scalar products of vectors:

$$\begin{array}{c} I_{w}\left(p\right)=\sum_{jk}g_{j}g_{k}w_{k}w_{j}\left(2{\left(v_{j}\cdot v_{k}\right)}^{2}-1\right) \end{array}$$

where *w*_*k*_ is the weight at *p*_*k*_, and all other terms are as above, and this is of the form given in Eq (1).

The comparison function *f* given by Eq (2) can be rewritten as

$$\begin{array}{c} \begin{array}{cc} f(p,q) & =\frac{\sum_{i}{\left|\left(G*V_{w}^{\left(i\right)}\right)\left(p\right)+\left(G*V_{w}^{\left(i\right)}\right)\left(q\right)\right|}^{2}}{8(G*W)(p)(G*W)(q)}\\ & -\frac{2I_{w}\left(p\right)-\left(G*W\right)\left(p\right)-2I_{w}\left(q\right)-\left(G*W\right)\left(q\right)}{8(G*W)(p)(G*W)(q)}+\frac{1}{4}, \end{array} \end{array}$$

in a similar manner as in the two-dimensional case. Here, the threshold for *f* was set to 0.5.

One final modification to the above process for three-dimensional segmentation is the incorporation of adaptive smoothing. The Gaussian windows in Eqs (1) and (2) are of arbitrary size; furthermore, the above formulation of the watershed algorithm can be used with a smoothing kernel that varies in size with position. This means that the Gaussian mask can be set locally to optimise the smoothing for each point. If a point is contained in a wide bundle, then a large smoothing kernel would allow greater noise reduction. Conversely, if a point were contained in a narrow bundle, then a small smoothing kernel would reduce the blurring of the boundaries of the bundle.

The particular adaptive smoothing method used here was previously described by Gomez [27]. The aim is to select Gaussian filters to minimise the following expression at each point *p*:

$$\begin{array}{c} d=\frac{\lambda }{\sigma {\left(p\right)}^{2}}+\epsilon {\left(p\right)}^{2}, \end{array}$$

where *λ* is a constant, *σ*(*p*)^2^ is the variance of the Gaussian at *p*, and *ϵ*(*p*)^2^ is given by

$$\begin{array}{c} \sum_{q\in N_{p}}\sum_{i}{\left|G\left(q\right)*V_{w}^{\left(i\right)}\left(q\right)-V_{w}^{\left(i\right)}\right|}^{2}, \end{array}$$

where *N*_*p*_ is the first-order neighbourhood of voxels. The minimisation is performed by computing the value of *d* for a range of values *σ* at each point, and selecting the value of *σ* corresponding to this minimum. The parameter values used for this adaptive smoothing are listed in Table 5.

**Table 5:** Parameter values used for adaptive smoothing.

Parameter	Value
*λ*	1.22
*σ*_min_	0.4
*σ*_max_	4.0
Δ*σ*	0.1

**Fig 26:**
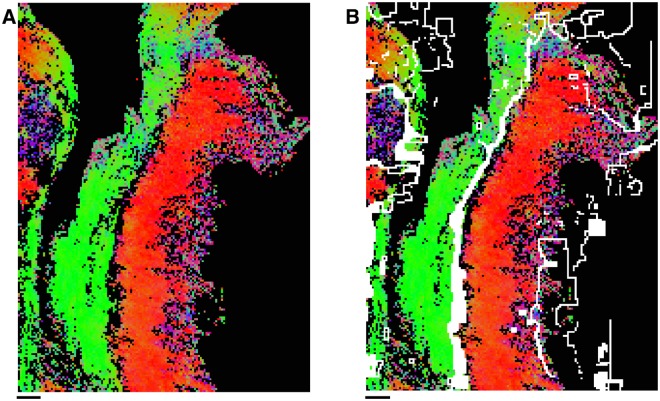
Three-dimensional segmentation. Here the image has been pseudocoloured by direction, with red representing the *x*-component, green representing the *y*-component, and blue representing the *z*-component. The boundaries shown in white divide areas where the bundle direction is substantially different, while avoiding segmentation of locally isotropic areas. Scale bars represent 1 mm.

The adaptive Gaussian mask generated in this manner refines the smoothing used in the edge detection by optimising the kernel size locally. This in turn leads to a more precise segmentation. This completes the process of bundle segmentation in three dimensions. The edges generated by this process have been constructed to ensure that they completely encase each fibrous structure. The full effect of this edge detection algorithm is shown in Fig 26.

### Phase VIII: Isolating smooth muscle tissue

The weighting functions enable the suppression of areas of nuclear density and homogeneity that are uncharacteristic of smooth muscle tissue. The aim is to remove vasculature and placental tissue to ensure that the reconstruction is composed exclusively of myometrial smooth muscle. This was achieved by identifying characteristics of the tissue which differ between myometrial smooth muscle and tissue types.

#### Identifying vasculature

**Fig 27:**
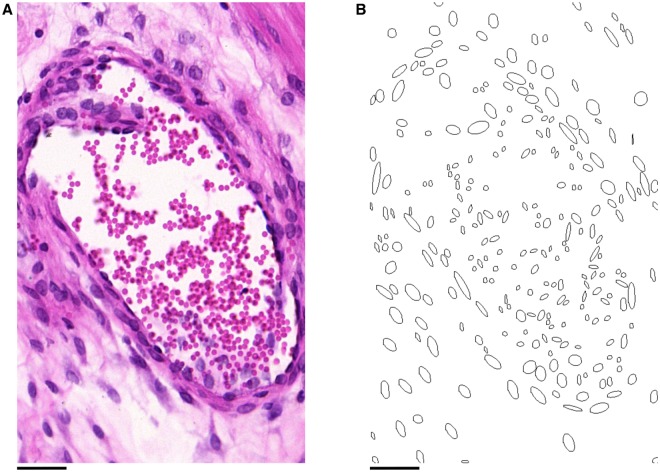
Red blood cell detection. Normally these nuclei are not included in structural computation because they lie within a size range which is below the threshold used for smooth muscle cell nuclei. Scale bars represent 20 *μ*m.

The weighting functions, while useful for enhancing the contrast between smooth muscle tissue and connective tissue, do not differentiate between myometrial and vascular smooth muscle. In order to locate the vasculature, an alternative approach was required. The image analysis methods for detecting nuclei also detected red blood cells, as shown in Fig 27, because red blood cells are located in space which contains no other cells for the algorithm to use as a background reference. The red blood cells were filtered out of the main tissue detection algorithm when the nuclei are thresholded by size, but were still recorded. This means they could be located applying an upper threshold to only include the lower size range in which they are present. Using this threshold to locate red blood cells, the proportion of cells within a voxel which were deemed red blood cells was recorded. A Gaussian mask was applied to the red blood cell density image. This smoothed image was thresholded to obtain potential locations for blood vessels. These points form clusters in the volume, and clusters with sufficiently large size were deemed to be points within blood vessels. These interior points were dilated by one voxel to include the tissue forming the wall of the vessels. While this technique provided some information on the location of blood vessels, it was not sufficient to remove all vascular smooth muscle. This is because not all blood vessels were filled with red blood cells at the time of sectioning to provide an accurate set of interior points. The intention of this process, however, was not to completely eradicate all blood vessels, but rather to reduce the impact of vascular smooth muscle on the overall structure so that it introduced fewer erroneous connections between myometrial smooth muscle fibres. The parameter values used in this process are listed in Table 6.

**Table 6:** Parameter values for detecting vasculature.

Parameter	Value
Maximum red blood cell size	14 *μ*m^2^
Minimum vessel size	9 voxels
Gaussian variance	1.0 voxels

Cell size is measured in the original histological images, vessel size is measured in terms of the voxels used in the reconstruction.

#### Identifying placental tissue in the rat

**Fig 28:**
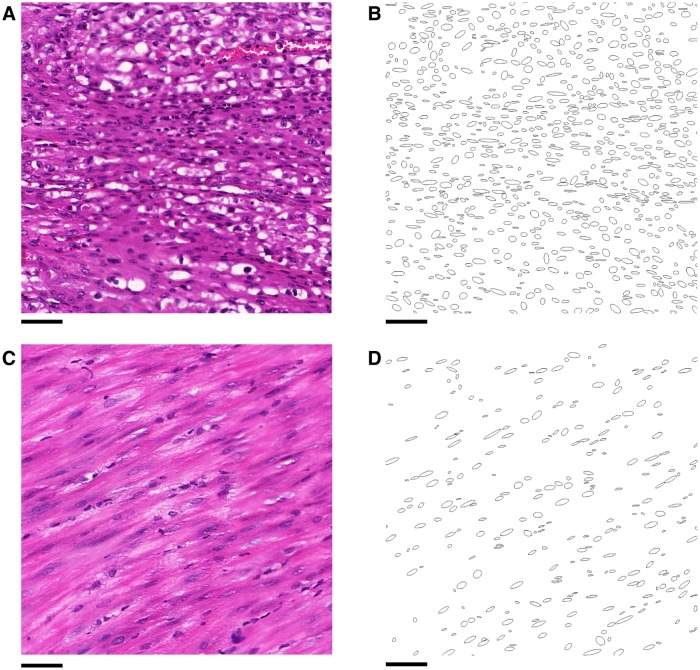
Nuclei detected in the placenta. Nuclei detected in placental tissue (**A-B**), compared to smooth muscle tissue (**C-D**). The nuclear orientation in the placental tissue (**B**) is locally homogeneous, making local anisotropy a poor measure for distinguishing placental tissue from smooth muscle tissue (**D**). The nuclei are, however, much more closely spaced than in smooth muscle (**D**), which enables differentiation of the voxels contained in the placental tissue. Scale bars represent 50 *μ*m.

The final technique used to delimit the reconstructions to myometrial smooth muscle tissue is specific to the pregnant rat uteri being processed, namely the detection of placental tissue. Generally, local heterogeneity of the tissue is not sufficient to identify such tissue, as shown in Fig 28. It was therefore necessary to determine the location of the sites using an alternative attribute. The density of nuclei within the placenta is greater than in smooth muscle tissue, as can be seen in Fig 28. Accordingly, nuclear density was used to identify placental tissue.

The nuclei used to generate the regional representation of the tissue are taken from a restricted size range, which represents the range of typical nuclear sizes found in smooth muscle tissue. For the purpose of detecting placental tissue, the range of sizes was increased to include all nuclei detected in the histological images. The inclusion of smaller nuclei enabled the inclusion of red blood cells which were present in vasculature within the placenta, while larger nuclei were included because this range is occupied with clusters, which are more likely to occur in areas of high nuclear density, which is typical of the placental tissue. The nuclear density of a voxel was defined to be the total number of nuclei from this extended range contained within the voxel. The threshold given in Table 7 was subsequently applied to these voxels to obtain a binary volume.

**Table 7:** Parameter values for identifying implantation sites.

Parameter	Value
Minimum density	4500 nuclei per mm^2^
Lower threshold	0.3
Upper threshold	0.6
Minimum cluster size	10000 voxels

Threshold values here are based on voxel values within the range [0, 1].

The aim of the remaining steps of this process is to identify large clusters of voxels that correspond to the placental tissue in the implantation sites. The binary volume was smoothed by using a large Gaussian kernel with *σ* = 4 voxels. This volume was subjected to a hysteresis thresholding procedure, as is used in Canny edge detection [22]. Hysteresis thresholding generates connected sets of points which all have a value above a lower threshold, and each connected set contains at least one point above an upper threshold. The upper and lower thresholds used here are given in Table 7. All such clusters above the size threshold given in Table 7 were deemed to be sufficiently large to represent implantation sites. These large clusters of voxels with high nuclear density were taken to be the placental tissue.

### Phase IX: Smoothing myometrial smooth muscle direction

**Fig 29:**
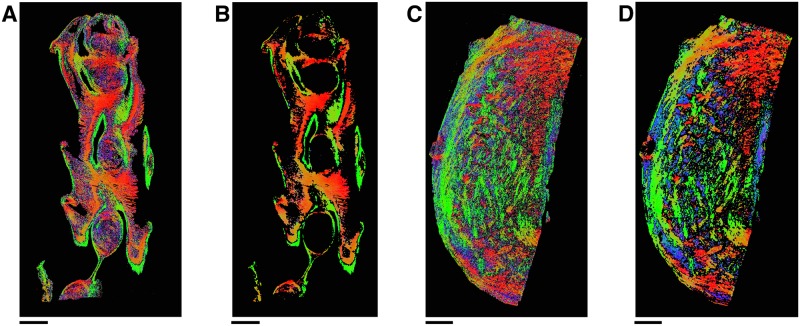
Isolating myometrial smooth muscle. The combined effect of bundle segmentation, tissue segmentation, and smoothing on the structure on a slide from rat myometrium (**A** & **B**) and a slide from human myometrium (**C** & **D**). Images are pseudocoloured by direction: red represents bundles oriented horizontally within the image plane, green represents bundles oriented vertically within the image plane, and blue represents bundles oriented perpendicular to the image plane. **A**: Rat bundle directions prior to segmentation and smoothing. **B**: Rat bundle directions after segmentation and smoothing. Note that the implantation sites have been marked as non-myometrial tissue. **C**: Human bundle directions prior to segmentation and smoothing. **D**: Human bundle directions after segmentation and smoothing. Scale bars represent 5 mm.

Using the various volume representations of various features in the tissue as outlined in the foregoing, it is now possible to obtain the final reconstruction of the myometrium. The process of obtaining this representation began by taking the original vector-valued volume and removing points corresponding to edges, blood vessels, and placenta (if present). The vectors in this volume were then multiplied by the weighting function, *w*. This vector volume was smoothed by convolving with a Gaussian kernel of radius 1 voxel, but, importantly, all points identified as edge points were excluded from this smoothing to maintain boundaries between distinct bundles. The directions of the resulting vectors are taken to be the fibre direction, with weights equal to the lengths of the vectors. The effect of this process is shown in Fig 29.

For the purpose of detecting bundle widths in registered and unregistered slides, it was also necessary to perform a similar operation on these two-dimensional slides. Since the registered slides directly correspond to the representative slides in the volume, the locations of vasculature and implantation sites correspond to the same features in the registered slides. These features were projected back onto the unregistered slides by identifying the points in the unregistered slides that were transformed to points corresponding to these features during the registration process. These features and the edges found by the segmentation algorithm were removed from the tissue for the purposes of measuring bundle widths. The remaining planar directions were subsequently smoothed using a Gaussian kernel of radius 1 pixel. These smoothed *planar* pixels were used to measure bundle width.

This concludes the process of generating the *in silico* representation of the myometrium. The following section will present a means to verify the accuracy of the registration process and analyse bundle widths in the reconstructed myometrium.

### Phase X: Measuring the widths of bundles

This section describes the process for measuring the bundle widths in two and three dimensions. These measurements enable two things: comparison of the bundle widths in registered and unregistered two-dimensional slides provides verification of the registration methods, while comparison of bundle widths in various areas of the three-dimensional reconstruction provides insight into variation in smooth muscle architecture throughout the tissue. The process for determining these width measurements followed the same general form in both cases: points were selected at random in the structure and the width was measured by drawing a line perpendicular to the tissue direction.

**Fig 30:**
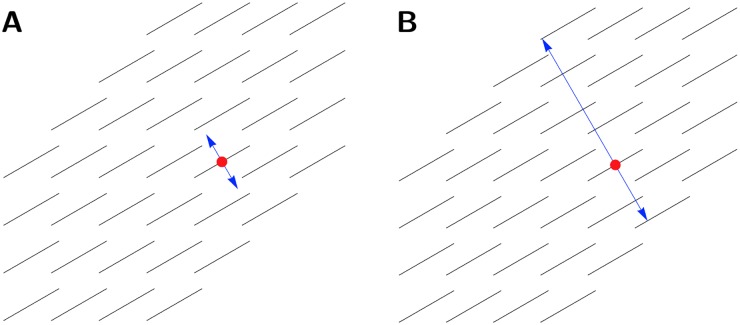
Measuring bundle widths using directional data. **A**: The direction of the width measurement taken at the grid point shown is perpendicular to the regional direction at the given point. **B**: The width measurement is taken by extending the line out from the initial grid point in both directions until it reaches the edge of the bundle.

The process for measuring bundle width in two dimensions is as follows. The smoothed regional representation of the slides was used, as described in the previous section. A uniform grid of predetermined separation was placed at random on the tissue image. If the pixel at a given grid point was classified as *planar* in the smoothed regional representation, a measurement was recorded at that point as follows. The direction perpendicular to the planar direction of the pixel was used for width measurement. This direction selection is illustrated in Fig 30A. The length of the line segment along this width direction through the centre of the given pixel which was contained in *planar* pixels within the tissue was taken to be the width measurement at this point, as illustrated in Fig 30B. These width measurements are taken for each of the registered and unregistered slides, giving a set of width measurements in two dimensions for the whole tissue block in both cases. The distributions of these widths were compared to determine if the registration process had substantially deformed the tissue away from its original shape.

The process for measuring bundle width in the three-dimensional volume was performed in much the same manner as the two-dimensional case. The volume being measured is the smoothed volume described in the previous section. Voxels with weight below a threshold of 0.2 were excluded for the purpose of these measurements. This step is necessary in three dimensions because, in contrast to the two-dimensional case, no threshold was applied to the weights during the segmentation process. A randomly positioned lattice of predetermined width was placed on the volume, and the width was measured at each intersection point of the lattice with an assigned direction vector. For a given point, the width direction is selected at random from the set of directions perpendicular to the tissue direction at that point. The line length within the tissue in the given direction is measured in the same manner as the two-dimensional case. These width measurements provide a sample of the bundle widths in the three-dimensional volume.

## Results

The *in silico* reconstructions of rat and human myometrium were generated from histological sections, which were registered and analysed by a semi-automated procedure to obtain the final volume. The slides were coarse-grained into regions with a typical scale of 5 cell widths. These regions were assigned planar directions based on the orientation of nuclei in the given region. This regional representation was used to register the slides to obtain a three-dimensional representation. The registered slides were subsequently coarse-grained in the *z*-direction in order to create a uniformly spaced volume, where each voxel is assigned a three-dimensional direction vector based on the direction of the nuclei within the given voxel and the local bundle architecture. The volume generated in this manner was subsequently segmented to generate the *in silico* reconstruction of the myometrium.

All three-dimensional reconstructions were visualised using 3D Slicer [28] (www.slicer.org), except where noted below. These reconstructions were facilitated by generating DICOM files using the dcm4che package (www.dcm4che.org).

### Reconstructed volume bounds and slide representation

The slides used to generate the final *in silico* tissue reconstructions were selected from the series of 5 *μ*m sections taken from the original tissue blocks. Not all the sections were suitable for use in the reconstruction. The selection of slides was performed during the sectioning process, where slides were discarded if they were deemed beyond the algorithm’s ability to correct the distortion, and during the registration process, where slides were discarded if they failed to register accurately. In addition, the reconstructed volumes all have boundaries which were created by missing sections at the top and bottom of the stack, as well as by clipping the tissue to fit it onto the slides. This section presents the artificial bounds created in this manner, and the proportion of slides from the original stack represented in the final reconstructed volume. The artificial bounds will become important when boundary artefacts are to be considered in the subsequent analysis of the bundle widths. The proportion of slides used in the final reconstructions provides a measure of the reliability of the reconstructed volume. Furthermore, the ratio of slides present before and after registration provides a measure of the effectiveness of the automated registration algorithm, because all slides discarded at this point will be visually identified to have failed to register.

**Fig 31:**
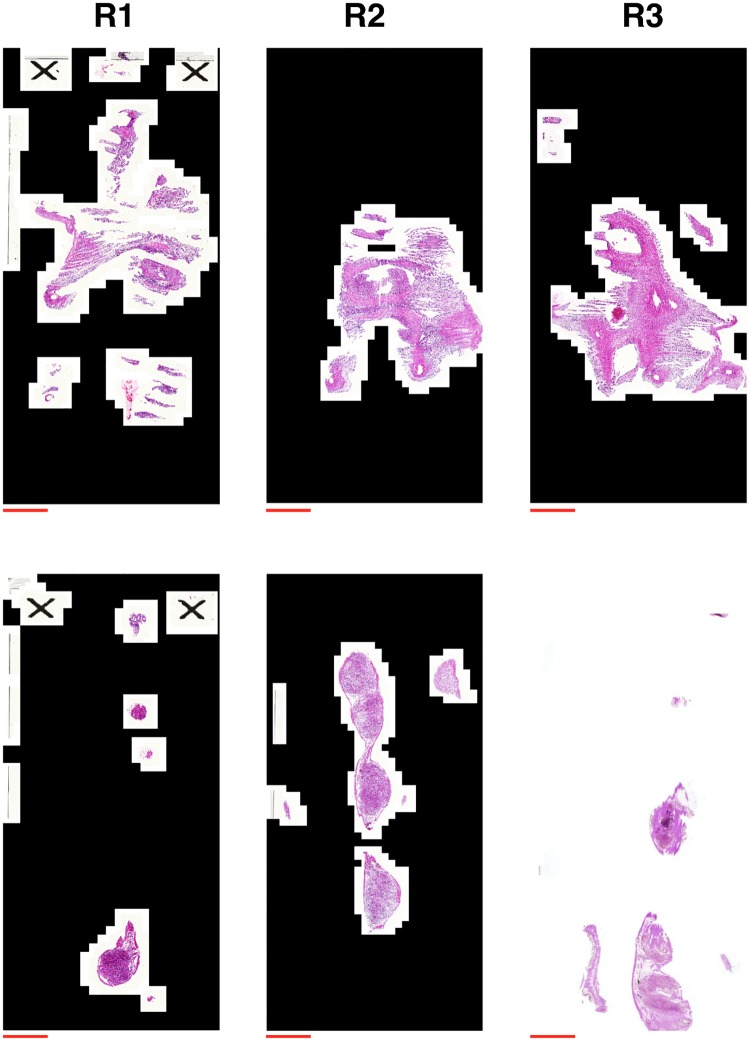
The first and last slides used in the reconstruction of the rat tissue blocks. The amount of tissue located prior to the first slide is unknown due to the lack of numbering prior to the first slide; however, the number of slides discarded prior to the first slide generated was less than 100, which puts an upper bound of 500 *μ*m on the depth lost. The last slide for each tissue block represents the last slide which the registration algorithm could register. In each case, the remaining slides include tissue in and surrounding the placental beds. The amount of tissue remaining in each case is less than 500 *μ*m, which can be verified by the number of slides that could not be registered. Top row: first slides in each of the rat tissue blocks. Bottom row: last slides in each of the rat tissue blocks. R* indicates tissue block number. Red scale bars represent 5 mm.

A special case where slides from the rat tissue block failed to register and were subsequently discarded was at the top of the stack, where the only tissue present was placenta surrounded by a small amount of smooth muscle, as illustrated in the bottom row of Fig 31. In each rat tissue block, the amount of smooth muscle tissue present in the slides will at some point become insufficient for accurate registration. For this reason, the *in silico* reconstructions of the rat tissue blocks do not contain the top-most slides. The reduction in stack size is noted in Table 8, and is equivalent to a depth of roughly 0.5 mm for each tissue block. Similarly, the bottom of the stack, where the sectioning began, is also missing tissue, because a number of slides had to be discarded prior to the first numbered slide. The number of slides lost in this manner cannot be quantified precisely because these slides were not numbered, but is fewer than 100, which is also the approximate number lost at the top of the stack. These two bounds on the volume represent a truncation of the tissue in the final volume representation, and the effect of this truncation will be noticeable in the following width analysis. Similarly, the reconstruction of the human tissue block also represents a volume with artificial bounds at the start and end of the stack, and the effect of this truncation will likewise be observed.

**Table 8:** Number of slides at each step of processing.

Tissue block	Total number of slides	Slides present before registration	Slides present after registration	Proportion of slides used
Rat block 1	389 (485)	221 (271)	141	36%
Rat block 2	411 (488)	237 (279)	174	42%
Rat block 3	418 (442)	201 (210)	118	28%
Human block	711	263	137	19%

Values in brackets indicate the number of slides including the top slides discarded due to an insufficient amount of tissue (rat blocks only, see text for details). Proportion shown is the percentage of the original slides used to construct the final volume.

**Fig 32:**
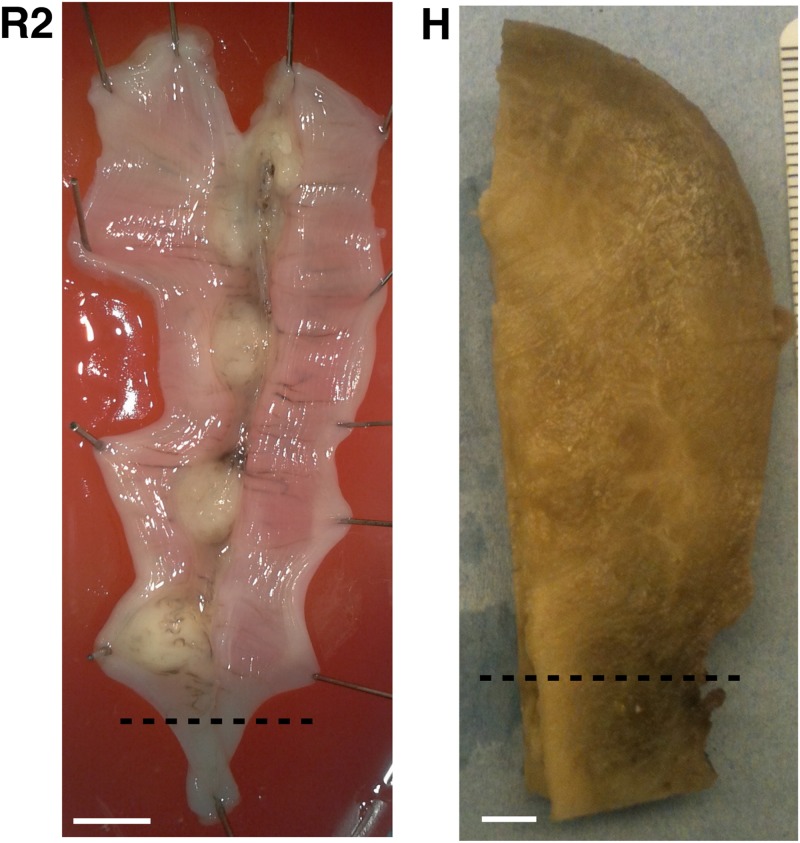
The approximate location of the clipping boundaries. This clipping was applied to the second rat block (**R2**) and human block (**H**) during the sectioning process. The precise boundary varied between slides, but the overall effect was a truncation of the tissue along the dashed lines shown. Scale bars represent 5 mm.

**Fig 33:**
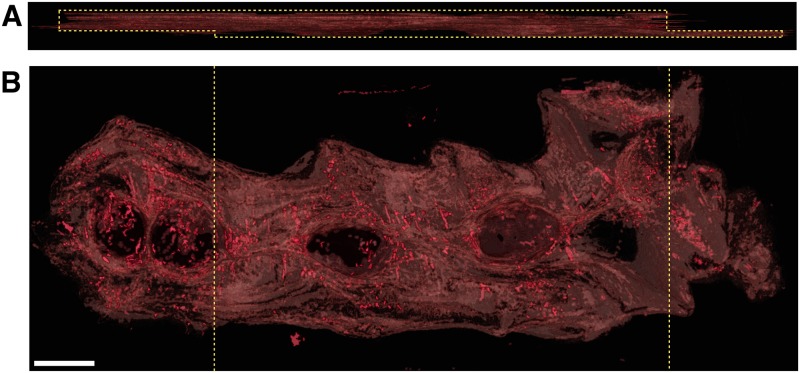
The bounds induced in the third rat tissue block by clipping. Here the tissue was clipped at the ovarian end for the first 120 slides, before transition to clipping at the cervical end. **A**: The approximate bounds created by this switch in clipping. **B**: The approximate locations where the tissue was clipped at each end. Scale bar represents 5 mm. Visualised in Osirix [29] (www.osirix-viewer.com).

The human tissue block, and second and third rat tissue blocks, also had bounds on the cross-sectional area imposed because the tissue was too large to fit entirely on the slides used. In order to allow the tissue to fit on the slides, the tissue was clipped, meaning that any tissue extending over the edge of a slide was trimmed off. The human tissue block was clipped at the cervical end to fit the tissue to the slide, and additionally the tissue block was made to fit the width of the slides by cutting the tissue in two prior to embedding in paraffin. This had the result of creating an artificial bound at the centre of the uterus and a bound at the cervical end, as shown in Fig 32A. Similarly, the second rat tissue block was too long for to fit the length of the slides, and sections were clipped along the line indicated in Fig 32B. The third rat tissue block was also too long for the sections to fit on the slides; however, it was possible to preserve much of the smooth muscle architecture by exploiting the three-dimensional positioning of the tissue in the paraffin block. The cervical end was slightly raised in the block, which caused the initial slides to contain tissue mostly from the cervical end. Accordingly, the ovarian end of the tissue was clipped to obtain the smooth muscle at the cervical end for these slides. As the sectioning progressed, the smooth muscle became less prevalent at the cervical end and more prevalent at the ovarian end. For this reason, the clipping was switched to the cervical end beyond a given slide deemed to be a transition between these two. While this process optimised preservation of the smooth muscle tissue for the reconstruction, the transfer of clipping did introduce slightly more complex artificial bounds, as illustrated in Fig 33. The effect of these bounds will be noted in the following analysis of the bundle widths in the *in silico* reconstruction.

During the sectioning process, it was noted that the final reconstruction did not require the full set of slides in the human tissue block. Accordingly, the number of sections discarded from the human tissue block was increased after the 300th slide to two out of every three in order to reduce the time required to process the slides. This reduced slide count is evident in the number of slides present prior to registration, given in Table 8; however, the effect of this deliberate discarding on the final reconstructed volume is minimal, due to the coarse-graining, as explained below.

Table 8 summarises the number of slides present at each step, and the total number of slides which would have been present, had none of the slides been discarded. About half of the sections in each of the rat tissue blocks were deemed unfit for registration. The proportion of sections discarded in the human tissue block was considerably greater than half; however, this was due to the reduction in slides mentioned in the previous paragraph. Comparison of the number of slides before and after registration shows that the registration process was judged accurate upon visual inspection for more than half the slides present after sectioning in each block: the majority of the slides taken from the original blocks were used to produce the *in silico* reconstruction. As described above, the *in silico* reconstruction was coarse-grained in the *z*-direction, which means that each slide in the final three-dimensional volume represents approximately 10 slides from the original tissue. This coarse-graining means that each slide of the reconstructed tissue is represented by an average of 2–4 slides from the original tissue blocks. This strongly suggests that the *in silico* reconstruction is representative of the tissue throughout the stack, and each slide in the final volume represents a faithful sample of the original tissue.

### Verification of reconstructed tissue

The reconstruction specifically and explicitly represents fibre direction. In order to verify that the final *in silico* version represents the original tissue, the following verification steps were performed. First, to verify the planar direction vectors assigned to the tissue, tiles were sampled at random and were visually compared to the assigned direction vectors. Second, in order to verify that the registration did not deform the original slides beyond a given threshold, the bundle widths in registered and unregistered tiles were compared to establish equivalence. The third verification step was to compare images of the original tissue blocks prior to sectioning with images of the *in silico* reconstruction. This comparison was performed by eye, in order to verify that the shape and features of the reconstructed tissue matched the original tissue.

**Fig 34:**
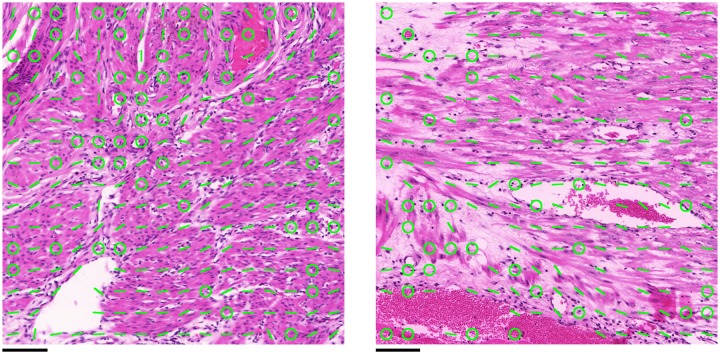
Comparison of assigned directions with histological images. Left: human tissue block. Right: rat tissue block 3. Green lines show indicate the planar orientation detected at that point, while green circles indicate that no planar direction could be assigned to the given point, but the point is still marked as containing nuclei. Scale bars represent 100 *μ*m.

First, the representation of direction was visually compared with the original histological images. This comparison was performed by selecting 16 × 16 pixel (∼1 mm^2^) tiles at random from each of the tissue blocks, and superimposing the detected planar directions onto these tiles. An example of two such tiles is shown in Fig 34, with direction indicated by green lines. The excellent agreement indicates that the planar directions found by the image analysis accurately represent the tissue direction. Areas where no planar direction could be assigned but which do contain nuclei are shown as green circles. These arise when either the cross sections of the detected nuclei closely approximate a circle, so the direction cannot be reliably determined, or when the detected nuclei in the region are associated to direction vectors that vary widely. These regions are categorised as being *vertical*, and are assigned three-dimensional vectors which are at least 30° from the slide plane. The circles in the left tile in Fig 34 generally represent areas where the nuclei have a low eccentricity, which suggests that these nuclei are indeed virtually perpendicular to the slide plane. The circles appear close to the edges of bundles in the right-hand tile where bundle direction is generally ill-defined, which suggests that these regions are categorised as *vertical* because the planar direction of the nuclei is highly variable in the given regions. This high variability means that any direction assigned to these regions would be unreliable, and therefore direction is assigned to these regions later in the process when three-dimensional directions have been assigned. While smooth muscle fibres are well-represented by the direction vectors in the images, it is also evident that a portion of these vectors have in fact been assigned to the vasculature which represents a confounding factor to the *in silico* reconstruction of the myometrial direction field. To eliminate these nuisance vectors, vascular regions are removed later in the processing pipeline by a separate algorithm during **Phase VIII**. The remaining tiles which were compared are given in the supporting information (S1–S16 Figs).

**Fig 35:**
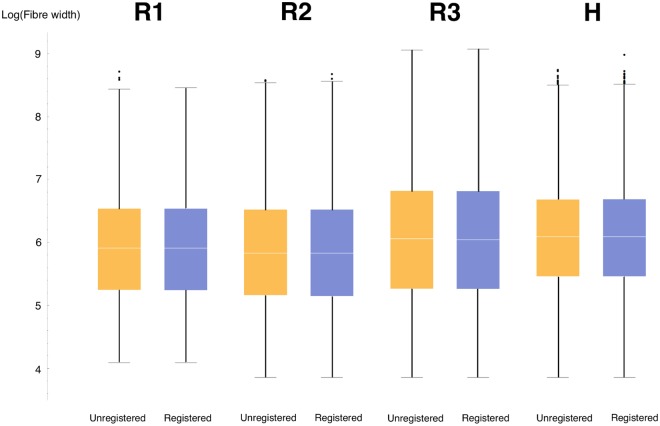
Variability of log-transformed bundle widths in registered and unregistered slides. **R1**, **R2**, and **R3** represent each of the rat tissue blocks, **H** represents the human tissue block. The general similarity in shape between registered and unregistered slides in each block suggests that any deformation incurred by the registration process has little effect on the overall structure of the tissue block.

The second step in the verification process was to compare the registered slides with the unregistered slides, to ensure that the registration procedure has not deformed the reconstructed tissue. The quantity used to compare these slides was *bundle width*. This width was automatically measured at points on a randomly placed grid for each registered and unregistered slide. The grids had an internodal separation of 4 regions, which corresponded to 240 *μ*m in the first rat tissue block, and 190 *μ*m in all other tissue blocks. These measurements were taken from each slide present before and after registration. The distributions of these datasets are positively skewed, so the log transform was applied for the purpose of statistical comparison of the means. The distributions of the resulting log-transformed data sets for registered and unregistered slides in each tissue block are shown as box plots in Fig 35. The log-transformed widths from unregistered and registered slides were compared to test for equivalence of the mean in the following manner. The maximum deformation allowed was set to 5%, meaning that if the means of bundle widths in unregistered and registered slides differ by more than 5%, the registration process is deemed to have substantially deformed the tissue and the tissue is no longer representative of the original slides. The log transform of this effect size is given by the interval

$$\begin{array}{c} (\log(1)-\log(1.05),\log(1)-\log(0.95))=(-0.0488,0.0513). \end{array}$$

The difference in log-transformed means and 95% confidence intervals are shown in Table 9. The confidence intervals are all contained within the above effect size interval, and therefore the deformation incurred is substantially below 5%. This indicates that any deformation incurred by the registration process has not significantly changed the mean bundle width.

**Table 9:** Difference in means of log-transformed width measurements between unregistered and registered slides.

Tissue Block	Mean Log difference	Confidence interval
Rat Block 1	0.00283	(−0.00402, 0.00968)
Rat Block 2	0.00483	(−0.000649, 0.0103)
Rat Block 3	0.00505	(−0.00137, 0.0115)
Human Block	−0.000848	(−0.00313, 0.00144)

Confidence interval represents a 95% probability of being in the given interval. The interval representing deformation of up to 5% is (−0.0488, 0.0513) for all tissue blocks. This interval contains all the above confidence intervals, suggesting that the tissue has been deformed by no more than 5% by the registration process.

To ensure that the distribution of widths was unchanged by the registration process, a bootstrap method was used [30]. For each tissue block, random samples of 1000 values were taken from the set of widths in each of the registered and unregistered slides. For each pair of samples, the Kolmogorov-Smirnov test statistic was calculated [30]. The distribution of these values was used to determine the 90% confidence interval for the actual value of the Kolmogorov-Smirnov test statistic. If the confidence interval was found to lie below the critical value for the Kolmogorov-Smirnov test at a 95% confidence level, the distributions were deemed to be equivalent. The Kolmogorov-Smirnov test statistic is defined to be

$$\begin{array}{c} D_{n,m}≔\underset{x}{\sup}\left|F_{u,n}\left(x\right)-F_{r,m}\left(x\right)\right|, \end{array}$$

where *F*_*u*,*n*_(*x*) is the empirical CDF of a sample of *n* values from the unregistered slide widths and *F*_*r*,*m*_(*x*) is the empirical CDF of a sample of *m* values form the registered slide widths. The probability of interest is

$$\begin{array}{c} P\left(\sqrt{\frac{nm}{n+m}}D_{n,m}<c\left(\alpha \right)\right), \end{array}$$

where *c*(*α*) is the critical value for the Kolmogorov-Smirnov test at significance level *α*. The critical value used in the equivalence test was *c*(0.05) = 1.36 [30]. The values of these tests are summarised in Table 10. The table shows that the above probability is greater than 90% for all tissue blocks, strongly suggesting that the distribution of widths is similar between registered and unregistered tissue blocks.

**Table 10:** Value of the normalised Kolmogorov test statistic for comparing registered and unregistered slides.

Tissue Block	*n*	*m*	*K*_*n*,*m*_	$P(K_{1000,1000}<1.36)$
Rat block 1	242856	239319	0.96	0.9504
Rat block 2	453123	449007	1.56	0.9486
Rat block 3	404011	398464	1.32	0.9544
Human block	2321528	2153015	1.00	0.9532

The probabilities were generated by bootstrap sampling to obtain a distribution of *K*_1000,1000_. The critical value *c*(0.05) = 1.36 represents a 95% confidence interval for the normalised test statistic. The probabilities show that the 90% confidence interval for the value of *K*_*m*,*n*_ in each pair of datasets is contained within the 95% confidence interval, indicating that the pairs of values represent similar distributions.

**Fig 36:**
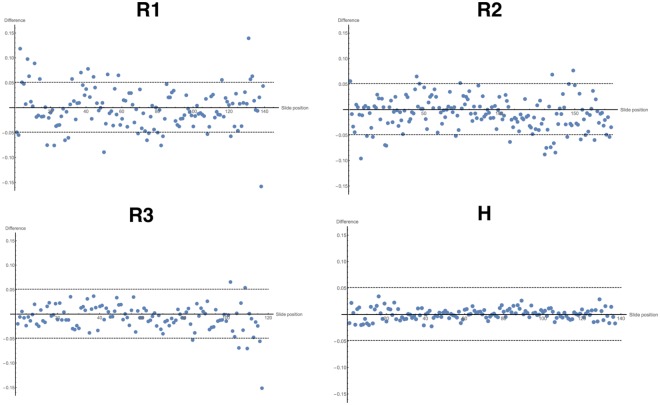
Difference in mean log-transformed bundle widths between registered and unregistered tissue blocks by slide. Dashed lines indicate a 5% change in width. The vast majority of points lie within the 5% bounds for each block, suggesting that even at an individual slide level the deformation is not significant.

While the foregoing confirms that the tissue blocks have not been substantially deformed in general, it is also imperative to determine if any systematic deformation has occurred. This form of deformation would manifest as in incremental deformation of slides away from the global reference slide, caused by minor inaccuracies in registration accumulating toward the ends of the stack. The graphs in Fig 36 show the difference in the mean of the log-transformed widths between registered and unregistered slides as a function of slide position. The dashed lines indicate the values for a deformation of ±5%. In all cases the vast majority of slides lie within these lines, suggesting that any systematic deformation is sufficiently small to be within a 5% effect size, suggesting that systematic distortion was insignificant. The human tissue (Fig 36H) shows deformation well below 5% in all slides, unlike the rat samples. This could be due to the tissue having a roughly convex cross section for most of the slides, yielding a more rigid structure when sectioned.

### Visual comparison at the organ level

**Fig 37:**
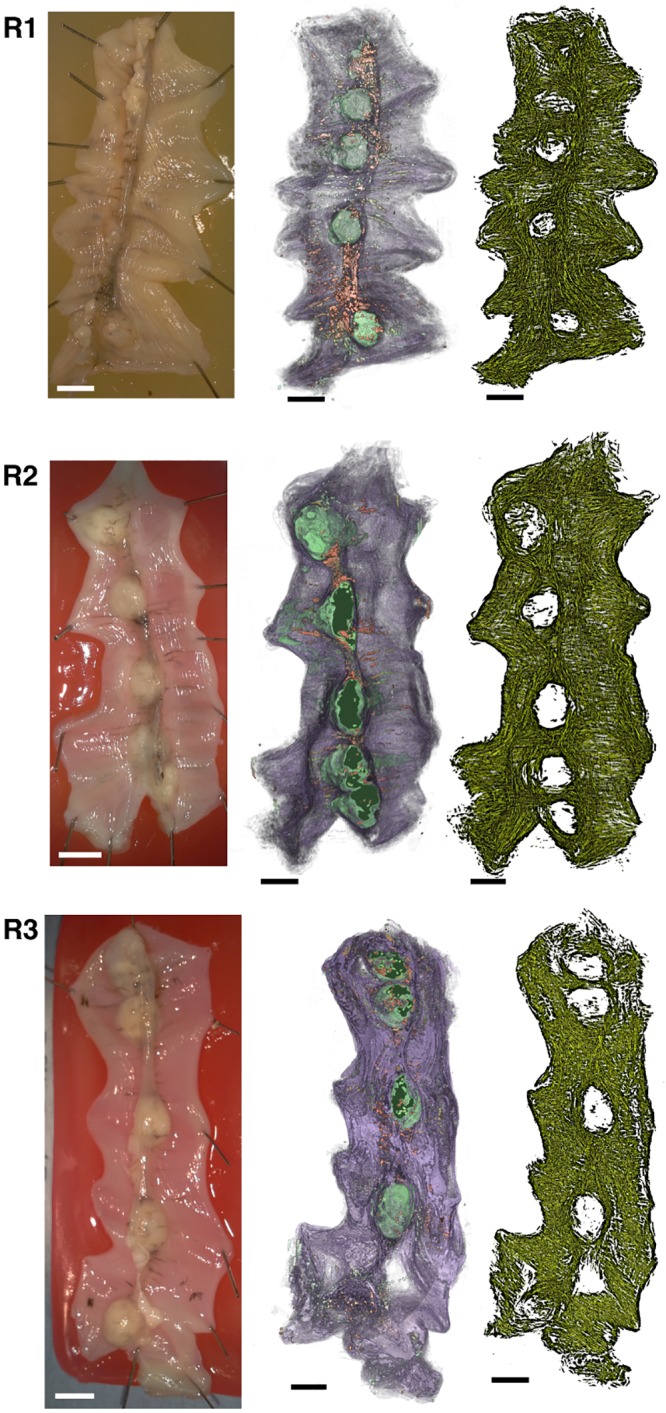
Comparison of the general habitus of the reconstructed tissue with the original tissue in the rat tissue blocks. Each row shows original tissue and representation of the *in silico* reconstructions for each rat tissue block, labelled **R1**–**R3** according to the block number. Left: image of the original tissue block taken between fixation and embedding in paraffin. Centre: representation of the *in silico* reconstruction of the tissue block, where red indicates blood vessels, green indicates implantation sites, and purple indicates smooth muscle tissue. Here the general shape of the tissue and positioning of blood vessels agrees with the original tissue (left-hand panel) in all tissue blocks. Right: bundles in the *in silico* reconstruction, where the tissue is highlighted along the fibre direction from a set of randomly selected seed points (see S1 Appendix for details). These bundles exhibit close correspondence to the fibrous structures visible in the original tissue (left-hand panel) in all tissue blocks. Scale bars represent 5 mm.

**Fig 38:**
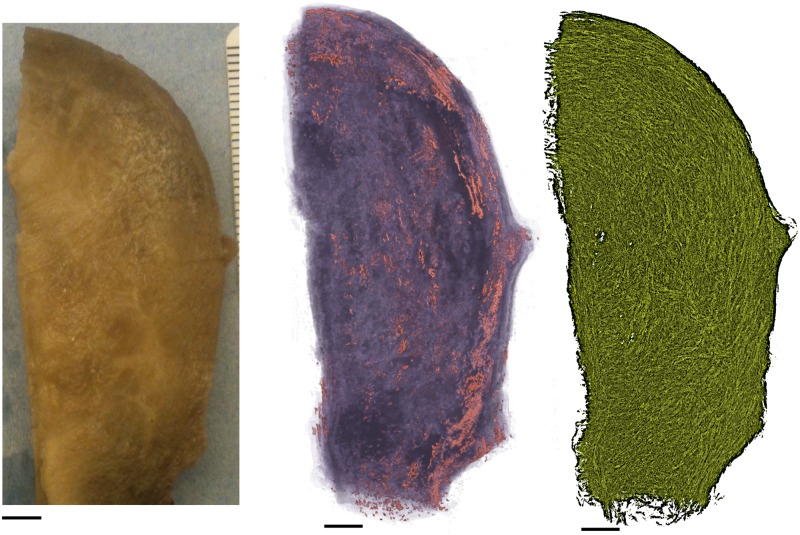
Comparison of the general habitus of the reconstructed tissue with the original tissue in the human tissue block. Left: image of the original tissue block taken between fixation and embedding in paraffin. Centre: representation of the *in silico* reconstruction of the tissue block, where red indicates blood vessels, and purple indicates smooth muscle tissue. The general shape of the tissue aligns with the original tissue block, suggesting that the reconstruction is a good representation of the overall shape of the tissue. Vasculature is less apparent in the human tissue (left) than in the rat samples; however, areas that are densely packed with red blood cells appear darker, for example toward the exterior of the tissue. These dark areas correspond to vasculature detected in the reconstruction (centre). Right: bundles in the *in silico* reconstruction, where the tissue is highlighted along the fibre direction from a set of randomly selected seed points (see S1 Appendix for details). Scale bars represent 5 mm.

Comparison between the original tissue blocks and the *in silico* reconstructions are shown in Figs 37 and 38. This shows that the general habitus of the tissue blocks has been largely maintained in the reconstruction, with slight differences observable at the edges of the tissue. Furthermore, the blood vessels in the rat tissue blocks, visible as darker areas in the original tissue and red in the reconstructed tissue, have also been maintained in the reconstruction. These two similarities between tissue and *in silico* reconstruction suggest that the general shape of the reconstruction represents that of the original tissue blocks.

### Analysis of bundle widths

**Fig 39:**
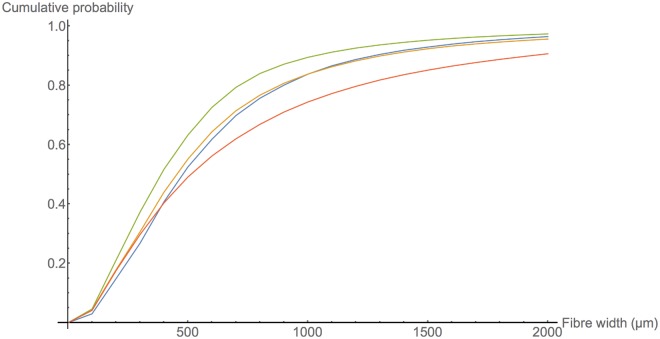
Empirical CDFs of the distribution of bundle widths. Widths in the human tissue block are markedly higher than those in the rat tissue blocks, as indicated by the shift to the right of the distribution. Additionally, the third rat tissue block appears to have smaller widths than the other two rat tissue blocks, as indicated by a shift to the left of the distribution.

In all blocks, the bundle width was measured at nodes of a randomly positioned lattice in a randomly selected direction perpendicular to the direction of the given voxel. The separation of nodes in the lattice was set to 4 voxels in the slide plane, which represents 240 *μ*m in the first rat tissue block and 190 *μ*m in all other tissue blocks, and 1 voxel in the *z*-direction, which represents 60 *μ*m in the first rat tissue block and 47.5 *μ*m in all other tissue blocks. The higher sampling rate in the *z*-direction was required because the depth of the tissue blocks were typically ∼10× smaller than the other dimensions. The distributions of measured bundle widths in the rat and human tissue blocks are compared in Fig 39. The median, 95% confidence interval, and sample size of each of these data sets for each tissue block are listed in Table 11. These measurements were compared in a similar manner to the bundle width comparison performed in the previous section; the data was log-transformed and pairwise comparisons were performed on the log-transformed data sets to test for substantial differences. The difference in means of the log transformed data between widths in each pair of tissue blocks is shown in Table 12, with 95% confidence intervals. This shows that the difference in means between all tissue blocks is non-zero with 95% confidence, which suggests that the bundle widths vary between all tissue blocks.

**Table 11:** Median bundle width for each tissue block.

Tissue Block	Median ± CI (*μ*m)	*n*
Rat Block 1	482 ± 2	91438
Rat Block 2	452 ± 2	189958
Rat Block 3	388 ± 2	166666
Human Block	513 ± 2	922534

Error shown is the 95% confidence interval, rounded up to the nearest *μ*m.

**Table 12:** The difference of means of the log-transformed bundle widths between each tissue block.

Tissue Block	Rat Block 1	Rat block 2	Rat block 3	Human block
Rat Block 1	-	0.051 ± 0.006	0.21 ± 0.006	−0.092 ± 0.006
Rat Block 2	−0.051 ± 0.006	-	0.15 ± 0.005	−0.14 ± 0.004
Rat Block 3	−0.21 ± 0.006	−0.15 ± 0.005	-	−0.29 ± 0.004
Human Block	0.092 ± 0.006	0.14 ± 0.004	0.29 ± 0.004	-

Error shown is 95% confidence interval.

The magnitude of the difference in widths between two tissue blocks *A* and *B* as a proportion of the overall width of *A* is given by

$$\begin{array}{c} 1-\exp(\mu_{A}-\mu_{B}) \end{array}$$

where *μ*_*A*_ and *μ*_*B*_ are the means of the log-transformed widths for the tissue blocks *A* and *B*. The difference in means between rat blocks 1 and 2 corresponds to an decrease in width of 5% ± 1% from block 1 to block 2, which suggests that there is no substantial difference in bundle width between these two blocks. The difference in means between rat block 3 and rat blocks 1 and 2 correspond to a decrease in width of 19% ± 1% from block 1 to block 3, and a decrease in width of 14% ± 1% from block 2 to block 3. This suggests that rat block 3 is substantially different from the other two rat blocks. One confounding factor that might contribute to this difference, however, is the more complex artificial set of boundaries imposed on rat block 3. This effect is apparent in the analysis of bundle widths as a function of depth in the stack, which is discussed in more detail below. The difference in means between the human tissue block and the rat tissue blocks correspond to a decrease in width of 10% ± 1%, 15% ± 1%, and 35% ± 1% from the human block to rat blocks 1, 2, and 3, respectively. This suggests that there is a substantial difference between the human tissue block and the rat tissue blocks. However, the difference between the widths in the human block and rat block 1 is smaller than the difference between rat block 3 and rat block 1, which indicates that the difference observed between human and rat bundle widths may be within the bounds of variation between individuals.

**Fig 40:**
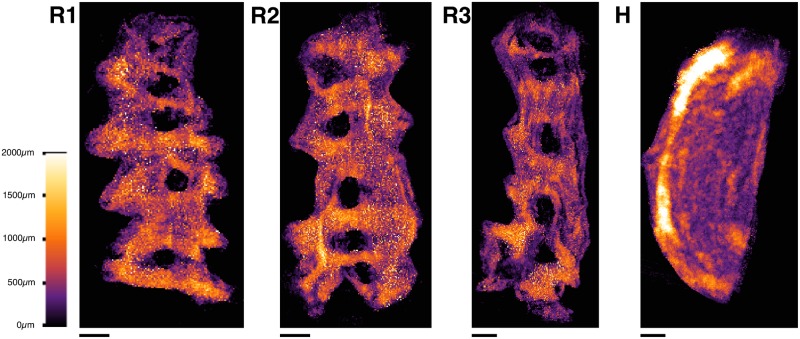
Mean bundle widths along the *z*-axis for each tissue block. The images were generated by taking the median value of recorded widths in the lattice along the *z*-axis, and coloured according to the LUT displayed on the left. **R1**, **R2**, **R3**: Rat tissue blocks 1, 2, and 3 respectively. **H**: Human tissue block. Scale bars represent 5 mm.

The spatial distribution of bundle widths in the slide plane for each tissue block is shown in Fig 40. These images were generated by calculating the median width along the *z*-axis for each point in the grid given by the slide plane cross-section of the lattice and representing this median value according to the lookup table (LUT) shown. In the rat tissue blocks, these indicate that the bundle width is variable throughout the tissue, with some areas of the tissue having larger widths, but no apparent correlation with any specific anatomical feature. In the human tissue block, there does appear to be an area of wider bundles toward the perimetrium, which agrees with previous observations made by Young and Hession [2].

**Fig 41:**
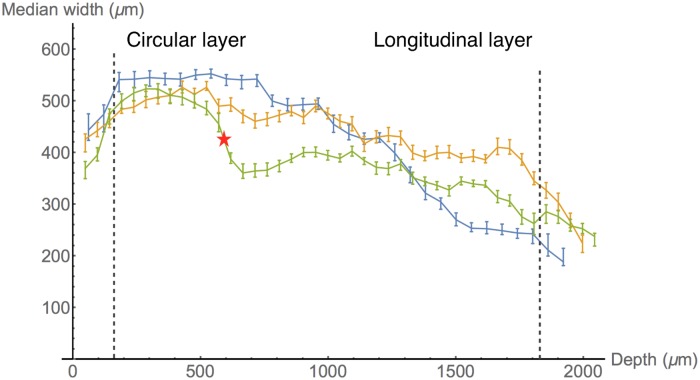
The median widths in the rat tissue blocks as a function of *z*. Each point represents a slide in the *in silico* reconstruction, sorted in increasing slide number such that the lower values represent slides containing mostly circular smooth muscle tissue, while the represent slides containing mostly longitudinal smooth muscle tissue. Blue represents tissue block 1, orange represents tissue block 2, and green represents tissue block 3. The general trend is a reduction in width from circular to smooth muscle, suggesting that bundles are thicker in the circular layer.

**Fig 42:**
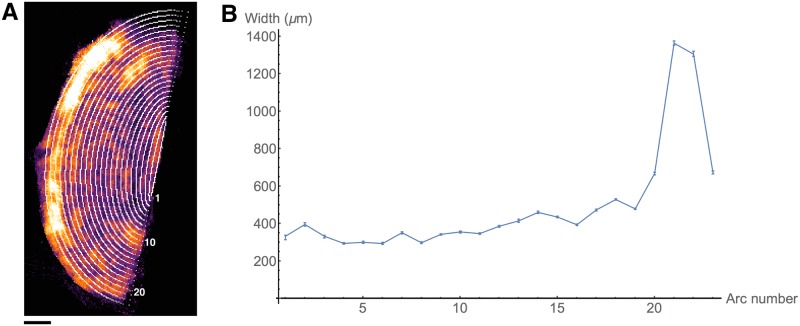
Comparison of bundle widths along arcs located between the perimetrium and endometrium. **A**: The arcs used to compare widths at varying distance from the endometrium. **B**: Median width on each arc. The large peak toward the perimetrium is the layer of subserosal bundles observed by Weiss *et al.* [3], and the values shown here indicate that the bundles are approximately 3 times wider than in the rest of the tissue. Scale bar represents 5 mm.

A comparison of widths by depth in the *z*-direction for the rat tissue blocks is shown in Fig 41. There appears to be a difference in width between the higher and lower bundles in the block, with widths toward the circular end being higher. This suggests that bundles are wider in the circular layer than in the longitudinal layer. The orientation of the human tissue block motivates the comparison of widths along the arcs shown in Fig 42A. These arcs represent steps away from the endometrium, and hence can be used to compare inner and outer layers. The comparison of widths measured along these arcs is shown in Fig 42B. This shows that the median width increases width distance from the endometrium, with a large excursion near the perimetrium. It appears that the bundles are wider in the longitudinal layer than in the circular layer, with larger bundles occupying the outermost portions of the longitudinal layer. This stands in contrast to the observations made in the rat myometrium, which shows larger bundles in the circular layer and thinner bundles in the longitudinal layer.

## Discussion

The methods presented in this paper provide a means for generating high-resolution three-dimensional reconstructions of myometrial smooth muscle from serial sections. The reconstructions generated in this manner have been shown to be representative of the original sections with a high degree of fidelity.

### Bundle widths

Bundle width may affect excitability [31]. The ratio of cell-to-cell coupling to volume is lower in thinner bundles than in thicker bundles: a larger proportion of cells in a thinner bundle is on the edge of the bundle due to the increased surface area to volume ratio, and these cells have uncoupled areas on their surface. This means that the total area available for cell-to-cell coupling per unit volume is lower in thinner bundles than thicker bundles. It has previously been shown that excitation propagation through cell networks with uniformly high levels of cell-to-cell coupling requires a large input of current [32]. This suggests that, if cells are uniformly connected in the tissue, a lower input current would be required to excite thinner bundles than thicker bundles.

The average widths of bundles measured here in the human (513 *μ*m) and rat (388–482 *μ*m) tissues are quite similar. It should be noted, however, that we compared a non-pregnant human to a pregnant rat. In pregnancy, there is proliferation followed by hypertrophy, which in turn may engender reorganisation or remodelling of the tissue microarchitecture.

Bundle width in the inner portion of the human myometrium is lower than the outermost portion, having a median width of 405 ± 1.5 *μ*m (taken from arcs 1–19 in Fig 42), which is within the range of average widths from the rat tissue. The human uterus is approximately 10 times the volume of the rat uterus, but this scaling is not reflected by a similar disparity in typical bundle width. One possible explanation is that the width of smooth muscle fibres in the myometrium has attained a physiological upper limit in humans.

We investigated the spatial distribution of bundle widths in the reconstructions. In the rat myometrium, the bundles were generally wider in the circular layer than in the longitudinal layer. In the human myometrium, a layer of thick bundles was identified in close proximity to the perimetrium, as has been previously observed [3]. Furthermore, the bundles appear become wider as they are located further away from the endometrium. If bundle width affects excitability, as described above, regions in which thinner bundles predominate would constitute the more likely sites of initiation of contraction waves. In the rat myometrium, this would imply that excitation is most likely to be initiated in the longitudinal layer, where bundles are thinner, as confirmed by previous findings [33]. Conversely, in the human myometrium, the thinner bundles appear to be located toward the inner myometrium, and by the same speculative argument one might expect excitation waves to originate in this region.

### Comparison with previous structural analysis

**Fig 43:**
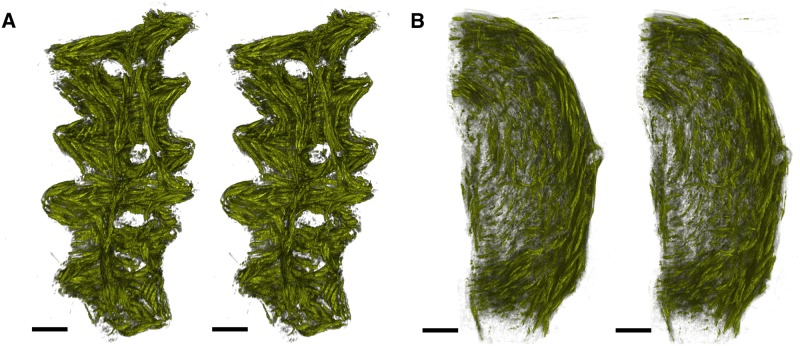
Fibrous structures in rat and human myometrium. Stereo pairs showing the fibrous structure in the rat (**A**) and human (**B**). Images were generated by highlighting portions of tissue along the bundle direction from randomly selected seed points in the reconstruction. S1 Appendix describes the methods used to generate these visualisations. The rat uterus is shown from the exterior with the cervical end at the top. Longitudinal bundles can be seen lying on top, with some distortion due to the pinning of the tissue, while circular bundles are positioned underneath. The human tissue is shown with fundus at the top, interior to the left of the image and exterior to the right. The bundles in the human do not show the same distinctive layers as the rat, with the central portion of the tissue showing no apparent preferred orientation. Toward the interior, circular bundles can be observed as horizontal from this viewing plane, while an inner layer of longitudinal bundles can be observed toward the cervix, in accordance with Weiss *et al.* [3]. The larger structures toward the exterior are in fact sheet-like structures, as previously observed by Young and Hession [2]. Scale bars represent 5 mm.

Overall, the stereoscopic images presented in Fig 43 indicate that the histological micro-architecture accords with Young and Hession’s [2] conception of the cylindric bundle of myocytes as the functional unit of the uterus. Clearly visible are the cylindric structures of 1–2 mm in diameter named *fasciculi* by Goerttler [34]. Moreover, it can be seen that these fasciculi form larger formations. Whereas the rodent myometrial wall can be divided into a more superficial longitudinal layer and a deeper circular layer (Fig 43A), the human situation is more complex, as was previously reported by Weiss *et al.* [3], who observed distinct circular structures near the uterine cavity, in agreement with our reconstruction (Fig 43B), which also shows longitudinal structures deep to the circular layer toward the cervical end of the tissue, again in accordance with Weiss *et al.* [3].

The differences between the organisation of the myometrium in human and the rat may to some extent be attributable to differences in litter size (monotocous versus polytocous) and the concomitant gross morphological differences (simplex versus duplex uterus). In the human, remodelling of the cervix and the generation and control of intra-uterine pressure are the crucial physiological parameters that determine successful parturition, whereas the expulsion of pups in the rodent may require a more coordinated set of movements.

**Fig 44:**
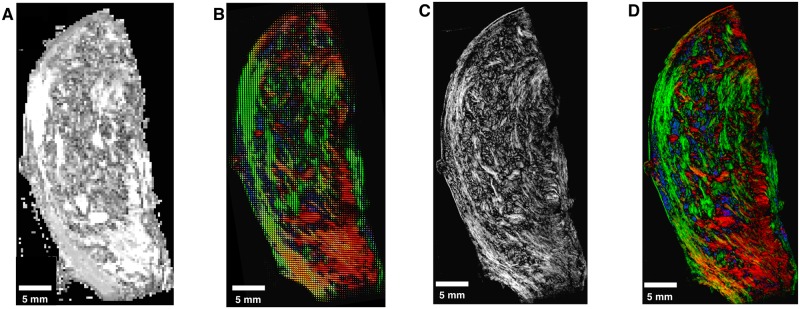
Comparison with anisotropy maps. DT MRI was simulated on the reconstruction to obtain fractional anisotropy (**A**) and diffusion tensor ellipses (**B**) at a resolution of 380 *μ*m per voxel (see S1 Appendix for detailed methods). These can be directly compared to the original weighting (**C**) and directions (**D**), which are at a resolution of 47.5 *μ*m per voxel. The simulated diffusion tensor images are similar to those presented by Weiss *et al.* [3].

To facilitate comparison with previous findings, we simulated DT MRI imaging of the reconstructions at a resolution comparable to that of current DT MRI technology (360 *μ*m per voxel), as shown for the human in Fig 44 (see S1 Appendix for details on how these were generated). The fractional anisotropy map (Fig 44A) shows high levels of anisotropy toward the perimetrium, endometrium, and cervix, with varying levels of anisotropy in the intermediate areas. The diffusion tensor visualisation (Fig 44B) reflects this trend of anisotropy, while the colouring shows that the disorderly structure can be discerned at this coarse-grained level, as previously observed by Weiss *et al.* [3]. A comparison between these coarse-grained images (Fig 44A and 44B) and the original higher-resolution images (Fig 44C and 44D) show that the general bundle direction and structure can be observed at the lower resolutions, although at this resolution (360 *μ*m per voxel) much of the finer microarchitecture is lost. For example, the bundles at the cervical end can be seen as fine bundles forming larger fasciculi in Fig 44D, whereas these distinctions are lost at the lower resolution (Fig 44B). This suggests that a full representation of the myometrial microarchitecture does require representation of at least the resolution presented here (∼50 *μ*m).

**Fig 45:**
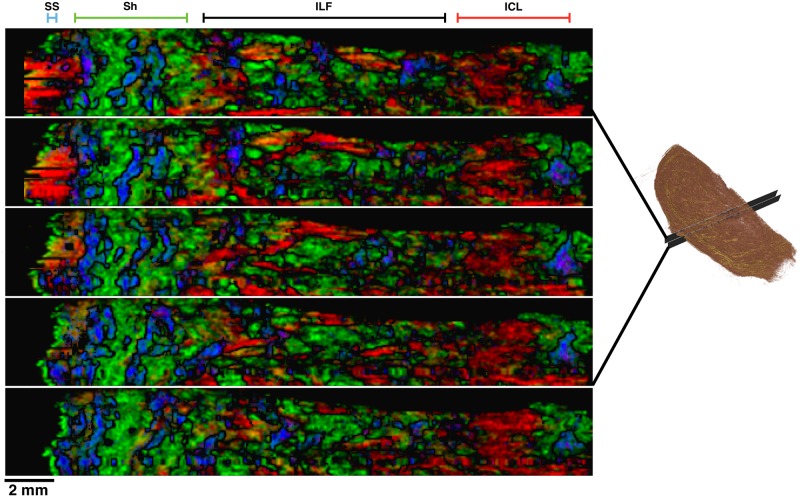
Resectioning of the reconstruction to show layers. Slices shown were taken at 0.475 mm intervals in the range indicated in the right-hand panel, and are displayed with the perimetrium on the left. Blue pixels represent bundles running in the vertical direction in the image plane, red pixels represent horizontal direction in the image plane, and green pixels represent direction perpendicular to the plane. The subserosal sheet (**SS**) can be seen as a line of green at the most superficial end, interrupted in the top images by the Fallopian tube. The sheets present deep to the subserosal sheet (**Sh**) have both through-plane and vertical orientation in the image. The through-plane (green) areas in this layer can be thought of as having been cut transverse to the bundle direction, which indicates that these flat structures are sheets observed in cross-section. The intermediate layer of fasciculi (**ILF**) shows varied bundle direction, as previously observed by Weiss *et al.* [3]. Also in accordance with Weiss *et al.* [3] is the inner circular layer (**ICL**), with a layer of longitudinal bundles deep of this, which is present toward the cervical (caudal) end of the tissue, and appears as areas of blue pixels in the inner circular layer.

**Fig 46:**
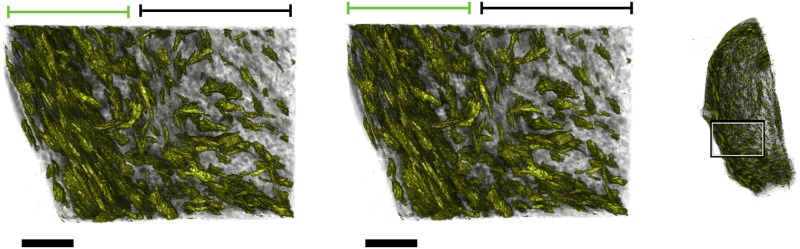
Stereo pair showing sheets. Sheets located just beneath the subserosal sheet are indicated by the green bar, while the deeper layer of smaller fasciculi is indicated by the black bar at the top of the image. The marked difference in structure between the sheets under the green bar and fasciculi under the black bar points to a clear distinction between the two layers. Structures were highlighted at random locations to highlight the large structures shown here (for details, see S1 Appendix). Right-hand panel shows the global position of the images. Scale bars represent 2 mm.

**Fig 47:**
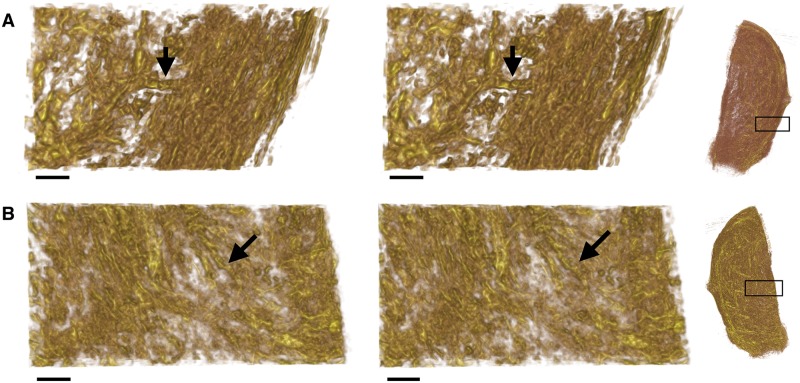
Stereo pairs of bridge-like structures connecting layers of the myometrium. **A**: Bridge connecting a superficial sheet to the intermediate layer of fasciculi. **B**: Bridge connecting the intermediate layer of fasciculi to the circular bundles in the inner myometrium. Panels to the right of the stereo pairs show the global position of the pairs. Scale bars represent 1 mm. These bridges were identified by suppressing voxels of low weight to improve visibility of the structures.

The structural composition of the human myometrium is summarised in Fig 45, in which the *in silico* representation has been resectioned to give virtual cross-sections perpendicular to the long axis of the organ. The most superficial part of the tissue is occupied by the subserosal sheet (∼200 *μ*m) [2]. Deep to this feature is a layer of sheets, as previously observed by Young and Hession [2], which is approximately 5 mm thick. These sheets are visualised in Fig 46, where it can be observed that there is a boundary where these sheets dissolve into a system of smaller fasciculi. The sheets are connected to the smaller fasciculi deep to the sheets by bridge-like structures, as shown in Fig 47A. The layer of smaller fasciculi extends from the layer of sheets to the inner circular layer (Fig 45). Again, connections between the intermediate layer of fasciculi and the circular bundles can be observed, as is shown in Fig 47B. The circular layer approaches the interior cavity of the uterus, except toward the cervical (caudal) end of the uterus, where there is an additional layer of longitudinal bundles, as previously observed by Weiss *et al.* [3]. Fig 44 shows that this inner longitudinal layer does not extend to the fundal portion of the uterine cavity.

**Fig 48:**
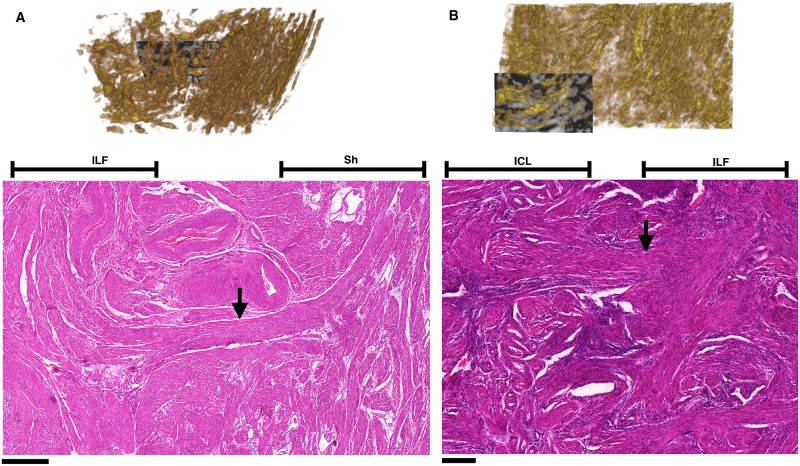
Histological images of identified bridge-like structures. **A**: Bridge connecting a superficial sheet (**Sh**) to the intermediate layer of fasciculi (**ILF**). Top panel shows the position of the slice in the reconstruction, scale bar indicates 500 *μ*m. **B**: Bridge connecting the intermediate layer of fasciculi (**ILF**) to the circular bundles in the inner myometrium (**ICL**). Top panel shows the position of the slice in the reconstruction, scale bar indicates 200 *μ*m.

The reconstructions generated here are in direct correspondence with the original histological images. This means that any structures found in the reconstructed tissue can be referred back to the histological slides that contain these structures. This enables direct verification of the presence of these structures, and allows one to glean contextual information not present in the reconstruction. Fig 48 shows this histological context for the bridge-like structures shown in Fig 47. The large bridge-like structure connecting the sheet layer to the intermediate layer of fasciculi is clearly visible in Fig 48A, confirming the existence of the structure. Similarly, the point of connection between the intermediate layer of fasciculi and the inner circular layer is shown in Fig 48B.

**Fig 49:**
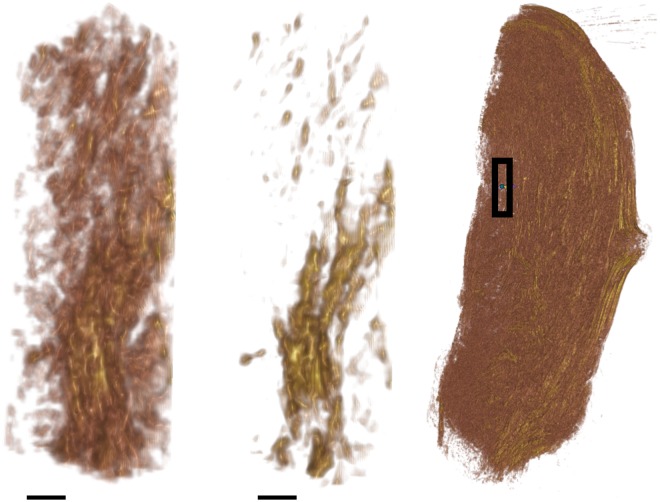
Resolving substructures within a single fasciculus. The left-hand panel shows a reconstructed fasciculus where voxel opacity represents the assigned weights, which themselves represent where the voxels are positioned in a fibrous structure, with high weights representing points in the centre of a bundle, while low weights represent edges of the bundles. The middle panel shows the same structure with the lower weights suppressed, revealing smaller fibrous components of the fasciculus, which correspond to the bundles previously classified by Young and Hession [2]. Right hand panel shows the global location of the fasciculus. Scale bar represents 0.5 mm.

The fasciculi are composed of several smaller structures termed *bundles* by Young and Hession [2], each consisting of functional contractile units of myocytes that adhere closely and are similarly oriented. Even though the acquisition step in our algorithm resolves individual cells (width 5–10 *μ*m), the spatial averaging required to obtain reliable bundle directions results in an effective spatial resolution of about 50 *μ*m (edge width of one voxel), which is still substantially below the 1 mm width cited for fasciculi. The classical fasciculi are visible to the unaided eye and can be dissected free [2]; our method resolves these structures nearly to the level of the individual bundle. An example of the finer structure of a fasciculus is shown in Fig 49, where the isolation of individual bundles was achieved by suppressing voxels with low weighting, which corresponds to points at the edge of a fibrous structure. The finer fibrous structures contained within the fasciculus shown in Fig 49 represent, in all likelihood, the bundles previously observed by Young and Hession [2]. Similar examples can be observed in two dimensions in Figs 44 and 45, where the variation in intensity emphasizes individual bundles.

**Fig 50:**
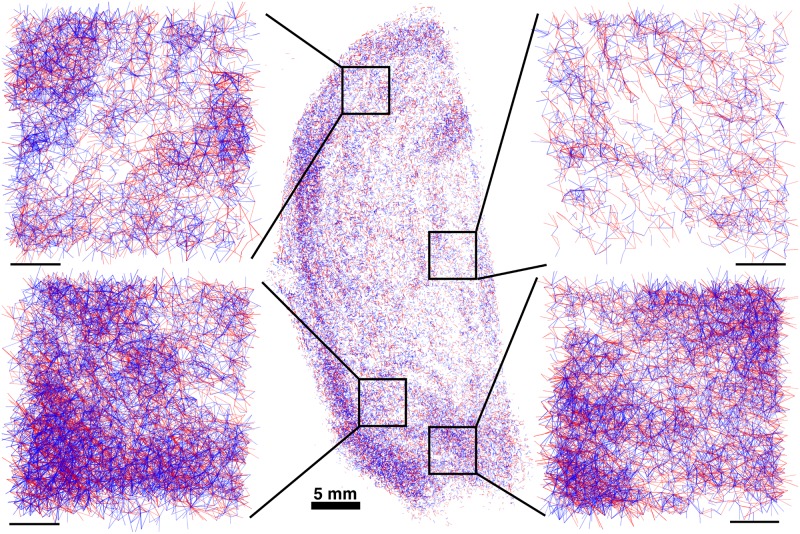
Interconnected network of bundles. This alternative representation of the reconstruction shows connections between bundles as arcs in a graph (details can be found in S1 Appendix). The red arcs represent the bundles, while blue arcs represent connections between bundles. The central panel shows a random selection of 10% of the arcs from the full representation. Close-up views show the network with connections of low weighting (which is derived from the structural weighting) removed to improve visibility. Larger structures, such as fasciculi (top left, bottom right) and sheets (bottom left, upper left corner of top left) can be identified by the high density of connections between bundles (blue arcs). In contrast, the fibrous structures toward the circular layer (top right) are smaller, and hence fewer connections can be observed between bundles. Scale bars in the close-up networks represent 1 mm.

Young and Hession [2] surmised that myometrial fibres form a network of communicating sheets and merging cylindrical structures and proposed that these structures merge to form a network that connects the majority of myocytes in a system of contiguous pathways. The present method not only allows this network to be resolved at the 50 *μ*m level, but also allows the reconstruction of the contiguous network at the whole-organ level, as shown in Fig 50.

### Limitations

The accurate identification of boundaries between structures constitutes a critical issue, which is partially mitigated by the use of nuclear density weighting (**Phase VI**); whereby a voxel positioned on the boundary of two bundles receives a lower weighting since it has a lower nuclear density than one positioned within a bundle. In addition, the segmentation method as applied during **Phase VII** enables the identification of boundaries between two bundles of different direction. However, adjacent parallel bundles cannot be identified in this manner. One possible solution to this problem would be to apply a collagen stain, such as Masson’s trichrome, which would highlight the boundaries of the bundles, as used previously by Young and Hession [2]. An alternative solution would be to increase the spatial resolution of the reconstruction; however, the current implementation requires multiple nuclei to be present in each voxel in order to obtain a reliable bundle direction, and a substantial increase in resolution (> 4×) would force each voxel to contain at most one nucleus.

While the reconstructions generated in this paper have been verified with respect to the two-dimensional bundle structure and accuracy of registration was visually confirmed, the inferred three-dimensional microarchitecture cannot be directly compared to the original tissue. Confirmation of the accuracy of the three-dimensional microarchitecture could be obtained by using an equivalent three-dimensional imaging technique, such as high-powered MRI [5], or micro-CT [6].

The human tissue sample examined here did not encompass the entire organ, and therefore the observations made here are limited to what was present in the sample. For example, the dorsal and caudal regions were not examined here and might have a layer of sheets that extends deeper into the myometrium than observed in this sample (5 mm). The structural features observed here could be investigated at the whole-organ level by performing the reconstruction procedure on a complete uterus; however, practical factors such as a limit on the size of slides available for use and processing time could hamper such a direct approach. An alternative approach would be to reconstruct specific portions of uterus, such as the fundus or cervix.

All slides in the tissue blocks were subjected to small distortions during the sectioning of the tissue. The registration algorithm (**Phase III**) effectively registers all slides elastically to one global reference slide, which means that the final reconstruction is subject to the small distortions present in this global reference slide. The algorithm selected a global reference slide in a manner which aims to minimise this distortion, but it is inevitably present and difficult to quantify. One possible solution to this problem would be to perform staining and image capture on one or more slides prior to sectioning, as has previously been performed [7]. This hybrid approach would prevent the initial distortion of the global reference slide, and could also be used to prevent incremental distortion on larger tissue blocks, while minimising the time-consuming process of staining and scanning each slide prior to sectioning.

### Future research

The methods described here use nuclear shape and orientation to determine the three-dimensional structure of myometrial smooth muscle. The reliable and consistent relationship between nuclei and fibre direction utilised here is also present in other smooth muscle tissues, which suggests that these methods could be readily applied to the reconstruction of such tissues. One potential use for this method is the visualisation of fibrosis in the heart. This structural formation is not readily examined with MRI or CT techniques, but could be examined using the technique presented here. Other potential applications of this technique include the investigation of atherosclerosis in vascular tissue and visualising the fine structure of other visceral smooth muscle tissues such as those in the gastro-intestinal tract. Inasmuch as the registration technique relies on heterogeneity of the fibrous structure of the tissue, the registration method could be less well-suited to more regular or periodic structures, such as skeletal muscle.

A key advantage of this reconstruction technique over other three-dimensional imaging techniques is the direct correspondence to histological slides. This correspondence could be further utilised in future implementations by applying additional stains to the slides, either as a counterstain to the H & E stains used here, or applied by restaining the tissue after the initial scanning of the slides. This would enable further contextualisation for any structural features discovered in the reconstruction. For example, immunofluorescent staining for connexin proteins would highlight gap junction densities and provide information on the conductance of the tissue.

## Conclusion

The methods presented here enable the reconstruction of myometrial smooth muscle in rat and human uteri from serial histological sections at a resolution of ∼50 *μ*m per voxel length. This reconstruction has been shown to be faithful to the original histological sections and the general habitus of the original tissue. The spatial distribution of bundle widths in the reconstructions was presented, showing an increase in width from interior to exterior in the rat, while in the human the widths decreased from interior to exterior. The *in silico* reconstruction of the tissue can be interrogated via a range of visualisation tools, allowing researchers to interpret the spatial data set according to their needs. The reconstructions are in direct correspondence with the original histological slides taken from the tissue, which allows the inspection of anatomical features identified in the three-dimensional reconstruction in the context of the original tissue. The techniques used are based on myometrial smooth muscle architecture, but can be applied to other smooth muscle tissue types.

## Supporting information

S1 Fig Histological image of a randomly selected area of human myometrium. Image dimensions: 760 *μ*m × 760 *μ*m, stained with haemotoxylin and eosin.
(TIFF)

S2 Fig Comparison of assigned direction vectors with the original histological image S1 Fig. Image dimensions: 760 *μ*m × 760 *μ*m, stained with haemotoxylin and eosin.
(TIFF)

S3 Fig Histological image of a randomly selected area of human myometrium. Image dimensions: 760 *μ*m × 760 *μ*m, stained with haemotoxylin and eosin.
(TIFF)

S4 Fig Comparison of assigned direction vectors with the original histological image S3 Fig. Image dimensions: 760 *μ*m × 760 *μ*m, stained with haemotoxylin and eosin.
(TIFF)

S5 Fig Histological image of a randomly selected area of rat myometrium. Image dimensions: 760 *μ*m × 760 *μ*m, stained with haemotoxylin and eosin.
(TIFF)

S6 Fig Comparison of assigned direction vectors with the original histological image S5 Fig. Image dimensions: 760 *μ*m × 760 *μ*m, stained with haemotoxylin and eosin.
(TIFF)

S7 Fig Histological image of a randomly selected area of rat myometrium. Image dimensions: 760 *μ*m × 760 *μ*m, stained with haemotoxylin and eosin.
(TIFF)

S8 Fig Comparison of assigned direction vectors with the original histological image S7 Fig. Image dimensions: 760 *μ*m × 760 *μ*m, stained with haemotoxylin and eosin.
(TIFF)

S9 Fig Histological image of a randomly selected area of rat myometrium. Image dimensions: 950 *μ*m × 950 *μ*m, stained with haemotoxylin and eosin.
(TIFF)

S10 Fig Comparison of assigned direction vectors with the original histological image S9 Fig. Image dimensions: 950 *μ*m × 950 *μ*m, stained with haemotoxylin and eosin.
(TIFF)

S11 Fig Histological image of a randomly selected area of rat myometrium. Image dimensions: 950 *μ*m × 950 *μ*m, stained with haemotoxylin and eosin.
(TIFF)

S12 Fig Comparison of assigned direction vectors with the original histological image S11 Fig. Image dimensions: 950 *μ*m × 950 *μ*m, stained with haemotoxylin and eosin.
(TIFF)

S13 Fig Histological image of a randomly selected area of rat myometrium. Image dimensions: 950 *μ*m × 760 *μ*m, stained with haemotoxylin and eosin.
(TIFF)

S14 Fig Comparison of assigned direction vectors with the original histological image S13 Fig. Image dimensions: 760 *μ*m × 760 *μ*m, stained with haemotoxylin and eosin.
(TIFF)

S15 Fig Histological image of a randomly selected area of rat myometrium. Image dimensions: 760 *μ*m × 760 *μ*m, stained with haemotoxylin and eosin.
(TIFF)

S16 Fig Comparison of assigned direction vectors with the original histological image S15 Fig. Image dimensions: 760 *μ*m × 760 *μ*m, stained with haemotoxylin and eosin.
(TIFF)

S1 File Code used in reconstruction. Includes all reconstruction code and code for exporting visualisations in dicom format. All reconstruction and visualisation code is written in Java and is packaged as a set of ImageJ plugins.
(SH) Click here for additional data file.

S2 File Slide conversion shell script. Converts slides from the Mirax proprietary format into tiff files that can be accessed in ImageJ.
(ZIP)

S1 Appendix Additional methods for visualisation of fibrous structures. (PDF)
