# Supplementary figures and images for: A computational method for three-dimensional reconstruction of the microarchitecture of myometrial smooth muscle from histological sections
Source: PLoS One. 2017 Mar 16;12(3):e0173404. doi: 10.1371/journal.pone.0173404 (PMC5354307; doi:10.1371/journal.pone.0173404)

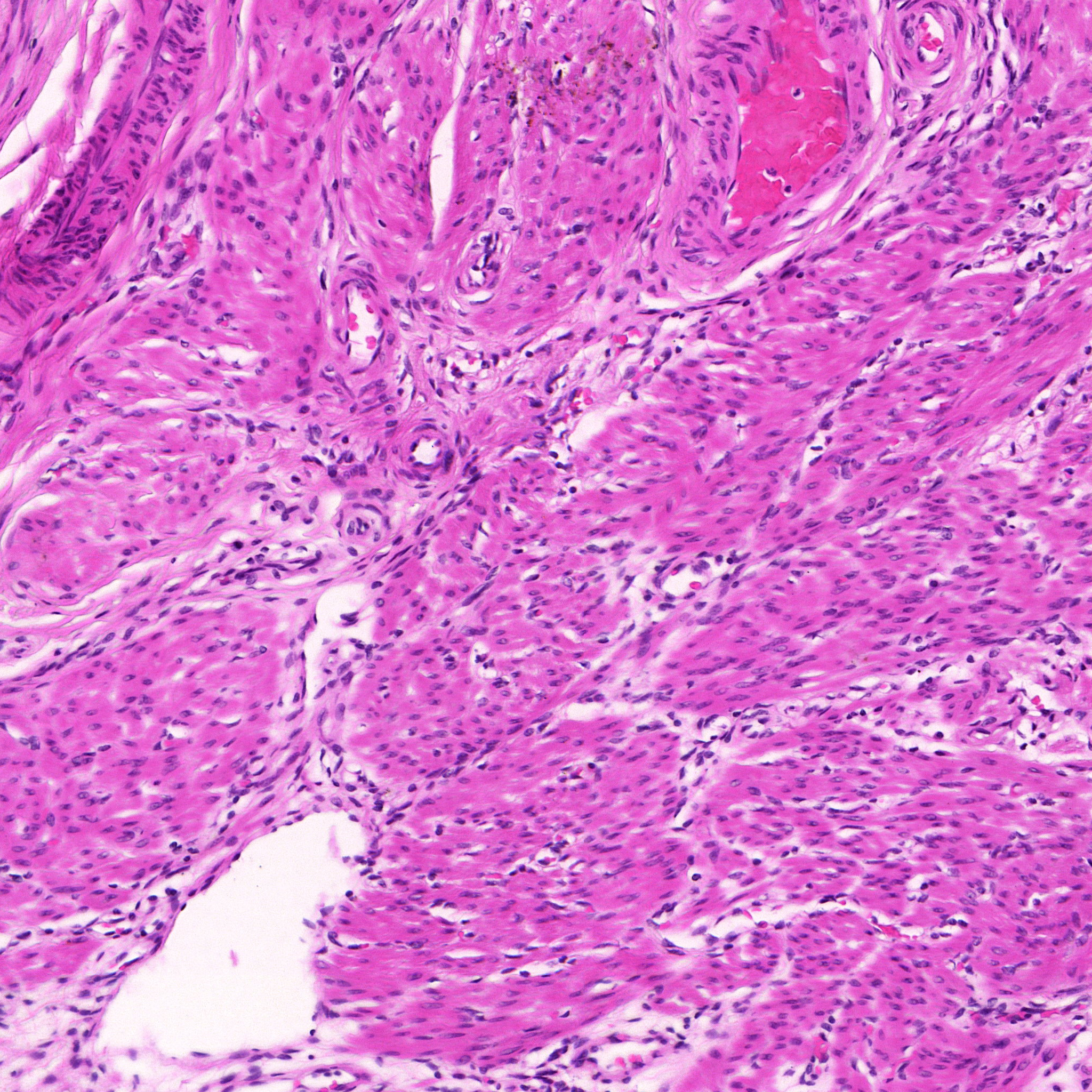

Supplement: S1 Fig — Image dimensions: 760 μm × 760 μm, stained with haemotoxylin and eosin. (TIFF) [file pone.0173404.s001.tiff]

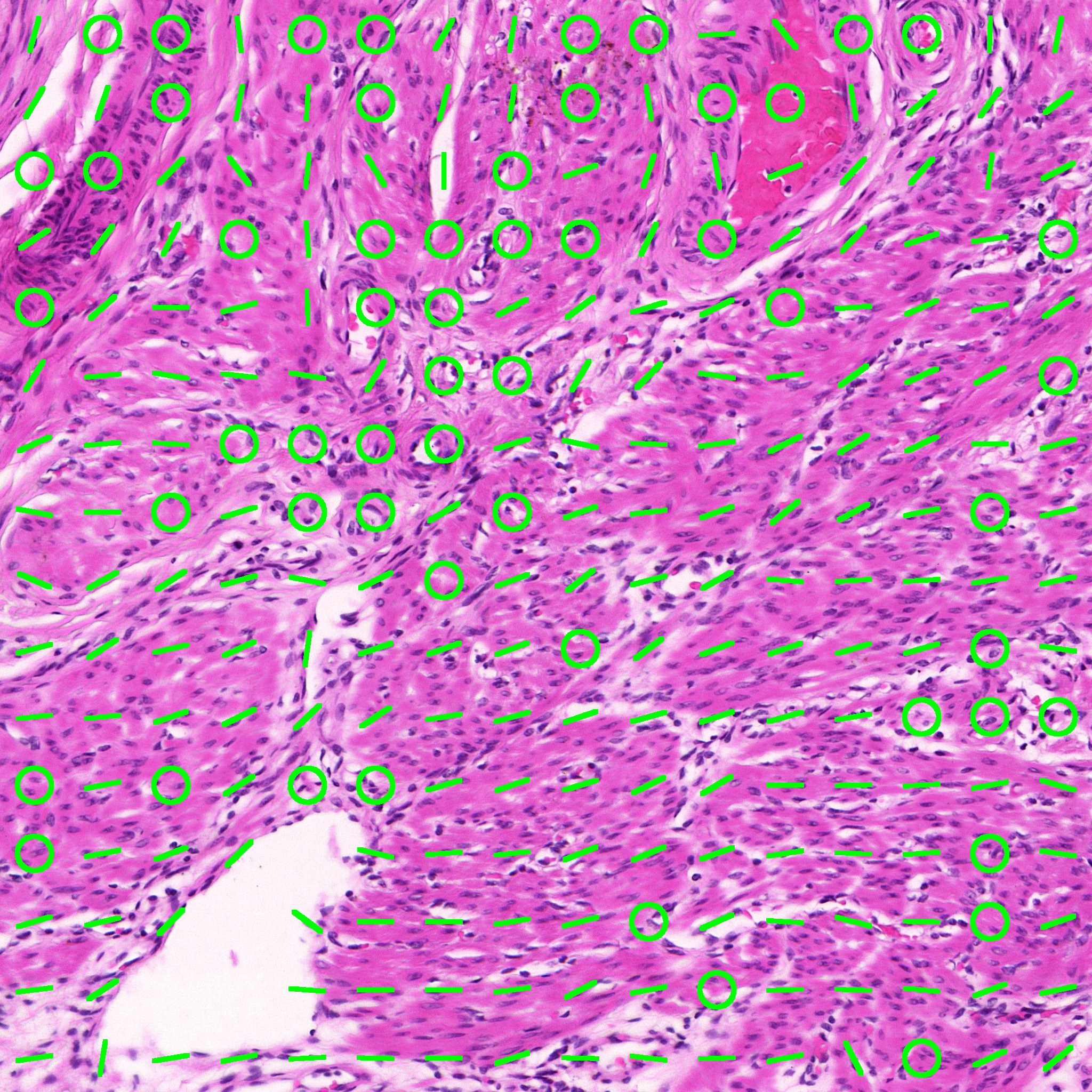

Supplement: S2 Fig — Image dimensions: 760 μm × 760 μm, stained with haemotoxylin and eosin. (TIFF) [file pone.0173404.s002.tiff]

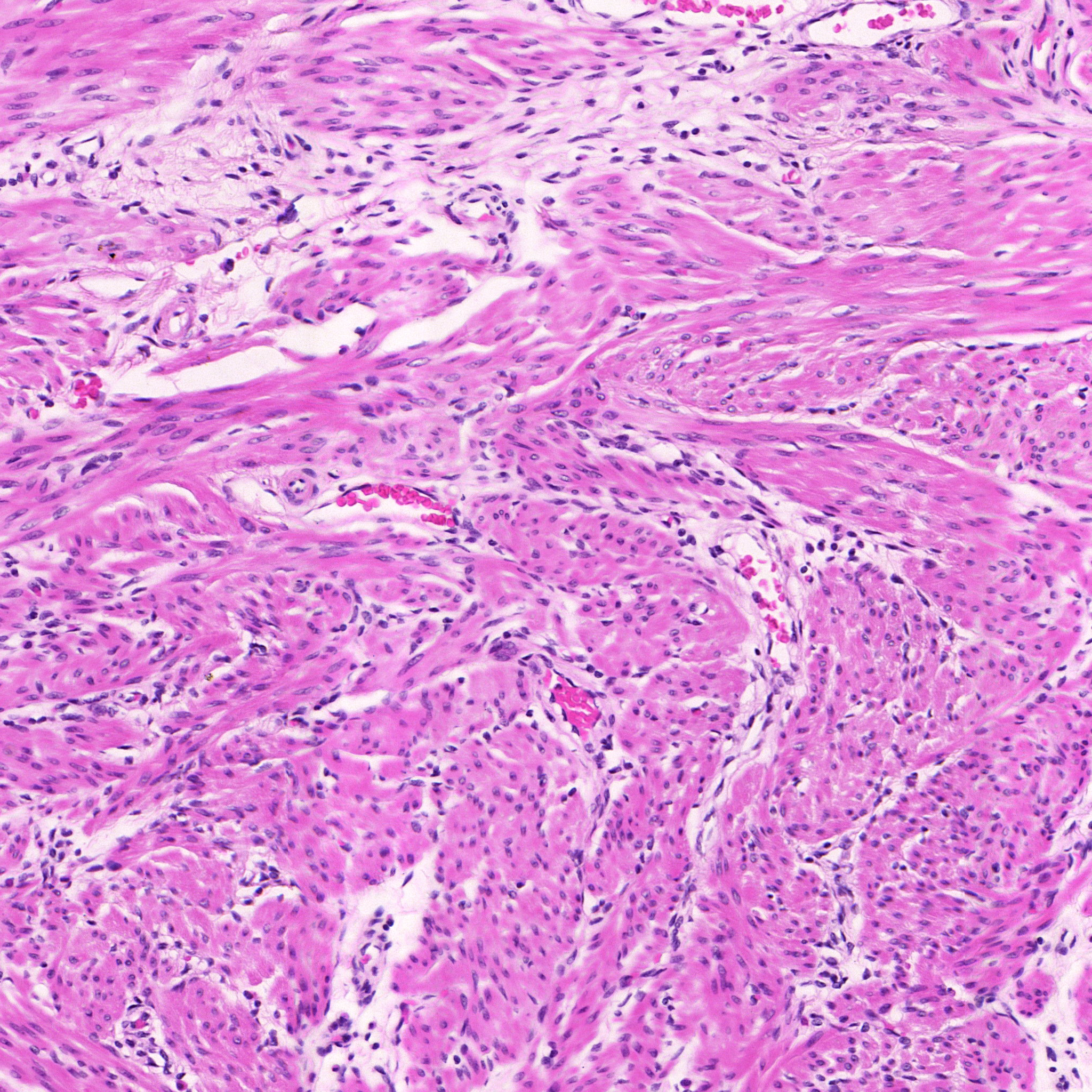

Supplement: S3 Fig — Image dimensions: 760 μm × 760 μm, stained with haemotoxylin and eosin. (TIFF) [file pone.0173404.s003.tiff]

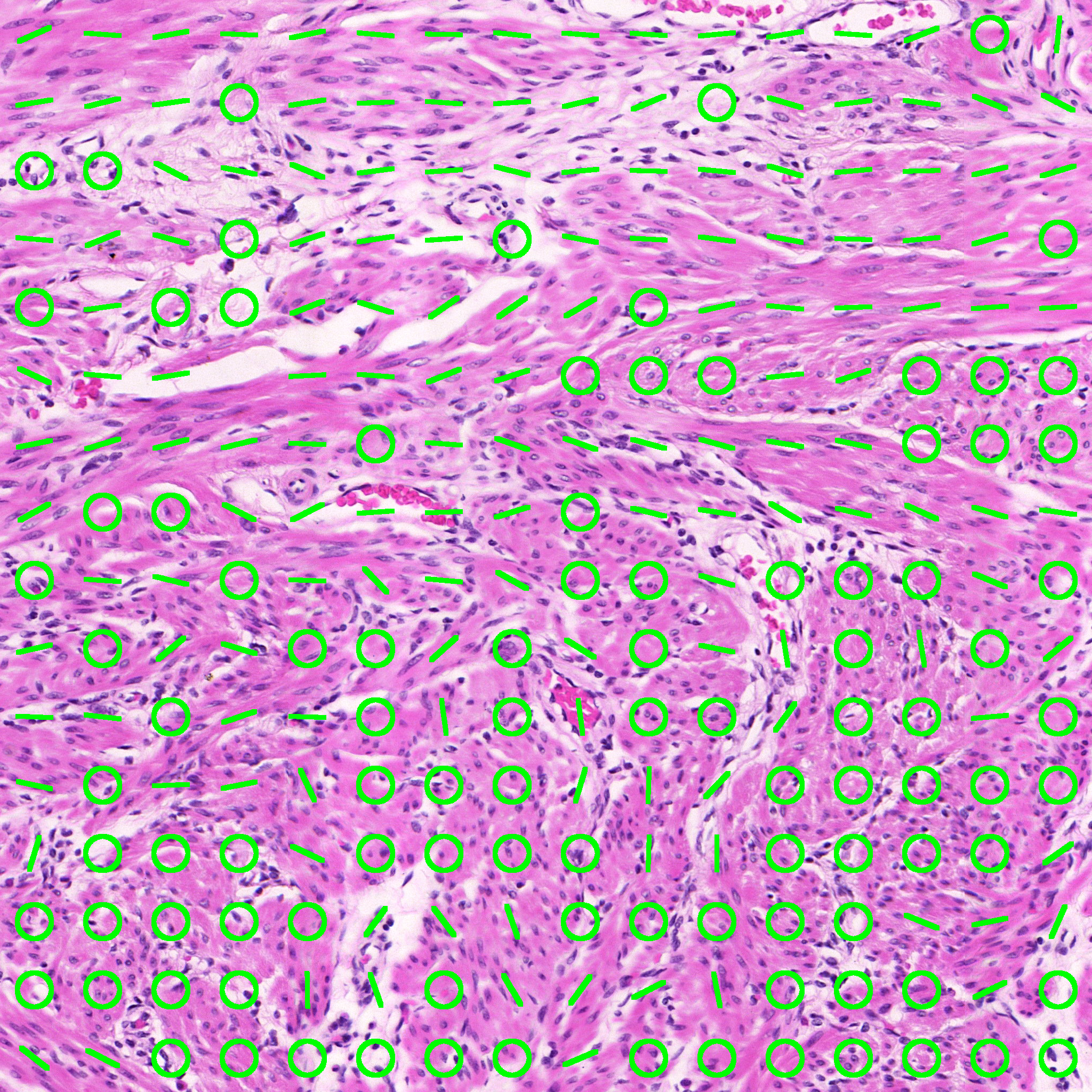

Supplement: S4 Fig — Image dimensions: 760 μm × 760 μm, stained with haemotoxylin and eosin. (TIFF) [file pone.0173404.s004.tiff]

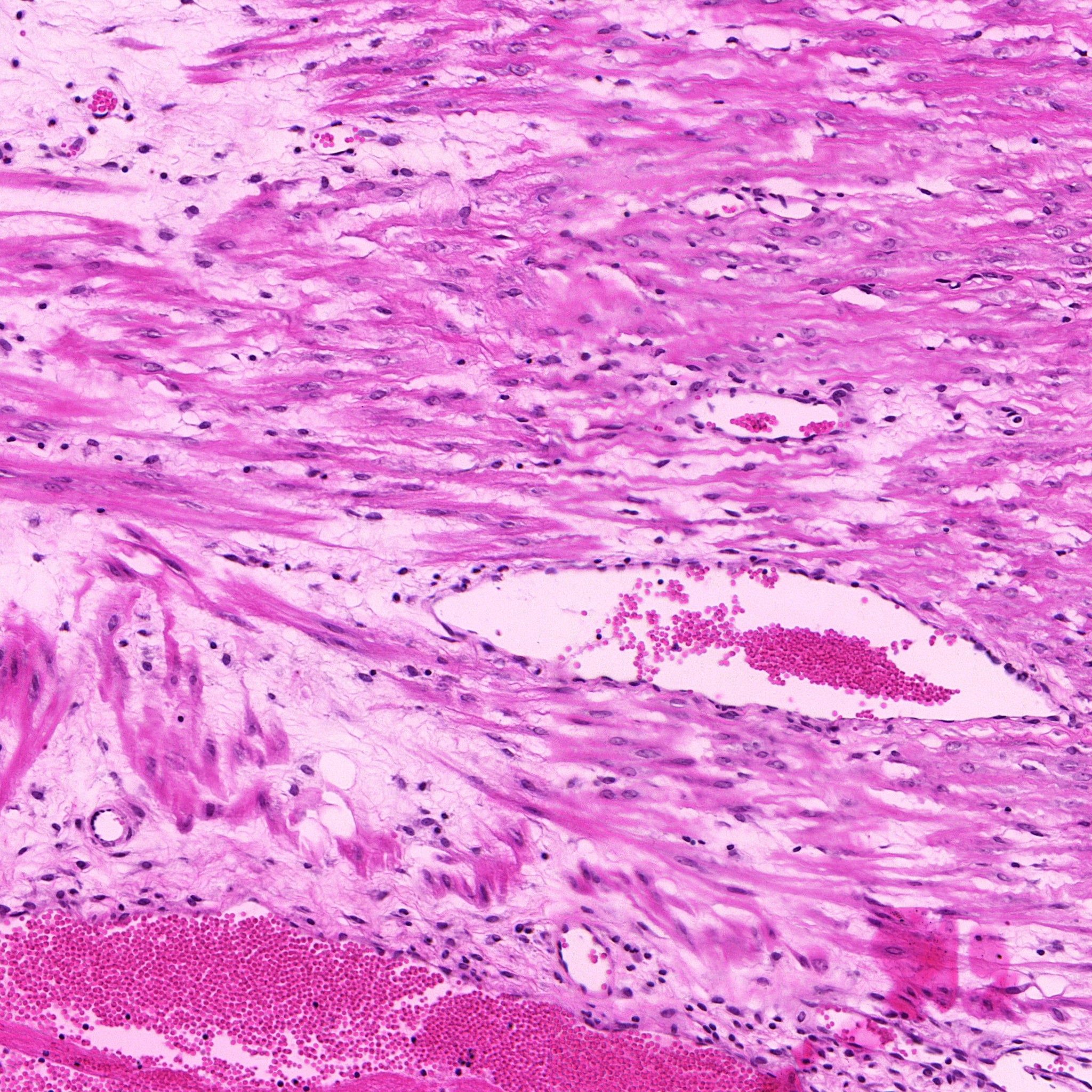

Supplement: S5 Fig — Image dimensions: 760 μm × 760 μm, stained with haemotoxylin and eosin. (TIFF) [file pone.0173404.s005.tiff]

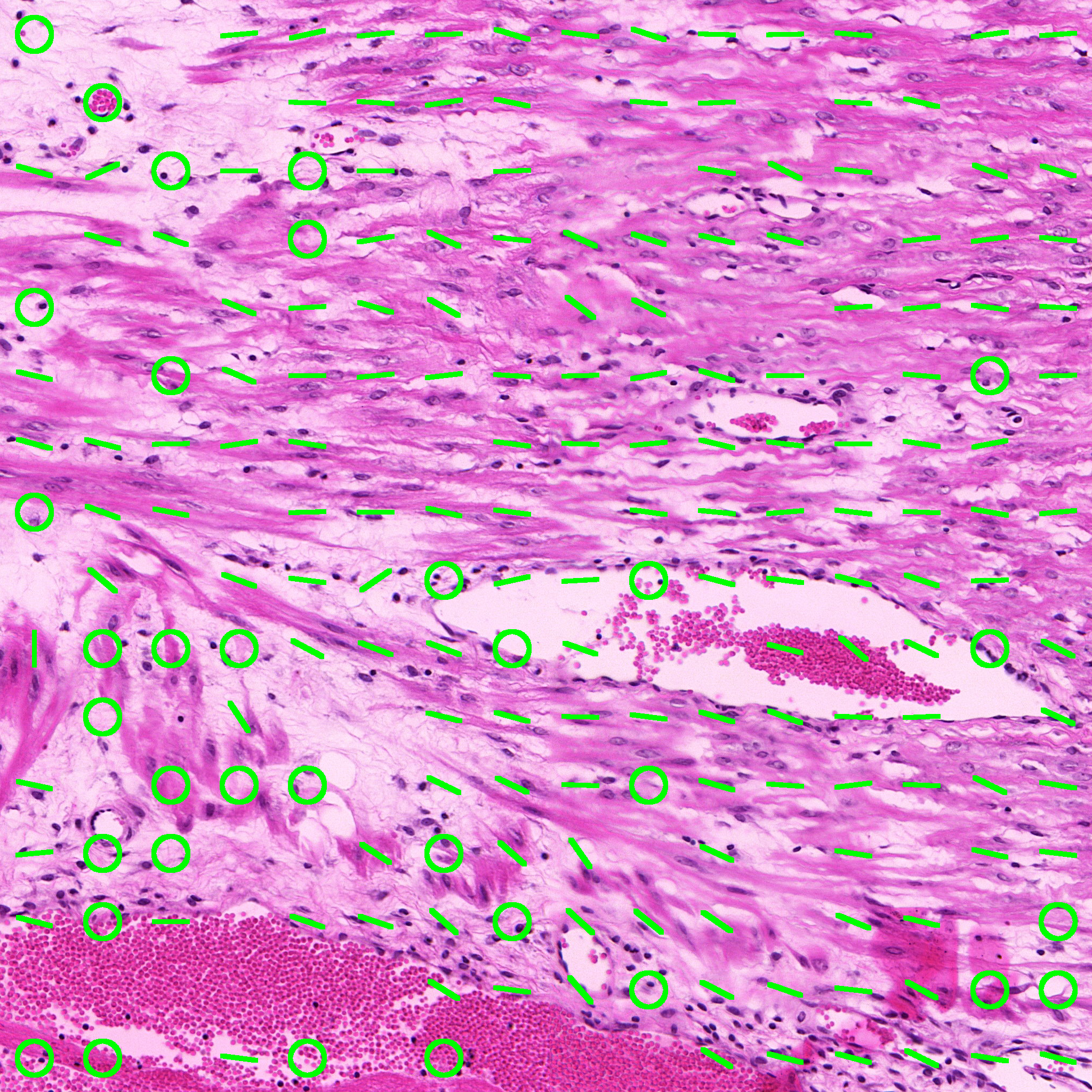

Supplement: S6 Fig — Image dimensions: 760 μm × 760 μm, stained with haemotoxylin and eosin. (TIFF) [file pone.0173404.s006.tiff]

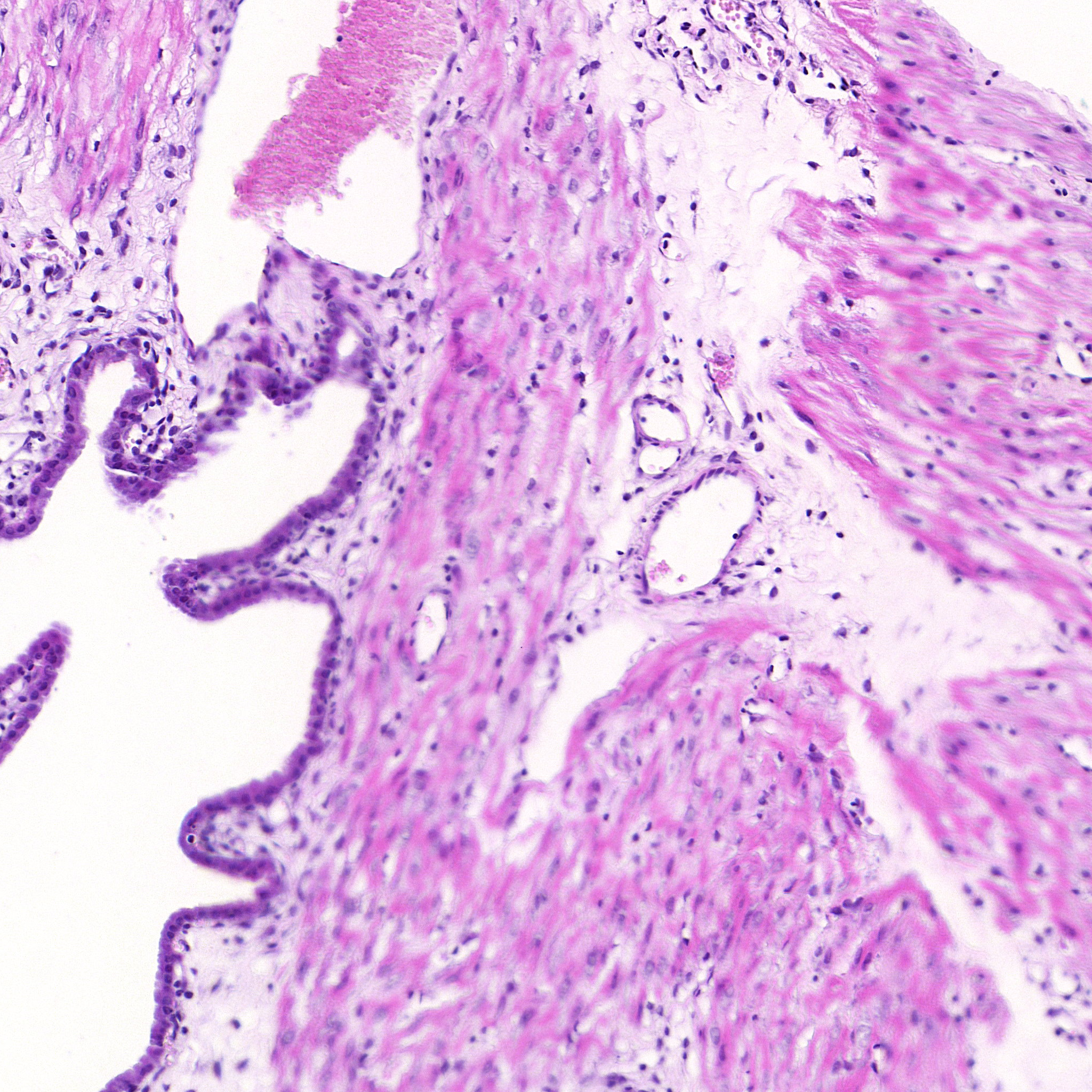

Supplement: S7 Fig — Image dimensions: 760 μm × 760 μm, stained with haemotoxylin and eosin. (TIFF) [file pone.0173404.s007.tiff]

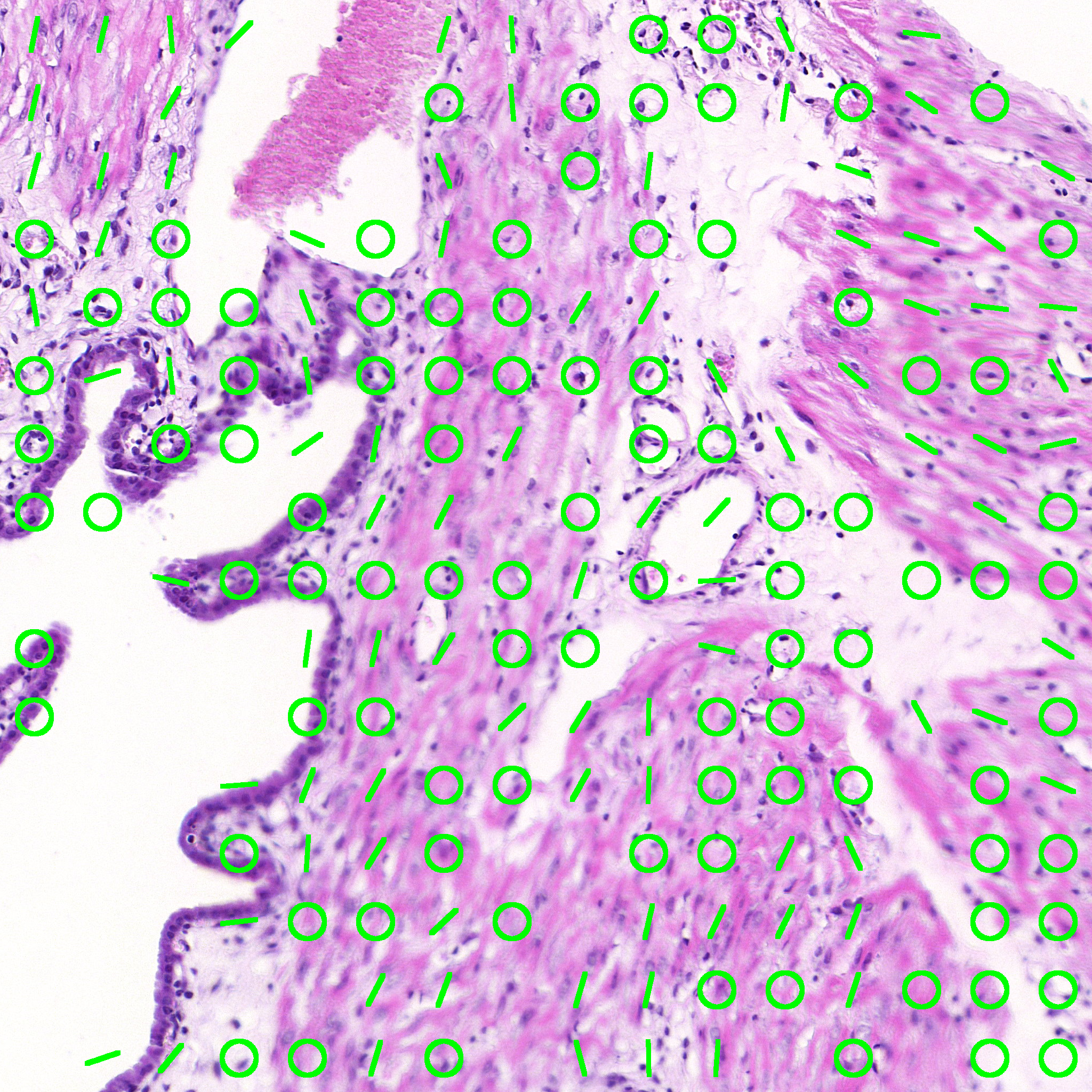

Supplement: S8 Fig — Image dimensions: 760 μm × 760 μm, stained with haemotoxylin and eosin. (TIFF) [file pone.0173404.s008.tiff]

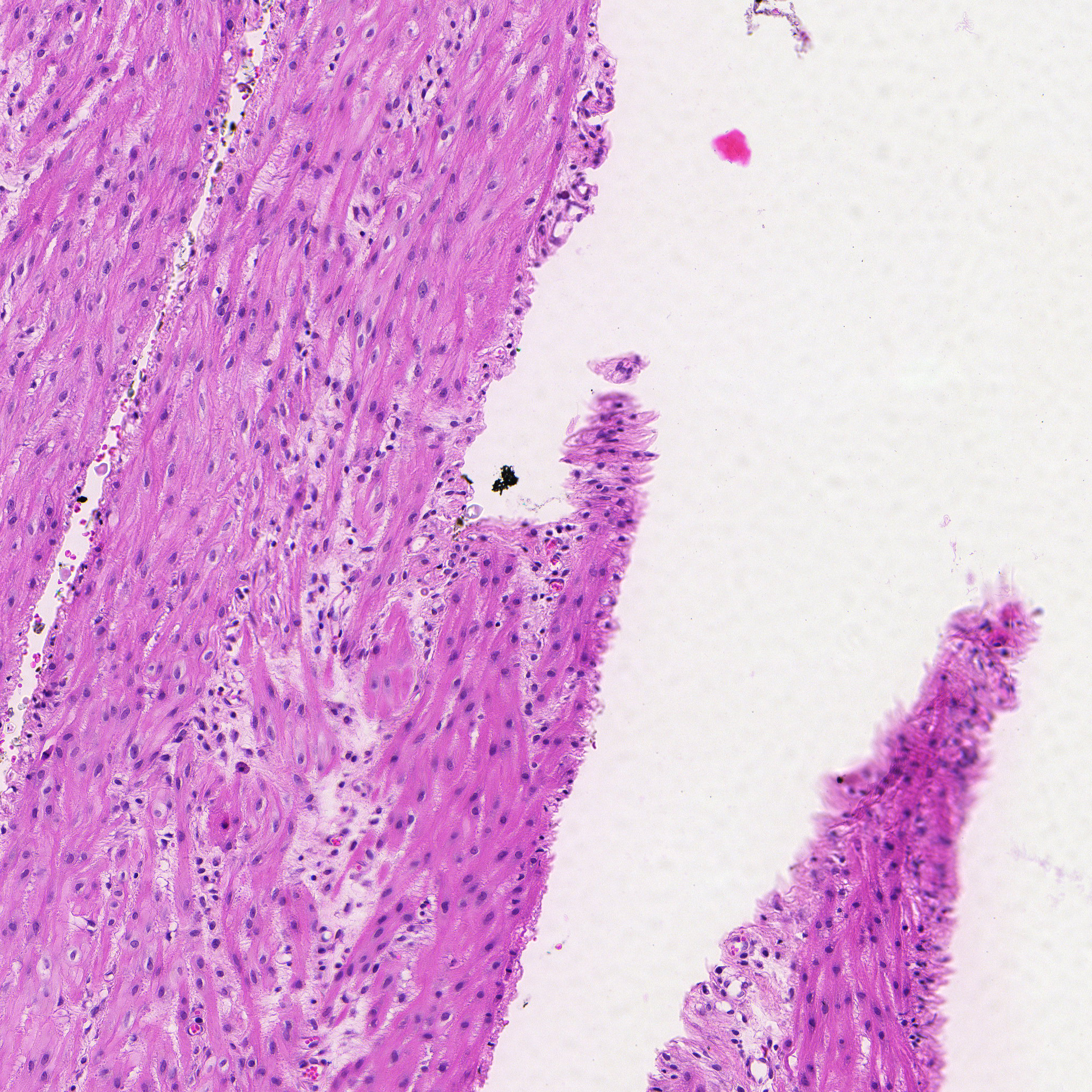

Supplement: S9 Fig — Image dimensions: 950 μm × 950 μm, stained with haemotoxylin and eosin. (TIFF) [file pone.0173404.s009.tiff]

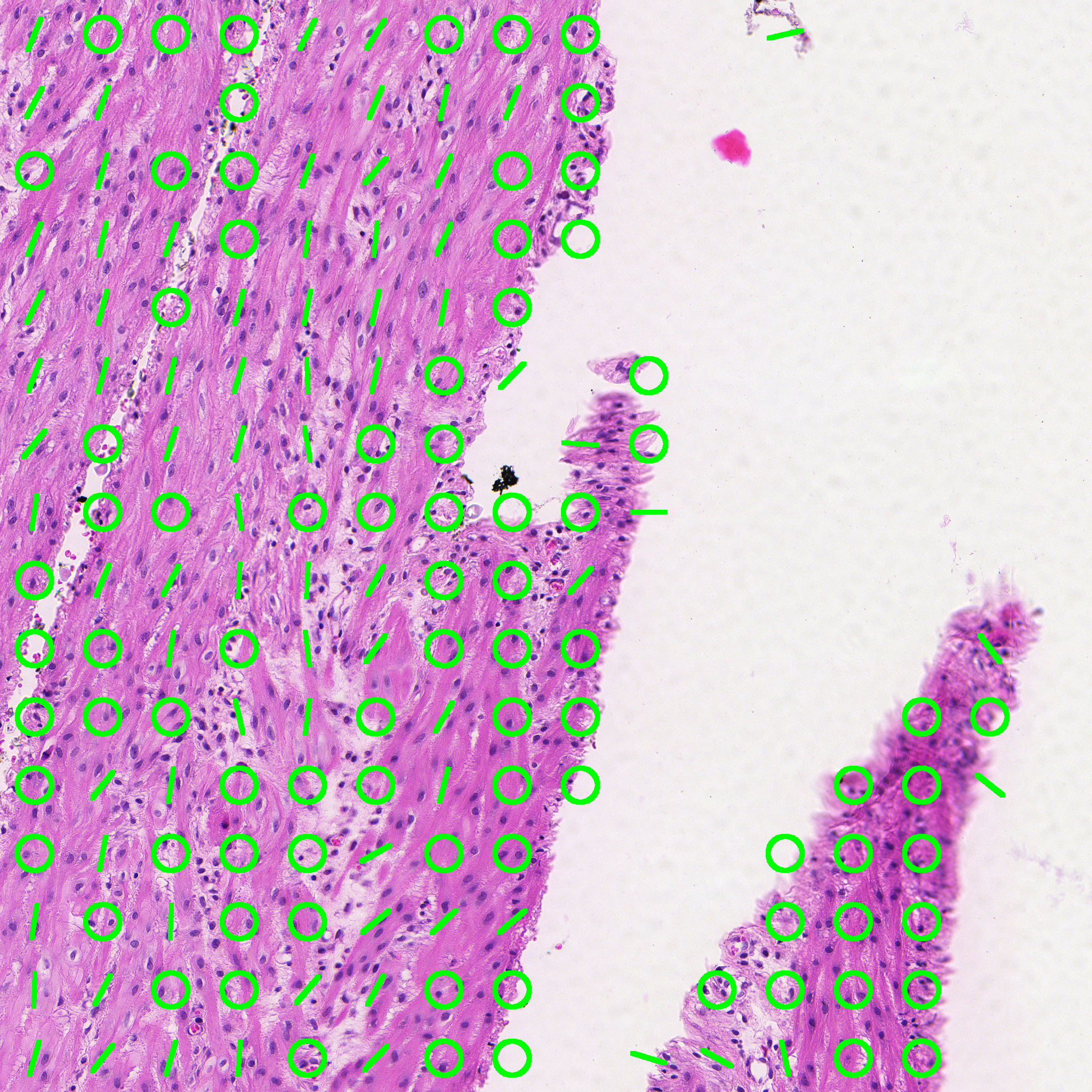

Supplement: S10 Fig — Image dimensions: 950 μm × 950 μm, stained with haemotoxylin and eosin. (TIFF) [file pone.0173404.s010.tiff]

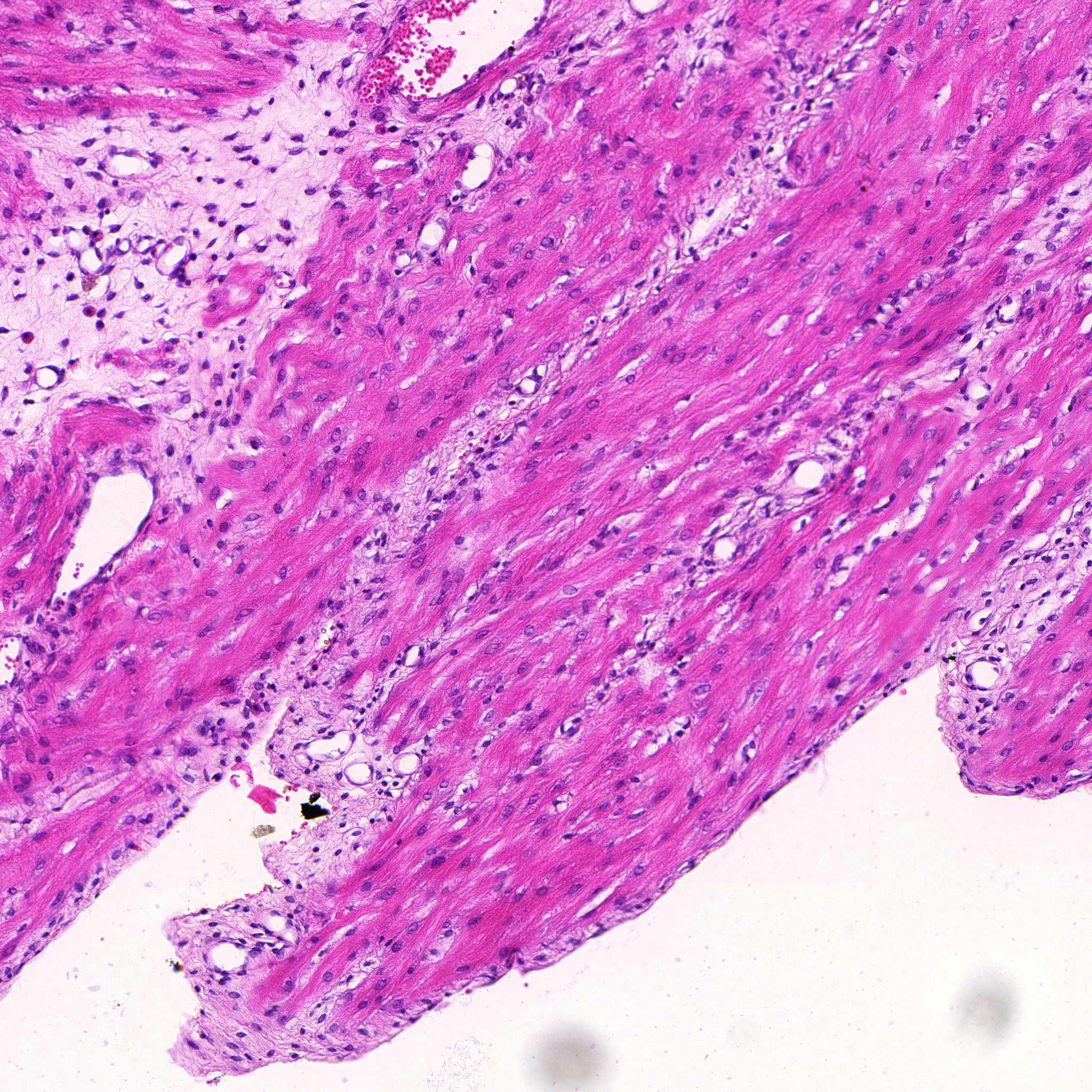

Supplement: S11 Fig — Image dimensions: 950 μm × 950 μm, stained with haemotoxylin and eosin. (TIFF) [file pone.0173404.s011.tiff]

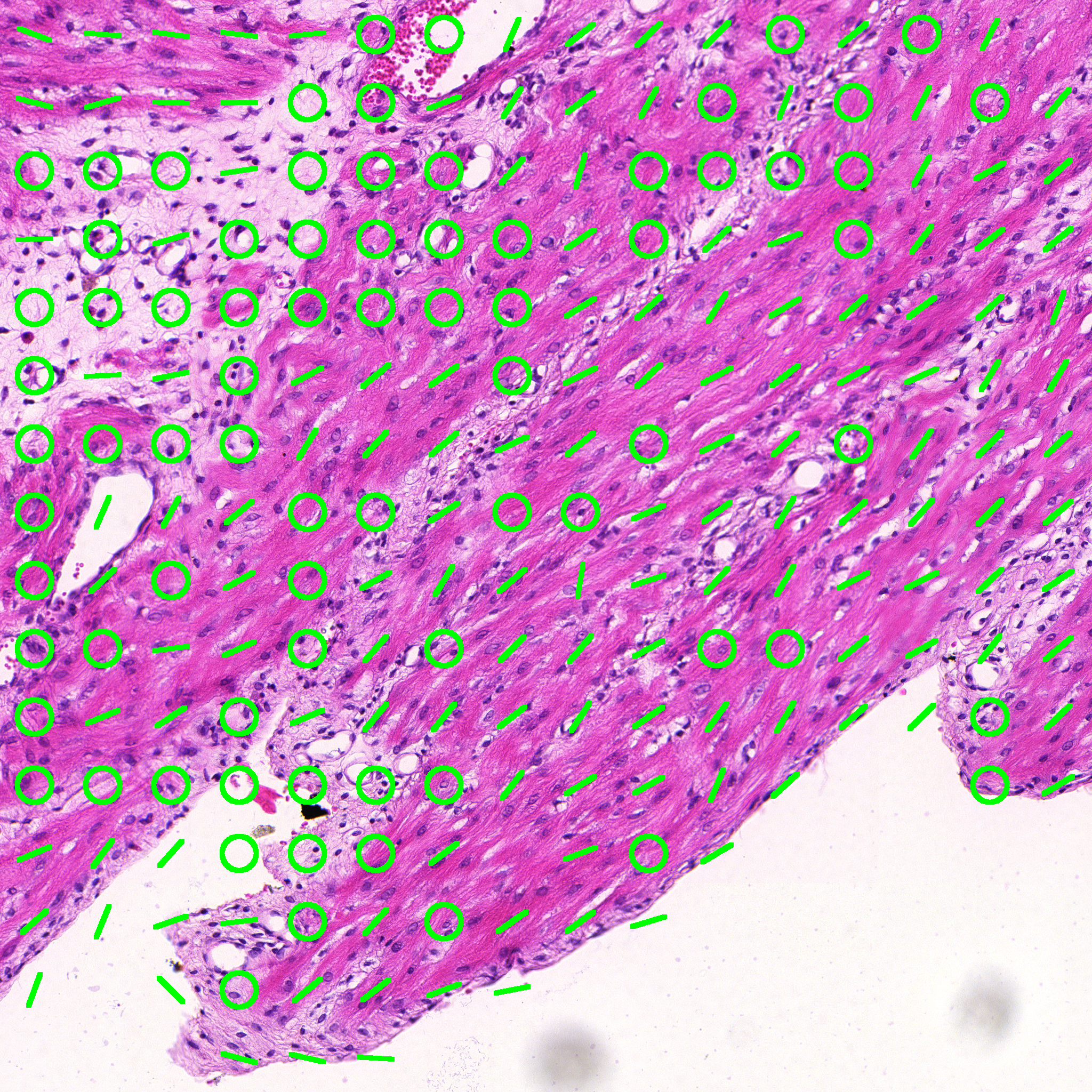

Supplement: S12 Fig — Image dimensions: 950 μm × 950 μm, stained with haemotoxylin and eosin. (TIFF) [file pone.0173404.s012.tiff]

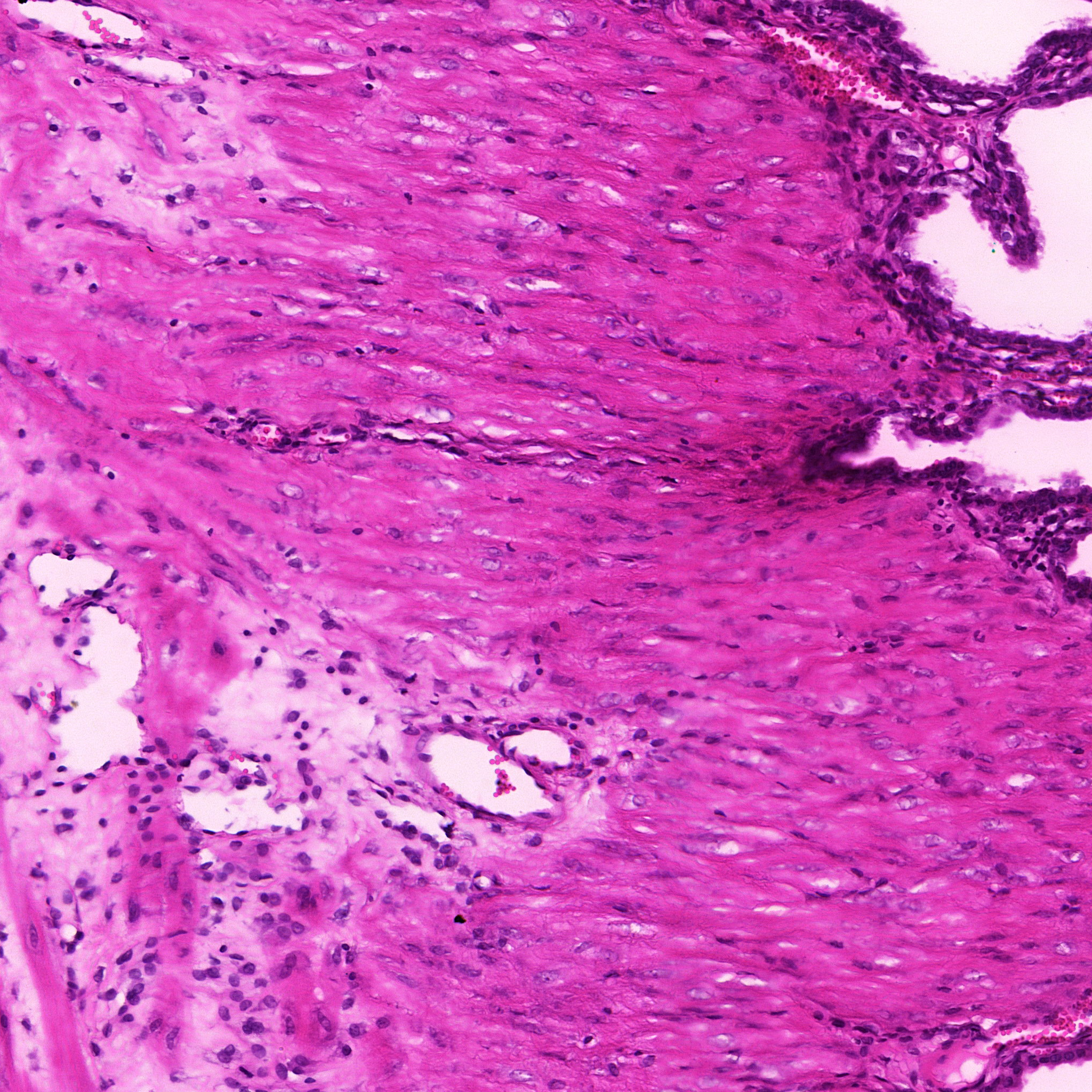

Supplement: S13 Fig — Image dimensions: 950 μm × 760 μm, stained with haemotoxylin and eosin. (TIFF) [file pone.0173404.s013.tiff]

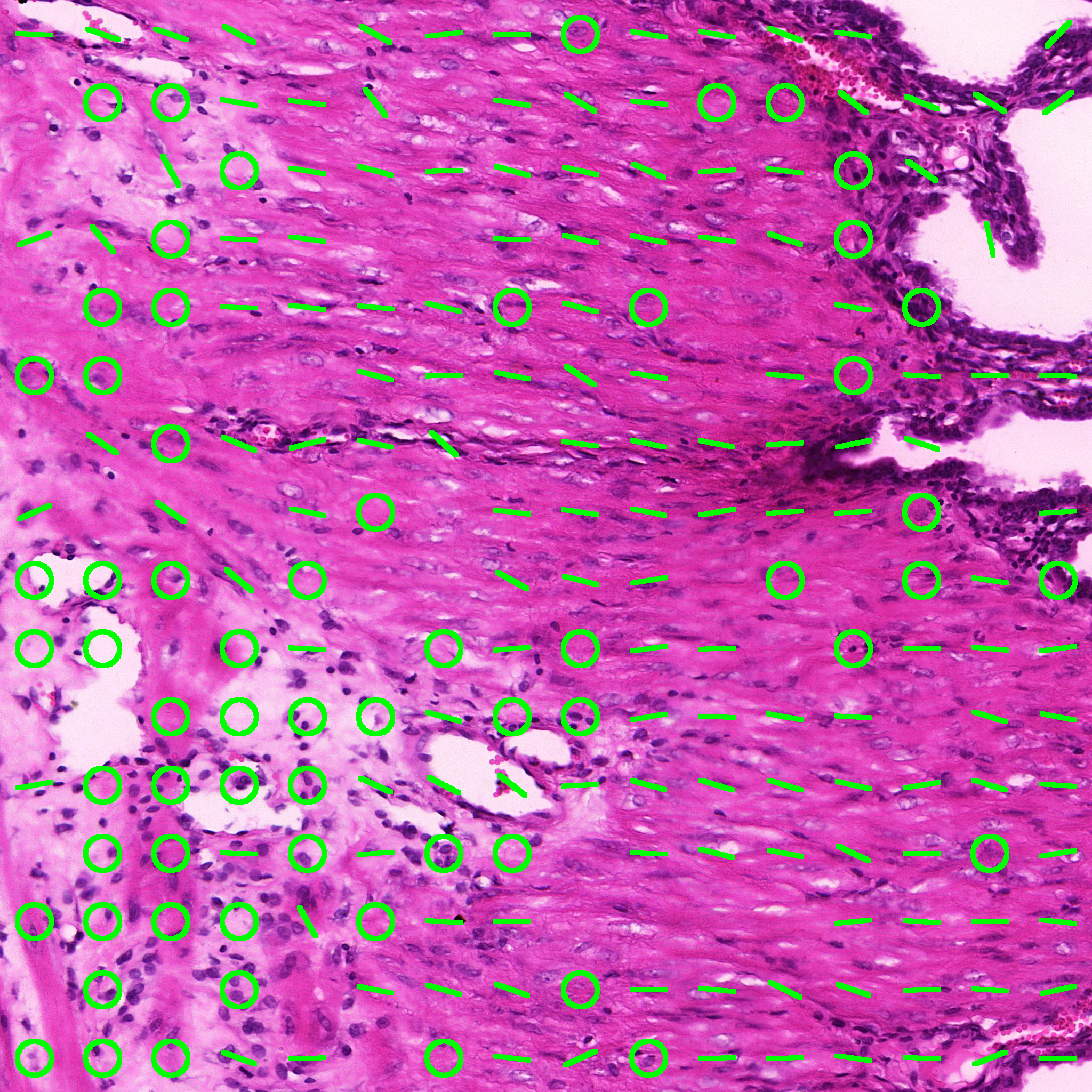

Supplement: S14 Fig — Image dimensions: 760 μm × 760 μm, stained with haemotoxylin and eosin. (TIFF) [file pone.0173404.s014.tiff]

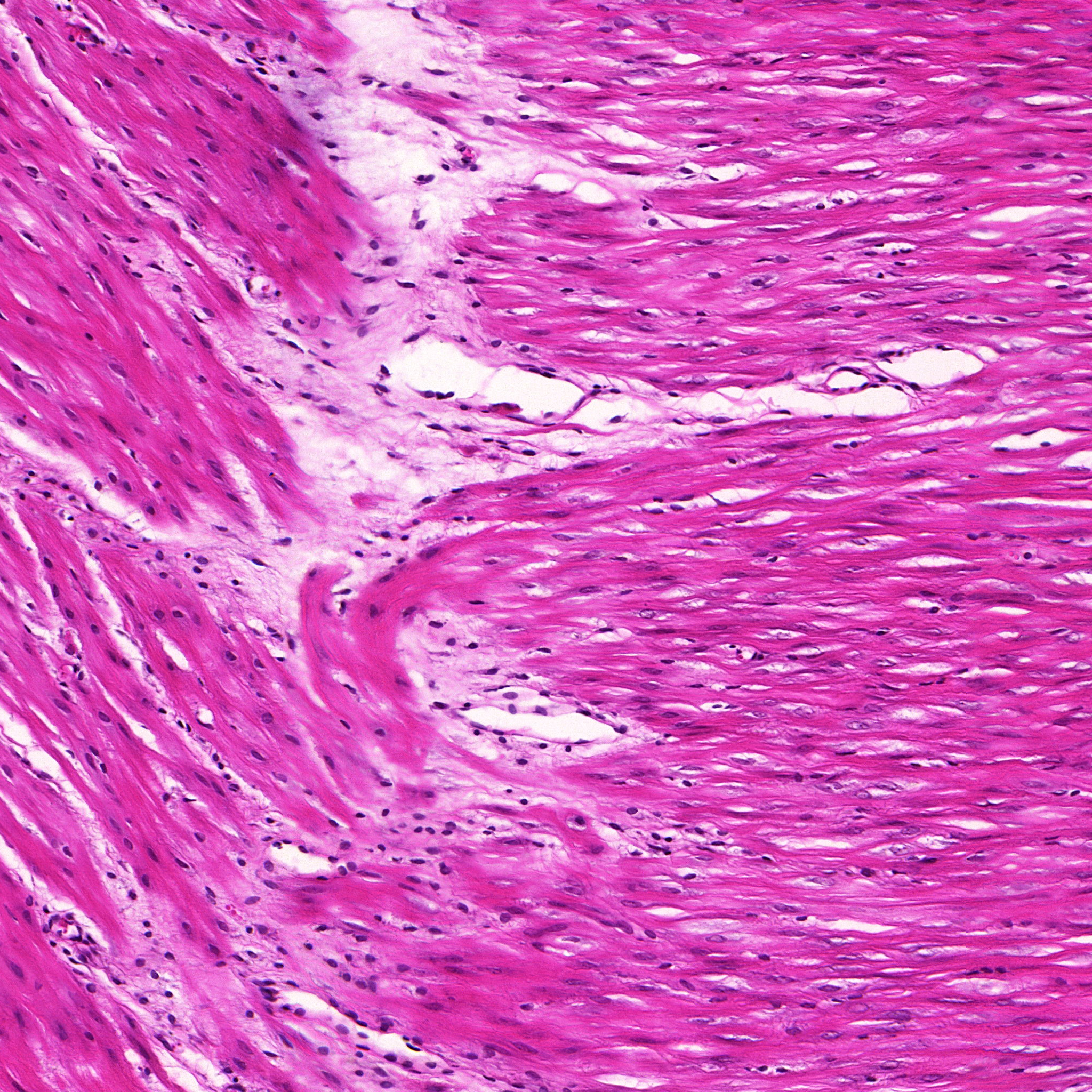

Supplement: S15 Fig — Image dimensions: 760 μm × 760 μm, stained with haemotoxylin and eosin. (TIFF) [file pone.0173404.s015.tiff]

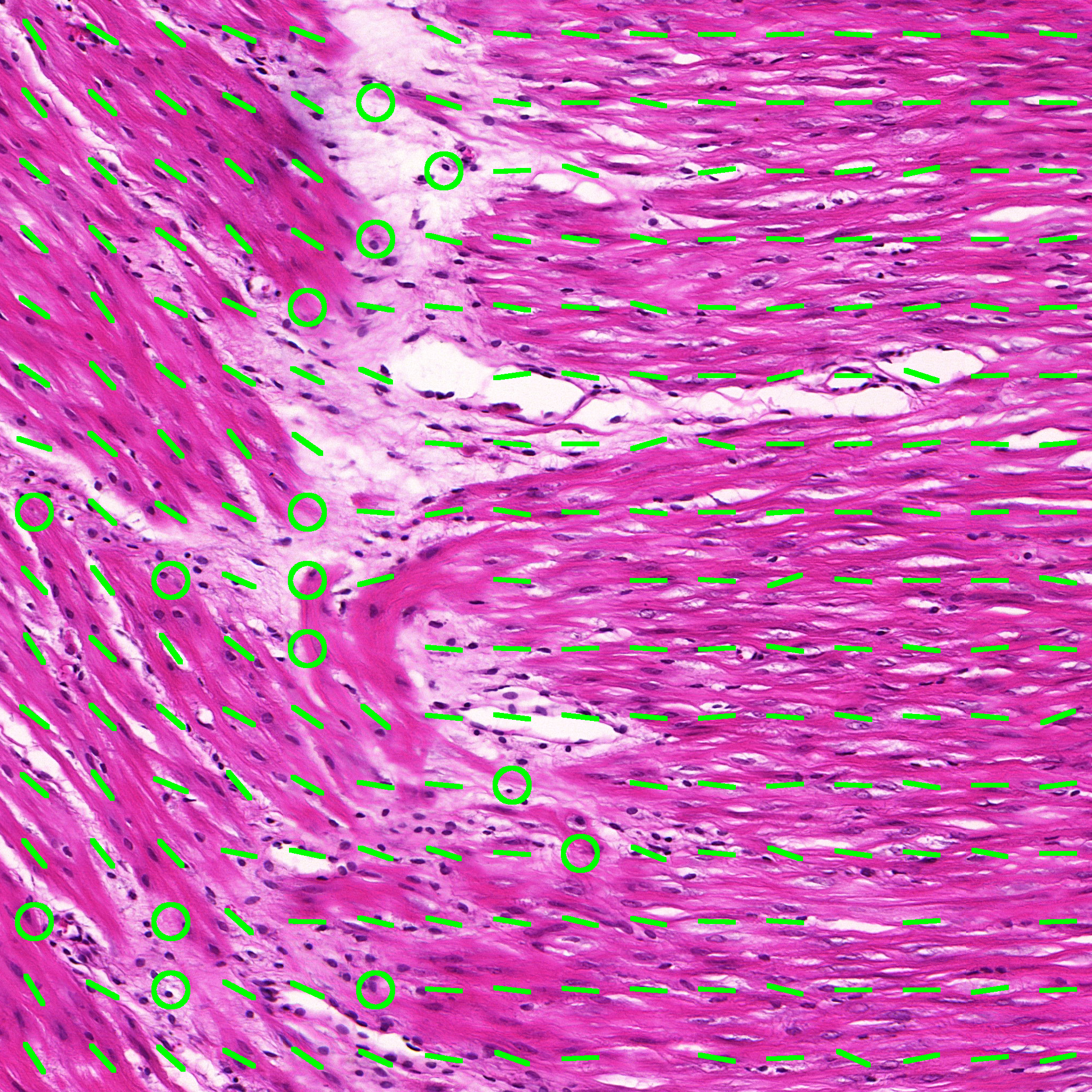

Supplement: S16 Fig — Image dimensions: 760 μm × 760 μm, stained with haemotoxylin and eosin. (TIFF) [file pone.0173404.s016.tiff]
