# Supplementary material for: A computational method for three-dimensional reconstruction of the microarchitecture of myometrial smooth muscle from histological sections
Source: PLoS One. 2017 Mar 16;12(3):e0173404. doi: 10.1371/journal.pone.0173404 (PMC5354307; doi:10.1371/journal.pone.0173404)
Supplement: S1 Appendix — (PDF) [file pone.0173404.s019.pdf]

S1 Appendix: Visualisation tools for interpretation of three-dimensional architecture

Highlighting fibrous structures

It is important to be able to visualise fibrous structure in the myometrium to identify fasciculi, sheets, and bundles, as previously observed by Young and Hession [6]. While the *in silico* reconstruction of the myometrium contains these structures, they are not readily observed in three dimensions, because they are not present in isolation, but, rather, form an intricate network of fibrous structures which are not readily separated by visual inspection. In order to identify these structures, portions of the reconstruction were highlighted in a manner that represents the local fibre direction.

Three forms of highlighting were used: bundle highlighting, which isolates thin strands of the reconstruction; fasciculus highlighting, which isolates wider strands than the bundle highlighting; and sheet highlighting, which isolates flat sheet-like structures. These three highlighting methods were used together to identify the relevant structures, while bundle and fasciculus highlighting were also used in isolation to view these structures throughout the tissue. When combining the three methods, the highlighting for a given point depended on the local structure of the tissue, as illustrated in Fig 1. Each point was classified based on this local structure, and for each classification a specific set of highlighting points was allocated. These highlighting points were used as a basis for a recursive expansion of highlighting from a point. Parameter values for the following are given in Table 1.

Categorising structures

The local structure of the tissue was determined by measuring line lengths along direction vectors, as follows. For a point p and unit direction vector v, define $\hat{v}$ to be

$$\hat{v} := v / \max_{i \in \{1,2,3\}} v_i,$$

where v_i is the ith component of v. The length of the line $L_+(p, v)$ along the vector v from p is defined to be the largest value $l_1 < l_{\max}$ such that for all integers $l \in [0, l_1]$, the weight of the voxel containing the point

$$p + l\hat{v}$$

is above the minimum weight $w_{\min}$ given in Table 1, where $l_{\max}$ is the length limit given in Table 1. The length $L(p, v) \in [0, 2l_{\max}]$ is thus defined

$$L(p, v) := L_+(p, v) + L_-(p, v),$$

where $L_-(p, v) = L_+(p, -v)$ is the length in the reverse direction.

Each point in the tissue was classified based on $L(p, v)$ as follows. For a point p with direction vector v, a sequence $\{v_i\}_{i=0}^{7}$ of vectors perpendicular to v was selected such that the angle between v_i and v_{i+1} was 22.5° for each $i < 7$. The length $L(p, v_i)$ was measured for each i, and compared to $L(p, v)$, using the threshold ratio T given in Table 1:

(i) if $L(p, v_i) \leq TL(p, v)$ for all i or $L(p, v) < l_{\min}$ (Table 1), p is classified as a *bundle point*;

(ii) if $L(p, v_i) > TL(p, v)$ and $L(p, v_{i+4}) > TL(p, v)$ for some $i < 4$, p is classified as a *fasciculus point*;

(iii) otherwise p is classified as a *sheet point.*

Classification (i) is used to identify points that are contained within thin or short structures (Fig 1**A**), while classification (ii) identifies points that are part of large structures with a roughly cylindric shape (Fig 1**B**). Classification (iii) implicitly identifies points in large structures which

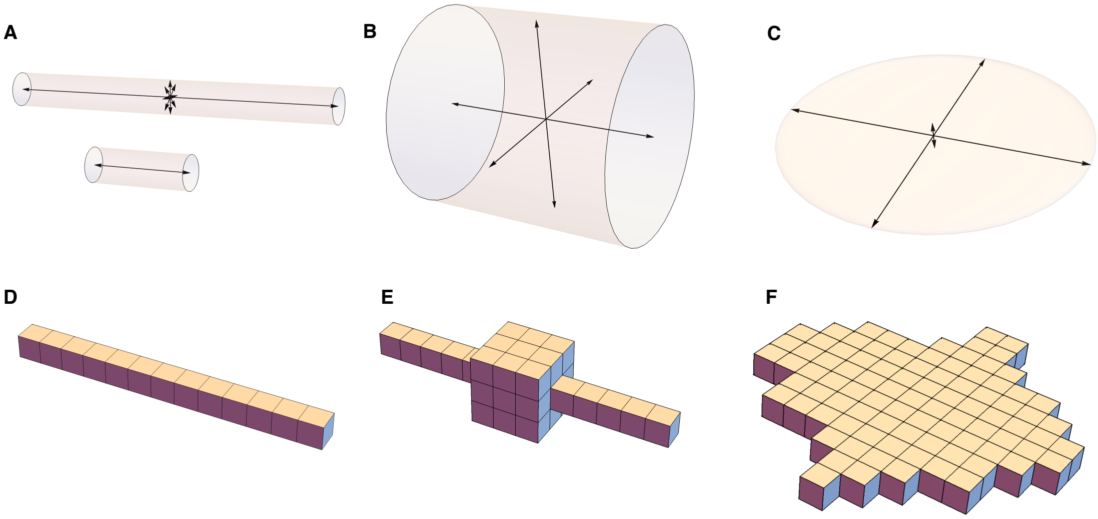

Figure 1: Classifying points as bundle, fasciculus, and sheet points. **A**: Local structure of *bundle points*, characterised by either the fibre width in all directions being much lower than the length or by a short length. Short structures are automatically included in this definition because characterisation of the structure based on length-to-width ratio is unreliable due to the short length, and bundle points have the smallest highlighting set (**D**), which reflects the small nature of the structure. **B**: Local structure of *fasciculus points*, characterised by large fibre width in two perpendicular directions relative to the fibre length, giving the impression of a large roughly cylindric structure. **C**: Local structure of *sheet points*, characterised by large fibre width in only one direction relative to the fibre length. **D**: Area around a *bundle point* to be highlighted, given by a line along the fibre direction. **E**: Area around a *fasciculus point* to be highlighted, given by the union of a bundle line (**D**) and the second order neighbourhood of the point. **F**: Area around a *sheet point* to be highlighted, given by the expanding the width of the bundle line (**D**) at each point within the plane shown in **E**.

are narrow in all but two directions (Fig 1**C**). Each of the above classifications is assigned a set of highlighting points associated with the local structure: bundle points are assigned a line of points along the directions v and$-v$ such that all points in the line have weight above w_{min} and the line extends at most $\hat{l}$ (Table 1) from the original point (Fig 1**D**). Fasciculi are assigned sets based on this bundle line: fasciculus points are assigned the union of this line and the second-order neighbourhood of p (Fig 1**E**). Sheet points are assigned the set of points based on the bundle line and the vector u perpendicular to v corresponding to the longest length L: the bundle line is widened along u up to a radius $\hat{l}$ such that the width is non-increasing width distance from the sheet point. An example of a sheet set is shown in Fig 1**F**.

Parameter	Value
l_{max}	10 voxel lengths
l_{min}	8 voxel lengths
$\hat{l}$	6 voxel lengths
w_{min}	0.1

Table 1: Parameter values for highlighting structures. One voxel length is approximately 50 μm, which means that $2l_{\text{max}}$, the upper limit on length measurements for identifying structures, is approximately 1 mm, which is in the range of widths previously observed for fasciculi [2, 6]. Weight is a dimensionless representation of nuclear density and local homogeneity with range $[0, 1]$.

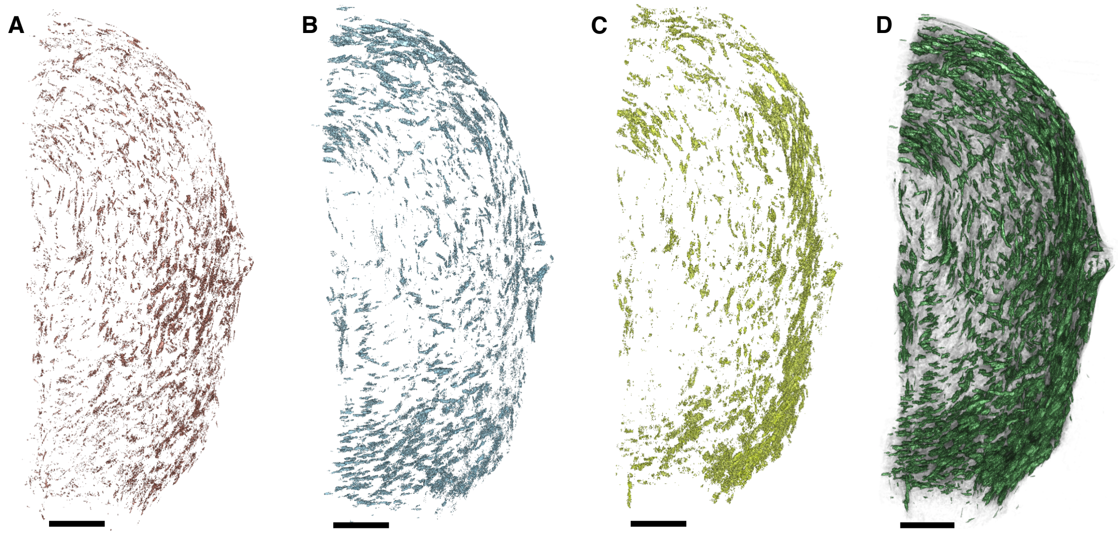

Figure 2: The effect of various forms of highlighting A: Highlighting of bundle points; **B**: Highlighting of fasciculus points; **C**: Highlighting of sheet points; **D**: Highlighting of all three structures. The highlighting category of a point depends on the local structure. The fine structures formed from highlighting bundle points (**A**) are evenly distributed in the tissue. Fasciculus points form larger fibrous structures (**B**), and show similar spatial distribution to bundles. Sheet points (**C**) are most dense toward the perimetrium (right in the image).

Random seeding

If all structures are rendered, the result is a nearly solid mass. To obtain a graphic that conveys more insight, a randomly selected portion is visualised. This selection is performed by selecting seed points at random and highlighting neighbouring points in a recursive fashion, utilising the previously assigned recursion sets (Fig 1).

For the purpose of selecting seed points, the reconstruction was divided into cuboid volumes, and in each subvolume a randomly selected voxel was taken as a seed point. If this voxel was weighted below the minimum weighting, then a new voxel was randomly selected, and this process of reselection was repeated up to 5 times. Highlighting outward from this seed point p_0 was performed to obtain a set of highlighted points p_i using the above assigned sets of points in the following recursive algorithm:

(i) highlight p_i;

(ii) obtain assigned set of highlighting points S_i for p_i;

(iii) repeat for all points in S_i.

The number of iterations of this algorithm was bounded to constrain the amount of tissue highlighted. For highlighting of only fasciculi or bundles, the set S_i in step (ii) is defined to be the fasciculus or bundle highlighting set for all points. The dimensions of subvolumes used for selecting seed points and bound on recursion varied depending on the type of highlighting being performed, as given in Table 2. The result of these algorithms can be seen in Fig 2.

Network graphs

Whereas the visualisation tools described above are geared to assess a faithful micro-anatomical representation, it may sometimes be desirable to visualise the underlying topology if the tissue, in particular, its connectivity structure. This network model of the tissue is generated by using a watershed algorithm in a similar manner to that used for the segmentation of the tissue presented

Parameter	bundle highlighting	fasciculus highlighting	combined highlighting
Subvolume x-length	10	30	30
Subvolume y-length	10	30	30
Subvolume z-length	3	10	10
Recursion bound	3	8	4

Table 2: Parameter values for different highlighting types. All lengths are in voxel lengths (~ 50 μm). Smaller subvolumes were used in bundle highlighting because the structures were finer.

in **Phase VII**. In this case, however, the structures isolated by the watershed algorithm are used to represent nodes of the network, with watershed boundaries representing the connections between these nodes. This network representation provides a visualisation of the tissue which enables the inspection of connectivity between fibres.

The three-dimensional scalar image used as the basis for the watershed algorithm is the weighting image generated in **Phase IX**. This image has already been segmented to delineate borders between tissue areas that are not deemed to be electrophysiologically connected To coarse grain the connected areas, a standard watershed segmentation algorithm was applied to this image, giving a set of isolated areas in the reconstruction that correspond to nodes in the network representation. The arcs of the network representation represent connections between these areas: if two segmented areas shared a watershed boundary, then an arc was drawn between the corresponding nodes. This process enabled the generation of a basic network representation of the tissue.

Each node in this network corresponds to a cluster of voxels in the reconstructed tissue. For each node the size, centre of mass, and mean direction of the corresponding cluster were recorded. Each connecting arc was assigned a weight equal to the largest weight value in the set of border voxels between the two clusters connected by the arc. These measurements enabled the visualisation of orientation of the connections relative to fibre direction: the mean direction at two nodes connected by an arc represents the fibre direction along that arc, which can be compared to the direction of the arc to determine how close this arc is to the fibre direction. If an arc was closer than 45° to the fibre direction, then it was classified as being along the fibre direction. Otherwise it was classified as being perpendicular to the fibre direction. This classification presents the network as a set of short straight line segments that either represent fibres (shown in red in Fig 3) or connections (shown in blue in Fig 3) between them. Additionally, the weighting of each arc represents the strength of the connection.

Simulating DT MRI

Diffusion tensor magnetic resonance imaging (DT MRI) is often used to determine fibre direction and anisotropy of smooth muscle tissue [4, 5, 7]. The reconstructions presented here specifically represent fibre direction, which can be used to simulate DT MRI scans of the reconstructions and compare them to previous findings. Diffusion tensors represent three-dimensional diffusion, given as a symmetric positive-definite matrix D, with principal eigenvector along the fibre direction, and eigenvalues representing relative diffusion along the eigenvectors. Diffusion tensors for individual voxels of the reconstruction were constructed with principal eigenvector equal to the previously calculated fibre direction, and eigenvalues estimated based on previous diffusion simulations in the myometrium [3], given in Table 3. These estimated eigenvalues are applied uniformly to all voxels, and therefore the fractional anisotropy is also uniform in this representation. To simulate the effects of DT MRI, this representation was coarse-grained to obtain diffusion tensors and fractional anisotropy at a similar resolution to previous DT MRI measurements [5]. The reconstruction was divided into cubes with side 8 voxels (380 μm) and the diffusion tensors were computed for each of these cubes. Conventional DT MRI computes diffusion tensors by measuring the reduction of intensity in resonance images when a range of diffusion gradients are applied to the volume [1].

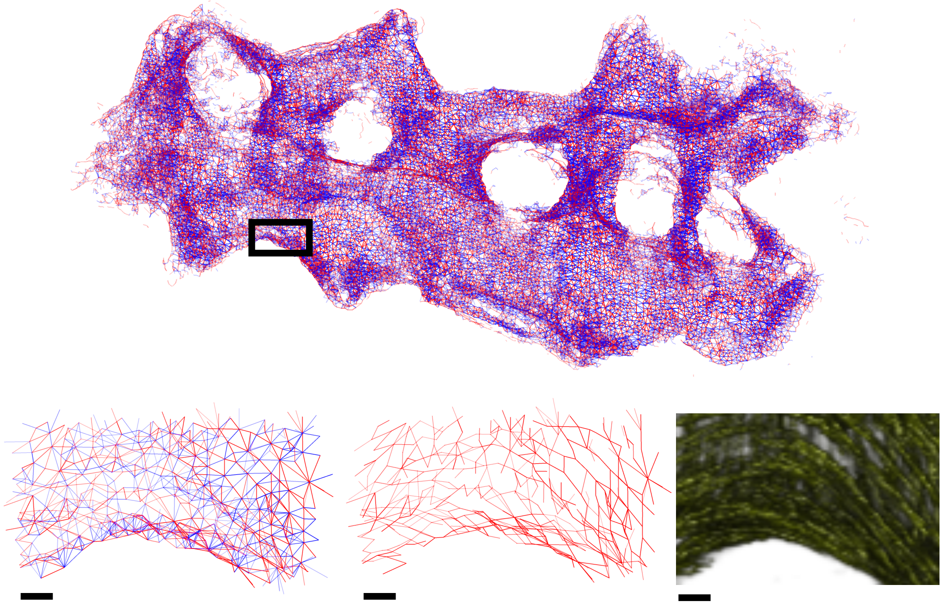

Figure 3: An example of a network graph of the whole tissue. Red arcs represent connections along the fibre direction, blue arcs represent connections away from the fibre direction, opacity represents the weighting of the arcs, nodes of the graph are omitted for ease of visualisation. Top: full network graph of a rat uterus. Bottom left: detailed view of the box shown in the top panel. Bottom middle: the same portion with just the arcs corresponding to fibres shown. Bottom right: bundle visualisation of the same portion of tissue. The arcs representing fibres in the network graph show direct correspondence with the visible longitudinal bundles in the reconstruction, while also showing arcs representing circular bundles underneath. Blue arcs connecting these bundles (bottom left) show numerous putative connections along the length of the bundles. This is true for the tissue in general, as can be seen by the high proportion of blue arcs in the top panel.

For an initial intensity S_0, the intensity of each diffusion gradient image S_k is given by

$$S_k = S_0 e^{b v_k^T D v_k},$$

where b is a constant set to 1 for the purposes of this simulation, v_k is the diffusion gradient vector applied to obtain S_k, and D is the diffusion tensor to be found. This is a system of linear equations that can be solved for D given an initial intensity image S_0 with no diffusion and 6 images S_k with diffusion gradient vectors v_k. The gradient vectors selected for simulation here are given in Table 3. The initial intensity S_0 of a cube containing the set of weights $\{w_i\}$ is given as

$$S_0 = \sum_i w_i.$$

The reduced intensity image S_k for a cube with direction vectors $\{v_i\}$ and corresponding weights $\{w_i\}$ is given by

$$S_k = S_0 - \sum_i w_i(\lambda_l v_i \cdot v_k + \lambda_t(1 - (v_i \cdot v_k)^2)^{1/2}),$$

where λ_l and λ_t are given in Table 3. The diffusion tensors obtained from solving the equations associated with these values are shown in Fig 4. These diffusion tensors are at a resolution ($\sim$

Parameter	Value
λ_l	0.7
λ_t	0.3
v_0	$(1, 1, 0)$
v_1	$(1, 0, 1)$
v_2	$(0, 1, 1)$
v_3	$(-1, 1, 0)$
v_4	$(-1, 0, 1)$
v_5	$(0, -1, 1)$

Table 3: Parameter values for simulating DT MRI. The values λ_l and λ_t are the estimated eigenvalues of the diffusion tensor in each voxel along the longitudinal and transverse directions respectively.

400 μm) that is comparable to conventional DT MRI, and represent the local diffusion based on estimated diffusion tensors in the original reconstruction. Additionally, the fractional anisotropy of a diffusion tensor [1] is given by:

$$\mathrm{FA} = \sqrt{\frac{3}{2}\frac{(\lambda_1 - \langle\lambda\rangle)^2 + (\lambda_2 - \langle\lambda\rangle)^2 + (\lambda_3 - \langle\lambda\rangle)^2}{\langle\lambda\rangle}}\ ,$$

where λ_i are the eigenvalues of the diffusion tensor and

$$\langle\lambda\rangle = \lambda_1 + \lambda_2 + \lambda_3$$

These methods enable the comparison of the reconstructed tissue with DT MRI scans of similar tissue samples.

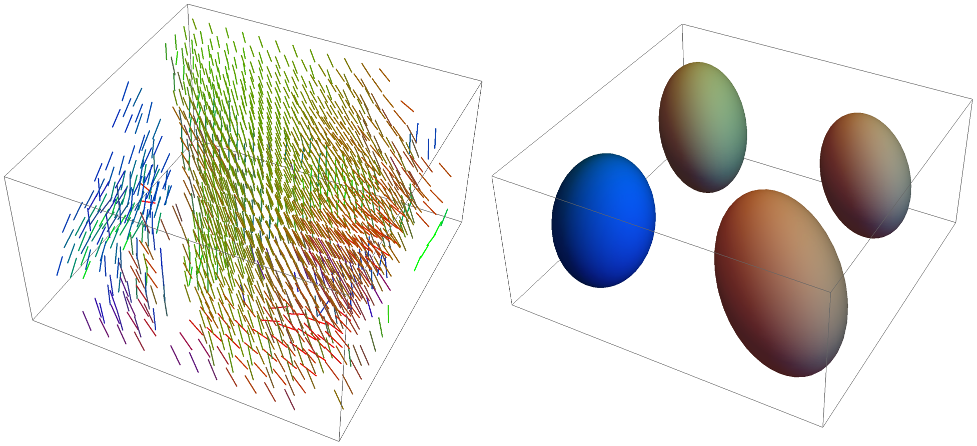

Figure 4: An example of diffusion tensors found by simulating DT MRI. Left: Direction vectors in a 760 μm$\times$760 μm$\times$380 μm portion of the reconstructed tissue, pseudocoloured so that each of red, green, and blue represent orthogonal directions. Right: The diffusion tensors resulting from simulating DT MRI in the volume on the left, using the same colouring as on the left.

References

[1] P. J. Basser. Inferring microstructural features and the physiological state of tissues from diffusion-weighted images. *NMR in Biomedicine*, 8(7):333–344, 1995.

[2] K. Goerttler. Die Architektur der Muskelwand des menschlichen Uterus und ihre funktionelle Bedeutung. *Jahrbuch Morph u Microsk Anat*, 65:45–128, 1931.

[3] P. S. La Rosa, H. Eswaran, H. Preissl, and A. Nehoraj. Multiscale forward electromagnetic model of uterine contractions during pregnancy. *BMC Medical Physics*, 12(4):1–16, 2012.

[4] S. Sinha and U. Sinha. In vivo diffusion tensor imaging of the human prostate. *Magnetic resonance in medicine*, 52(3):530–537, 2004.

[5] S. Weiss, T. Jaermann, P. Schmid, P. Staempfli, P. Boesiger, P. Niederer, R. Caduff, and M. Ba. Three-dimensional fiber architecture of the nonpregnant human uterus determined ex vivo using magnetic resonance diffusion tensor imaging. *The Anatomical Record Part A: Discoveries in Molecular, Cellular, and Evolutionary Biology*, 288(1):84–90, 2006.

[6] R. C. Young and R. O. Hession. Three-dimensional structure of the smooth muscle in the term-pregnant human uterus. *Obstetrics & Gynecology*, 93(1):94–99, 1999.

[7] L. Zhukov and A. H. Barr. Heart-muscle fiber reconstruction from diffusion tensor mri. In *Proceedings of the 14th IEEE Visualization 2003 (VIS'03)*, page 79. IEEE Computer Society, 2003.
